# Spatial Phylogenetics Reveals Endemism Hotspots and Conservation Priorities in Chinese Asteraceae

**DOI:** 10.1002/ece3.72403

**Published:** 2025-11-02

**Authors:** Xinyi Zheng, Xinyu Chen, Tianmeng Qu, Yanru Zhang, Yizhen Shao, Bing Zhang, Zhixi Fu, Xiaoxia Zhang

**Affiliations:** ^1^ Key Laboratory of Land Resources Evaluation and Monitoring in Southwest Sichuan Normal University, Ministry of Education Chengdu China; ^2^ College of Life Sciences Sichuan Normal University Chengdu China; ^3^ College of Life Science Henan Agricultural University Zhengzhou China; ^4^ Sichuan Leshan Ecological Environment Monitoring Center Station Leshan China; ^5^ Sustainable Development Research Center of Resources and Environment of Western Sichuan Sichuan Normal University Chengdu China; ^6^ State Key Laboratory of Plant Diversity and Prominent Crop/State Key Laboratory of Systematic and Evolutionary Botany, Institute of Botany the Chinese Academy of Sciences Beijing China

**Keywords:** Asteraceae, China, phylogenetic diversity, phylogenetic endemism, spatial phylogenetics

## Abstract

The Asteraceae is recognized as the largest family of dicotyledonous plants globally. Despite being the most species‐rich plant family in China, a nationwide biodiversity assessment integrating phylogenetic approaches remains lacking—hindering the identification of conservation priority areas from an evolutionary perspective. Using six chloroplast markers (*mat*K, *ndh*F, *rbc*L, *rps*16, *psb*A‐*trn*H, and *trn*G) and the nuclear ribosomal ITS region, we reconstructed the phylogeny of Chinese Asteraceae. Combining spatial distribution data of 1092 Asteraceae species in China (representing 7 subfamilies, 22 tribes, and 215 genera), we mapped the distribution patterns of species richness, weighted endemism, phylogenetic diversity, and phylogenetic endemism. The results revealed exceptionally high biodiversity, phylogenetic diversity, and endemism in the Tianshan–Altai Mountains of northwestern China, the southwestern region (particularly the Hengduan Mountains), and the island of Taiwan. These areas are characterized by complex topography and/or climatic stability, preserving long independent evolutionary lineages that may harbor unique genetic and functional traits, thus demonstrating exceptional conservation value. This study provides the first preliminary evaluation of the biodiversity and spatial phylogenetics of Chinese Asteraceae using phylogenetic methods, identifying biodiversity and endemism hotspots deserving conservation priority. We also established an initial spatial phylogenetic dataset for Chinese Asteraceae, which will facilitate future research on biogeographic history, trait evolution, and the development of conservation strategies.

## Graphical abstract

Based on the phylogenetic method and the spatial distribution data of 1092 Asteraceae plants in China, this study conducted a preliminary evaluation on the biodiversity and spatial phylogeny of Asteraceae plants in China for the first time and determined the biodiversity and endemic hotspots worthy of priority protection.

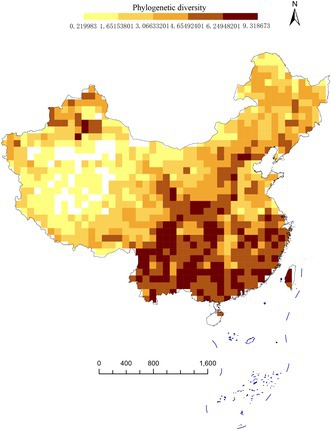

## 1 Introduction

Asteraceae, the largest eudicot family globally with approximately 24,700 species across 1627 genera, represents a cornerstone for understanding angiosperm evolution and ecology (Fu et al. 2016; Chen and Wang 2025). According to the Angiosperm Phylogeny Group IV (APG IV) classification system, this family is placed in Eudicots–Asterids–Campanulids–Asterales (Chase et al. 2016). Originating in Patagonia (Palazzesi et al. 2009; Barreda et al. 2010; Palazzesi and Barreda 2012), Asteraceae subsequently dispersed to all continents worldwide except Antarctica (Palazzesi et al. 2022). Nowadays, the family occupies almost all environments, from lowland forests to high alpine fell fields. Life forms are also extremely variable, including herbs, succulents, lianas, epiphytes, trees, or shrubs (Shi et al. 2011). Moreover, Asteraceae also has considerable scientific, ecological, and economical value, providing food crops, ornamentals, and herbal medicines (Fu et al. 2016; Rolnik and Olas 2021; Chierici et al. 2025). Due to the medicinal uses of hypolipidemic, antioxidant, antihypertensive, and vasorelaxant properties (Michel et al. 2020), many species have been used in traditional medicine for a long time, such as *Achillea arabica* Kotschy, *Ageratum conyzoides* L., and *Artemisia campestris* L. Altogether, these characteristics make this family an excellent group to study biogeography (Shi et al. 2011), pollen evolution (Qu et al. 2025), secondary chemistry (Hull‐Sanders et al. 2009), paleopolyploidy (Barker et al. 2008), domestication (Dempewolf et al. 2008), and invasions (Zhao et al. 2012). Asteraceae is the most species‐rich family in China (Fu et al. 2016).

Chinese Asteraceae comprise approximately 2533 indigenous species (ca. 1145 endemic species) and 296 genera (nearly 17% of the world's genera) (Shi et al. 2011; Chen and Wang 2025). Consequently, understanding the assembly and distribution of Chinese Asteraceae is critical to deciphering the broader evolutionary history of the region's flora. However, existing studies have primarily focused on either global phylogenetic frameworks (Mandel et al. 2019) or regional taxonomic revisions, creating a critical disconnect. This gap is particularly evident in the lack of a comprehensive, phylogenetically informed biodiversity assessment for Asteraceae across China. While studies in other regions, such as Australia (Schmidt‐Lebuhn et al. 2015) and islands (Baldwin and Sanderson 1998), have successfully utilized spatial phylogenetics to identify evolutionary hotspots and conservation priorities, a parallel synthesis for the Chinese continent remains absent (Fu et al. 2016; Huang et al. 2016; Panero and Crozier 2016; Mandel et al. 2019; Zhang, Huang, et al. 2021; Zhang, Wang, et al. 2021; Palazzesi et al. 2022; Omollo et al. 2024).

This gap is problematic because traditional conservation planning, which relies heavily on species‐level metrics like species richness (SR), fails to capture the evolutionary dimension of biodiversity (Faith 1992; Winter et al. 2013). Treating all species as equivalent overlooks the stark contrast between evolutionarily distinct “relict” lineages, whose loss would be irreplaceable, and rapidly radiating groups comprising many recently diverged species (Redding and Mooers 2006; Zhao et al. 2019; Baldwin and Sanderson 1998).

The phylogenetic diversity (PD) framework addresses this limitation by quantifying the totality of evolutionary history represented in a community (Faith 1992). When integrated with geographic range data into measures like phylogenetic endemism (PE), it allows for the identification of areas that harbor not only unique species but also unique evolutionary history (Rosauer et al. 2009). Therefore, four indices are used to measure relative endemism and diversity: species richness (SR), which quantifies the number of species per unit area (Gotelli and Colwell 2001; Magurran 2021); weighted endemism (WE), which combines the rarity of range size (Laffan et al. 2016); phylogenetic diversity (PD), which measures evolutionary uniqueness through the sum of branch lengths (Faith 1992); and phylogenetic endemism (PE), which combines phylogenetic relationships and geographical range restrictions (Rosauer et al. 2009).

Spatial phylogenetic methods have proven powerful in revealing patterns invisible to traditional approaches. In Asteraceae, spatial phylogenetic analyses have yielded significant insights (Mishler et al. 2020). Schmidt‐Lebuhn et al. (2015) applied the Categorical Analysis of neo‐ and paleo‐Endemism to assess phylogenetic diversity patterns in Australian Asteraceae at both generic and species levels. The research results of Asteraceae in Australia showed that there were similar phylogenetic diversity and endemic patterns at the genus level and species level. The neoendemic hotspots are mainly distributed in the mountainous areas in the southeast of Australia, while the hotspots in paleoendemic are in the tropical areas in the north. Similarly, research on island‐endemic Asteraceae (e.g., *A. sandwicense* ) employed Relative Phylogenetic Endemism (RPE) to differentiate between recent adaptive radiations (neoendemism) and relictual lineages (paleoendemism), fundamentally advancing island biogeography theory (Baldwin and Sanderson 1998). The methodology has demonstrated comparable significance in other plant lineages. For tropical trees, Dexter et al. (2017) integrated continental‐scale phylogenies with species distribution data to identify evolutionary refugia in Amazonia, while Thornhill et al. (2016) showed that > 20% of significant conservation areas in Australian flora would be overlooked using traditional species‐based approaches. In Gesneriaceae, Xu et al. (2019) revealed congruent patterns of phylogenetic diversity, phylogenetic endemism, and species richness in Primulina across karst regions of southern China. Their findings identified these areas as both “museums” preserving ancient lineages and “cradles” fostering recent diversification, highlighting the dual evolutionary role of karst habitats. These successes in other plant groups underscore the potential of such an approach for Asteraceae in China.

Here, we investigate the spatial patterns of Chinese Asteraceae using the largest and most comprehensive phylogenetic trees and the most extensive distribution data, with two aims: (1) to reveal the distribution patterns of phylogenetic diversity and endemism in Asteraceae; and (2) to identify priority conservation areas across multiple dimensions of Asteraceae biodiversity.

## 2 Methods

### 2.1 Reconstruct the Phylogenetic Tree

To investigate the evolutionary relationships among major lineages of Asteraceae in China and facilitate analyses of biodiversity and endemism, we reconstructed a preliminary phylogenetic tree of Chinese Asteraceae species. Through comprehensive evaluation of bootstrap values and topological structures derived from phylogenetic trees from individual or combined molecular markers, we identified an optimal molecular dataset for this study, comprising six chloroplast DNA regions (*mat*K, *ndh*F, *rbc*L, *rps*16, *psb*A‐*trn*H, and *trn*G) and the nuclear ribosomal internal transcribed spacer (ITS) sequence. Based on taxonomic identification and recent literature (Fu et al. 2016; Zhang, Huang, et al. 2021), we excluded species that failed to cluster with their putative relatives in the topological structure. This exclusion criterion was implemented because these taxa might represent cases of problematic taxonomic delimitation that could potentially confound analyses relying on current taxonomic frameworks (Li et al. 2019; Zhu et al. 2021).

The final dataset included 1092 Asteraceae species, compiled from both previous studies and the NCBI database. The sequence composition was as follows: 493 *rbc*L, 533 *mat*K, 282 *ndh*F, 1088 ITS, 481 *psb*A‐*trn*H, 198 *rps*16, and 147 *trn*G sequences (Table S1). Sequence alignment for the seven selected molecular markers was conducted using MAFFT v7.520 (Katoh and Standley 2013) with default parameters. The aligned sequences were subsequently processed using the “trimAL Wrapper” module in TBtools v2.322 to eliminate poorly aligned regions. Sequence concatenation was performed with SequenceMatrix 1.10, generating a combined dataset comprising 19,242 aligned nucleotide positions for phylogenetic reconstruction. Maximum likelihood (ML) phylogenetic analysis was executed in RAxML 8.0.24 in the CIPRES Science Gateway (Stamatakis 2014) under the GTRGAMMA model, with the following parameters: 1000 rapid bootstrap replicates for branch support estimation and 100 independent ML searches to identify the optimal tree topology. The resulting phylogenetic tree was visualized using FigTree v1.4.4 and subsequently utilized for biodiversity and endemism assessments.

### 2.2 Generate Topographic Map of China

The standard map of China was downloaded from the National Geomatics Center of China (http://www.ngcc.cn/). The GDEMV3 30 m resolution digital height data of China was downloaded from Geospatial Data Cloud (https://www.gscloud.cn/sources/accessdata/aeab8000652a45b38afbb7ff023ddabb?pid=302). The topographic map of China was created and generated using ArcGIS v.10.8.2 (https://www.esri.com/en‐us/arcgis).

### 2.3 Spatial Dataset Assembly

The spatial distribution data were compiled from a wide range of sources, including nearly all published national and provincial floras, as well as selected local floras, species checklists, and herbarium records. For the cleaning of distribution data, we adopted a series of procedures: Suspected erroneous records (such as mismatches between coordinates and the names of specimen collection locations) were manually verified using Google Earth and those that could not be validated were excluded; duplicate records were removed by matching specimen numbers; meanwhile, synonymies and spelling errors in species' scientific names were corrected to achieve unified and standardized nomenclature. Finally, we obtained 634,314 occurrence records for native Asteraceae species from 2087 species (accounting for approximately 82% of the total recorded Asteraceae species in China). The standard map of China was divided into 100 km × 100 km grid cells under equal area projection in ArcGIS v.10.8.2. To minimize the sampling bias of unequal sampling areas, we divided the map of China into 100 km × 100 km grid cells (945 grids in total to cover all of China), and grid cells on the border that cover less than 50% of the area of a grid cell (i.e., 5000 km^2^) were excluded from the analyses.

### 2.4 Spatial Phylogenetic Analysis

We performed basic biodiversity analyses using Biodiverse V4.1 software (Laffan et al. 2010), incorporating 100 km × 100 km gridded distribution data of 2087 native Asteraceae species in China and a relatively comprehensive phylogenetic tree of Chinese Asteraceae (including branch lengths for all analyzed species). Following the methods of previous studies (Mishler et al. 2014; Zhu et al. 2021; Omollo et al. 2024), we calculated four key biodiversity and endemism indices, namely species richness (SR), weighted endemism (WE), phylogenetic diversity (PD), and phylogenetic endemism (PE). All calculations were conducted at the grid‐cell level (945 grids in total to cover all of China). The results were visualized using ArcGIS v.10.8.2. In addition, SPSS 20 software was used to conduct nonparametric Spearman's rank correlation analysis and principal component analysis (PCA) on four biodiversity indices.

## 3 Results

### 3.1 Phylogenetic Tree of Asteraceae in China

**FIGURE 1:**
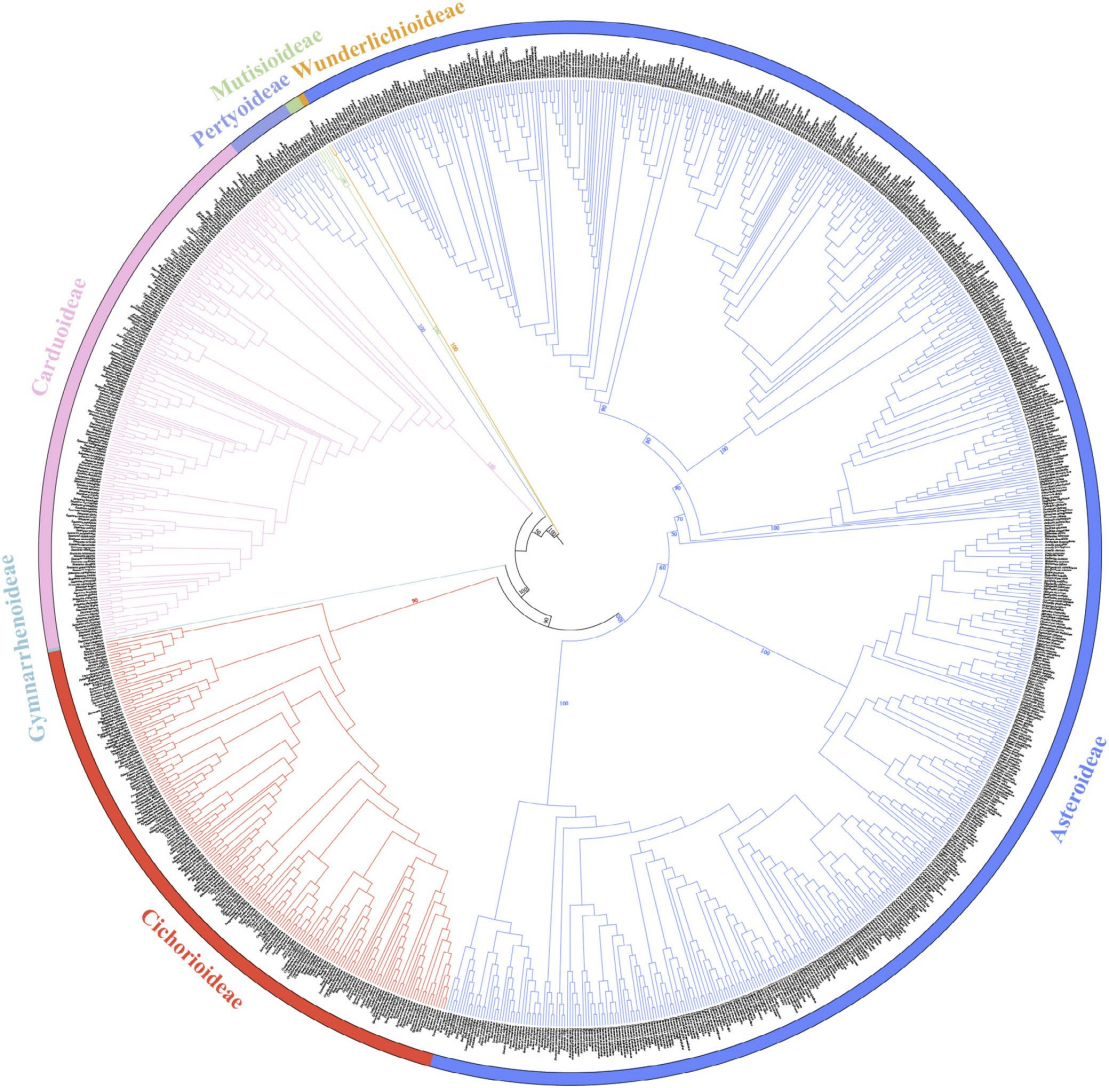
Phylogenetic tree of Asteraceae in China. The phylogenetic tree of Asteraceae in China includes 7 subfamilies (Mutisioideae, Wunderlichioideae, Carduoideae, Pertyoideae, Gymnarrhenoideae, Cichorioideae, and Asteroideae). Different branch colors represent different subfamilies.

The detailed topology of Chinese Asteraceae was investigated using the 215 genera (nearly 73% of genera in China) and 1092 species (nearly 43.11% of species in China) (Figure 1). In previous studies, Chinese Asteraceae were distributed in 7 subfamilies (Mutisioideae, Wunderlichioideae, Carduoideae, Pertyoideae, Gymnarrhenoideae, Cichorioideae, and Asteroideae) and 22 tribes (Mutiseae, Hyalideae, Cardueae, Pertyeae, Gymnarrheneae, Vernonieae, Cichorieae, Doroniceae, Senecioneae, Astereae, Anthemideae, Gnaphalieae, Calenduleae, Inuleae, Athroismeae, Helenieae, Coreopsideae, Neurolaeneae, Tageteae, Millieae, Eupatorieae, and Heliantheae) (Panero and Funk 2002, 2008; Funk et al. 2005; Fu et al. 2016; Susanna et al. 2020; Zhang, Huang, et al. 2021; Zhang, Yang, et al. 2024). This was consistent with our research results. See Figure 1 and Figure S1 for the results. The GenBank accession numbers for species sampled in this study are shown in Table S1.

### 3.2 Topographic Distribution of China

**FIGURE 2:**
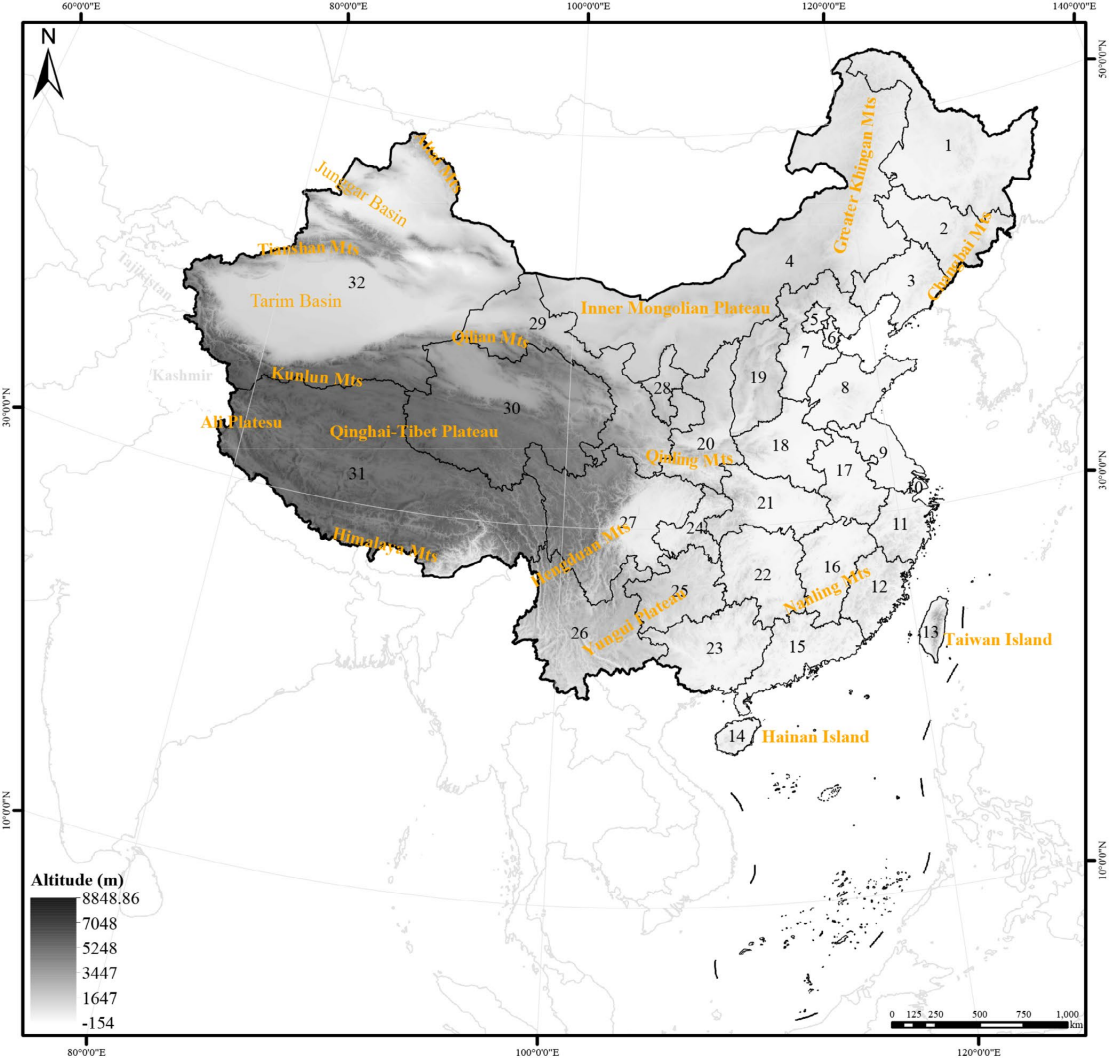
Topographic map of China showing locations mentioned in this study. 1 Heilongjiang, 2 Jilin, 3 Liaoning, 4 Inner Mongolia, 5 Beijing, 6 Tianjin, 7 Hebei, 8 Shandong, 9 Jiangsu, 10 Shanghai, 11 Zhejiang, 12 Fujian, 13 Taiwan, 14 Hainan, 15 Guangdong, 16 Jiangxi, 17 Anhui, 18 Henan, 19 Shanxi, 20 Shaanxi, 21 Hubei, 22 Hunan, 23 Guangxi, 24 Chongqing, 25 Guizhou, 26 Yunnan, 27 Sichuan, 28 Ningxia, 29 Gansu, 30 Qinghai, 31 Tibet, 32 Xinjiang. Altai Mts, Junggar Basin, Tianshan Mts, Tarim Basin, Kunlun Mts, Ali Plateau, Himalaya Mts, Qinghai–Tibet Plateau, Lhoka Prefecture, Qilian Mts, Inner Mongolian Plateau, Qinling Mts, Hengduan Mts, Yungui Plateau, Nanling Mts, Daxinganling Mts, Changbai Mts, Hainan Island, Taiwan Island.

This study focuses on the administrative regions of China as the research subject, thereby enhancing the practical applicability of conservation strategies for guiding the planning of protection gaps in nature reserves (Margules and Pressey 2000). A total of 32 provincial‐level administrative units in China are covered. The terrain of China descends from west to east, and the study mainly includes three altitude gradients (Ye 2020). High‐altitude regions, generally above 3500 m, reach a maximum elevation of 8848.86 m (Figure 2). The Qinghai–Tibet Plateau is bounded to the north by the Kunlun Mts (including the Karakoram Range, Yu et al. 2025) and the southern Qilian Mts (Shi et al. 2017), to the east by the Hengduan Mts (Gao et al. 2015; Xing Chen 2018), and to the south by the Himalayas (Figure 2; Yu et al. 2020). Mid‐altitude regions, with an average elevation of 1000 and 3500 m, encompass the Inner Mongolia Plateau, Loess Plateau, Yunnan–Guizhou Plateau, as well as the Tarim Basin, Junggar Basin, and Sichuan Basin. Low‐altitude areas, with an average elevation below 500 m, mainly consist of plains and hills. Additionally, the Qinling Mts, an eastern extension of the Kunlun Mts, traverse central China. The names of these regions and their corresponding geographical locations are provided in Figure 2.

### 3.3 Observed Patterns of Diversity and Endemism of Asteraceae

**FIGURE 3:**
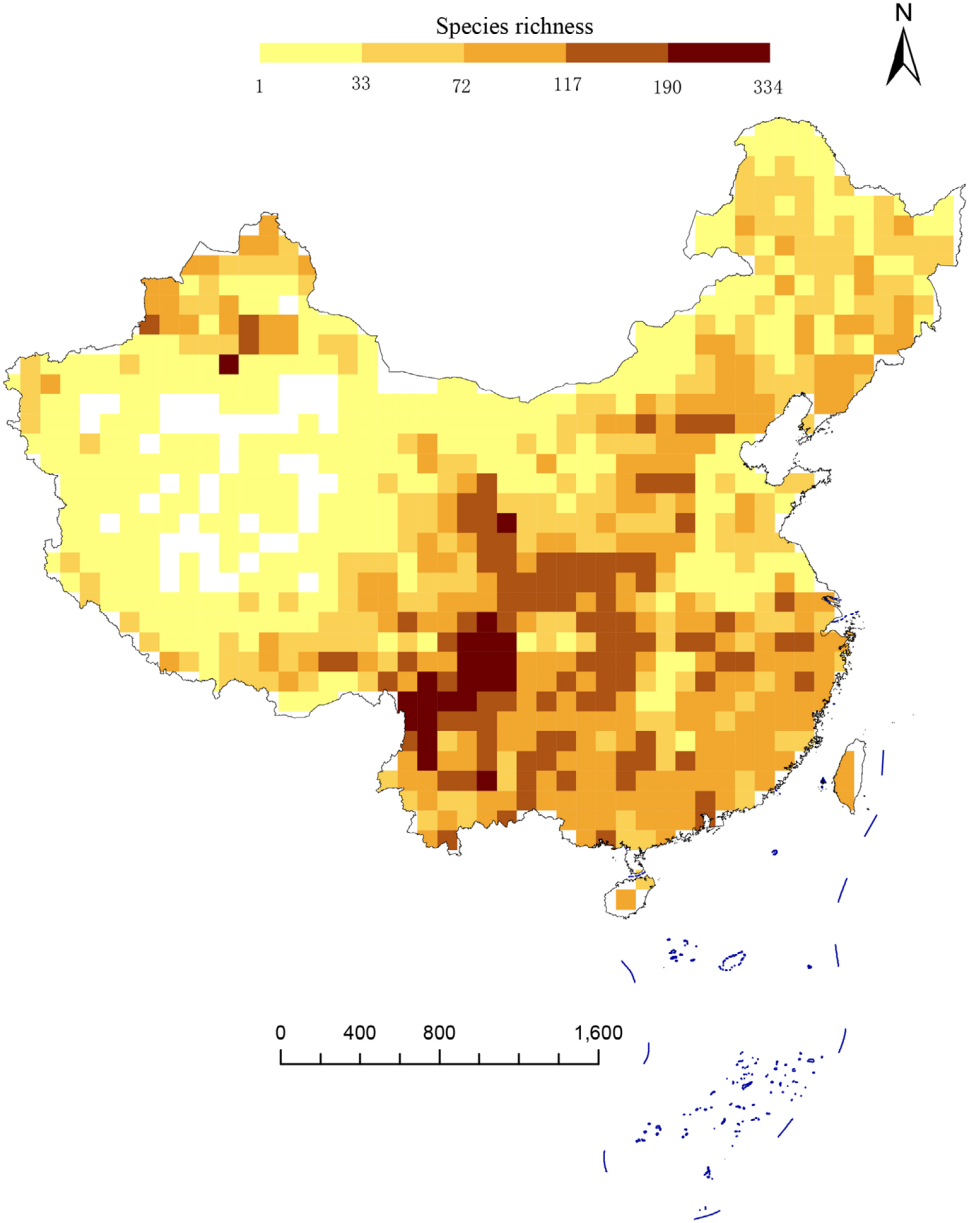
Maps showing the observed species richness of Asteraceae species in China. All of them are based on phylogram. A blank grid indicates that there is no record of Asteraceae species found in this place based on spatial distribution data.

**FIGURE 4:**
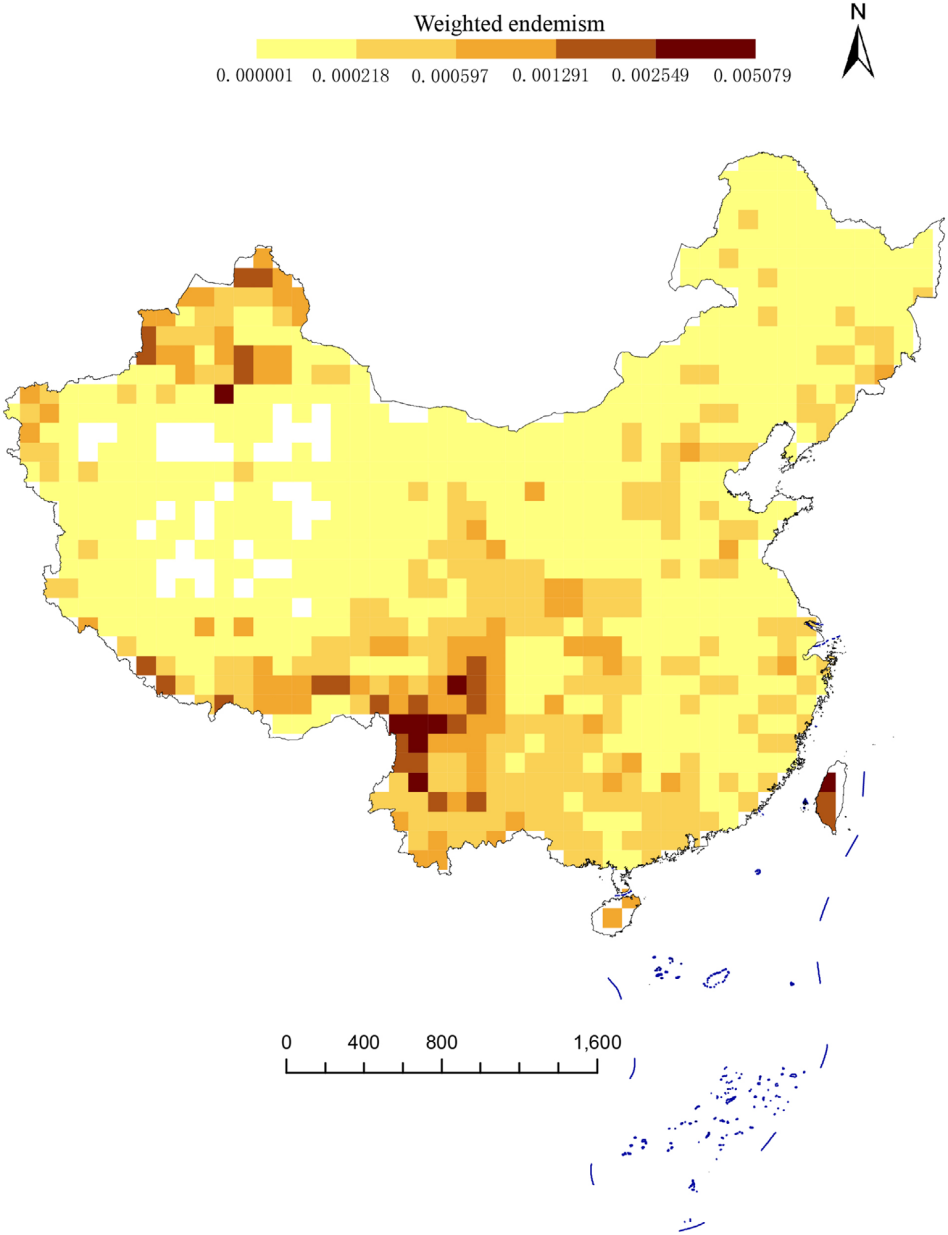
Maps showing the observed weighted endemism of Asteraceae species in China. All of them are based on phylogram. A blank grid indicates that there is no record of Asteraceae species found in this place based on spatial distribution data.

**FIGURE 5:**
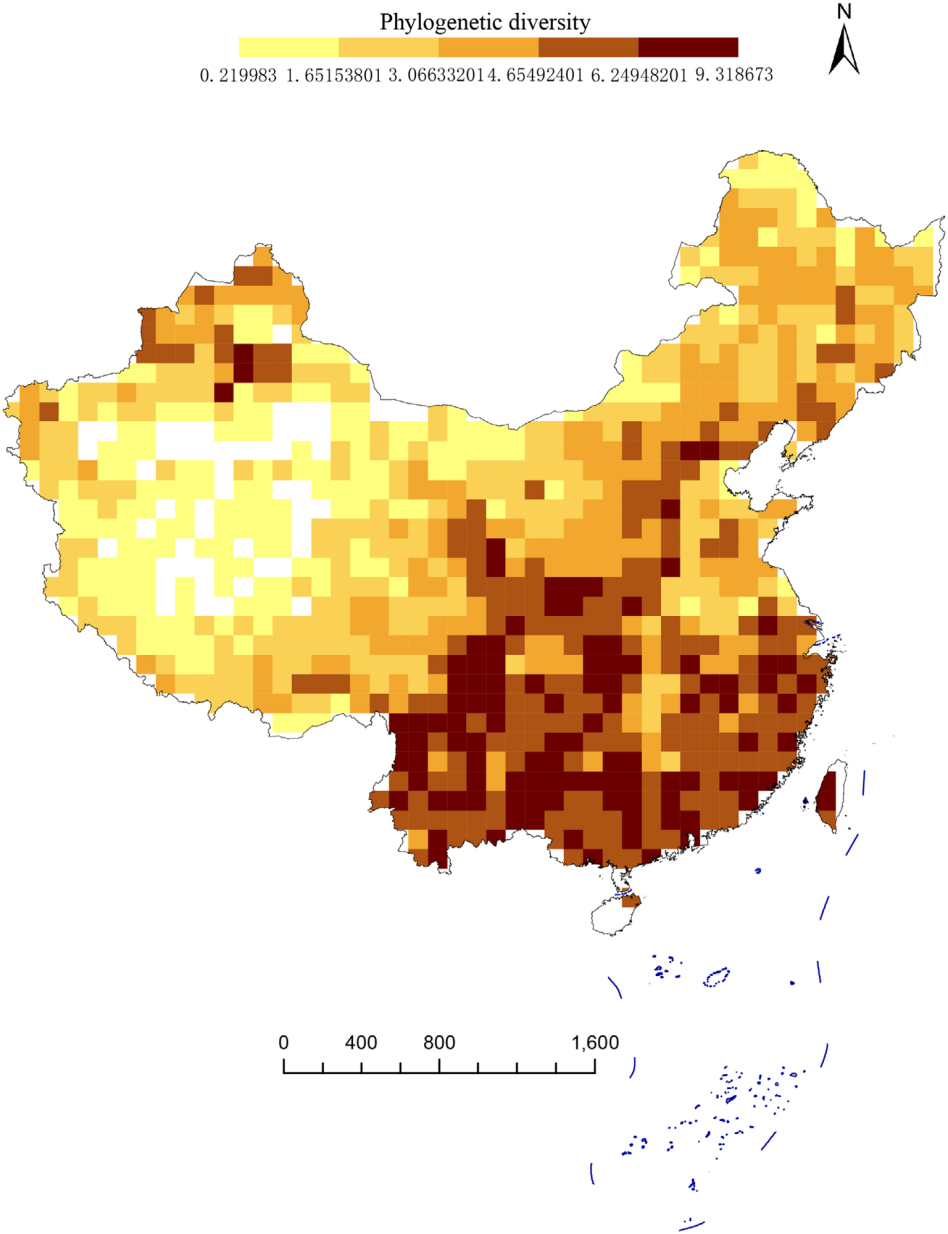
Maps showing the observed phylogenetic diversity of Asteraceae species in China. All of them are based on a phylogram. A blank grid indicates that there is no record of Asteraceae species found in this place based on spatial distribution data.

**FIGURE 6:**
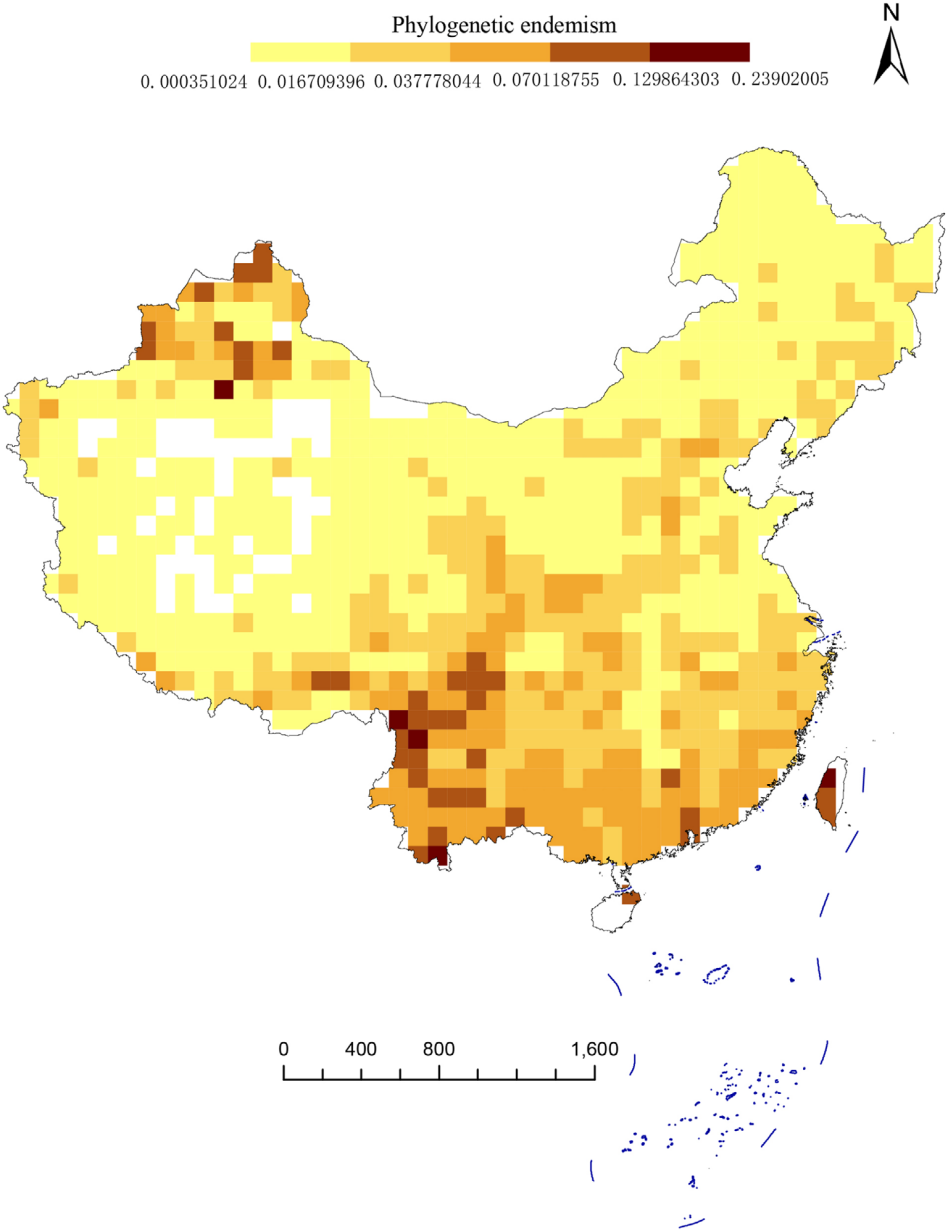
Maps showing the observed phylogenetic endemism of Asteraceae species of China. All of them are based on the phylogram. A blank grid indicates that there is no record of Asteraceae species found in this place based on spatial distribution data.

Our results reveal that Southwestern and Southern China exhibit the highest diversity and uniqueness of Asteraceae species, based on both traditional taxonomic metrics and phylogenetic indices—including species richness (SR), weighted endemism (WE), phylogenetic diversity (PD), and phylogenetic endemism (PE) (Figure 3). First, grid‐based species richness analysis (Figure 3) shows that higher richness is concentrated in the Tianshan Mts, Junggar Basin, Hengduan Mts, Qilian Mts, Yunnan–Guizhou Plateau, Hainan Island, Taiwan Island, eastern Sichuan, and central Gansu. In contrast, lower species richness occurs in the Qinghai–Tibet Plateau, Kunlun Mts, Tarim Basin, and Northeast China. Second, weighted endemism (WE) was calculated for each grid, with spatial patterns shown in Figure 4. The highest WE values are found in Northwestern China (Tianshan Mts, Junggar Basin, and Altai Mts) and Southern China (Hengduan Mts and Taiwan Island). Additionally, most regions south of the Qinling Mts in South China also exhibit relatively high WE. Notably, the spatial distribution of phylogenetic diversity (PD) closely mirrors that of species richness (Figure 5). Similarly, the pattern of phylogenetic endemism (PE) aligns with that of taxon‐based weighted endemism (Figure 6).

In addition, the results of the nonparametric Spearman's rank correlation analysis show that all the indices maintain a high degree of positive correlation (the Spearman's correlation coefficient ρ between SR and PD is 0.96, and the ρ between PE and WE is 0.90), confirming the consistency of the change trends of these indices (*p* < 0.01). The results of the principal component analysis (PCA) show that the KMO value is 0.586 (close to the acceptable threshold of 0.6), and Bartlett's test of sphericity is significant (χ^2^ = 3946.731, *p* < 0.001), indicating that the data are suitable for PCA. The variance contributions of each index are as follows: SR (81.963%, with an eigenvalue of 3.28), PD (12.954%, with an eigenvalue of 0.52), PE (3.99%, with an eigenvalue of 0.16), and WE (1.093%, with an eigenvalue of 0.044).

## 4 Discussion

This study highlights China as a global hotspot for Asteraceae diversity research. The unique phylogenetic and biogeographic patterns of Chinese Asteraceae not only reflect their distinct evolutionary trajectories but also carry profound ecological significance—reinforcing China's status as a critical region for understanding the family's diversification. Moreover, the combined use of species richness, weighted endemism, phylogenetic diversity, and phylogenetic endemism establishes a robust framework for evaluating patterns of species accumulation and evolutionary distinctiveness across China.

### 4.1 Phylogenetic Relationship of Asteraceae Plants in China

On one hand, due to the lack of molecular data, we excluded from the phylogenetic analysis those species that failed to form stable clustering relationships with their putative close relatives (Li et al. 2019; Zhu et al. 2021). On the other hand, some rare species and newly published ones could not be included in the analysis due to missing data. This may result in insufficient national coverage of species sampling in the phylogenetic tree, which in turn could affect the subsequent calculation of PD (phylogenetic diversity) and PE (phylogenetic endemism)—metrics that are highly dependent on the accuracy of branch lengths and phylogenetic relationships between groups. However, this study has covered nearly 73% of the generic taxa of Asteraceae in China, and the excluded species are mostly rare taxa with scattered distributions. Their absence has not altered the macropattern of overall diversity distribution, so the results still hold certain representativeness and reliability.

The phylogenetic tree indicates that most traditionally recognized subfamilies and tribes are monophyletic, with their monophyly strongly supported (Figure 2) (Funk et al. 2009; Fu et al. 2016). Within Mutisioideae, the monophyly of *Gerbera* is strongly supported, and it is sister to the disjunct genus *Leibnitzia* (BS = 100), consistent with previous hypotheses (Baird et al. 2010). Meanwhile, the placement of *Adenocaulon* within Mutisieae aligns with the findings of Kim et al. (2002), Fu et al. (2016), and Zhang, Huang, et al. (2021). Wunderlichioideae exhibits limited distribution records in China, with the present study focusing exclusively on two species (*Leucomeris decora* Kurz and *Nouelia insignis* Franch.) (Fu et al. 2016). Notably, the Chinese genera *Nouelia* and *Leucomeris* formed a well‐supported clade (BS = 100), consistent with recent research (Fu et al. 2016). This contradicts earlier classifications that place *Nouelia* and *Leucomeris* within Mutisieae (Gao et al. 2011; Shi et al. 2011; Fu et al. 2016). In Pertyoideae, the monotypic genus *Myripnois* was nested within *Pertya*, reflecting their morphological similarities (Gao et al. 2011; Shi et al. 2011; Fu et al. 2016). Carduoideae is one of the largest subfamilies in Asteraceae, comprising approximately 2850 species, while Gymnarrhenoideae was represented solely by *Gymnarrhena* and *Cavea*, the latter endemic to Himalayan alpine regions (Fu et al. 2016). Cichorioideae encompassed seven tribes, with Vernonieae and Cichorieae being predominant in China (Fu et al. 2016). Finally, Asteroideae, the largest subfamily in Asteraceae, included 15 tribes (accounting for ~68% of the total number of tribes) and exhibits high diversity (Fu et al. 2016). These results are largely consistent with previous studies at the classification level. However, uncertainties remain in poorly studied lineages (e.g., Wunderlichioideae and Pertyoideae), which require further support from additional molecular data and morphological evidence. Future studies should expand sampling to refine generic delimitations and test biogeographic hypotheses.

It is noteworthy that the challenge of resolving deep phylogenetic relationships or recent rapid radiative events represents a common issue in the field of phylogenetics, not limited to the plant kingdom. For instance, Zhang, Jin, et al. (2021); Zhang, Naicker, et al. (2024), in their study on the pathogenic fungus *Hypomyces* spp. (the causative agent of wet bubble disease in mushrooms), faced analogous difficulties in deciphering the evolutionary relationships among closely related pathogenic strains using multilocus datasets. Although the study organisms (higher plants vs. microorganisms) are markedly different, both cases must methodologically contend with the confounding effects of incomplete lineage sorting and rapid speciation on phylogenetic signal. This cross‐kingdom commonality underscores that the value of the phylogenetic framework we constructed for problematic Asteraceae genera extends beyond clarifying specific kinship relationships; it also provides a representative case study for understanding broader evolutionary biological questions. Future research, drawing on genomic approaches potentially applicable from fungal studies like those of Zhang, Naicker, et al. (2024), such as transcriptome sequencing, holds promise for offering breakthrough insights into resolving rapid radiations within the Asteraceae family.

### 4.2 Spatial Phylogenetic Analysis of Asteraceae in China

Correlation analysis and principal component analysis (PCA) jointly reveal that there is significant information redundancy among these four indices (including species richness, phylogenetic diversity, phylogenetic endemism, and weighted endemism). The high correlation (with a correlation coefficient *r* > 0.85) clearly indicates that they collectively depict the spatial pattern of biodiversity. Among them, species richness (SR), with a contribution rate of over 80%, fully demonstrates its dominant position among these indices. However, PD, PE, and WE are not redundant. They provide indispensable complementary information for the overall analysis. Specifically, SR and PD focus more on reflecting species abundance and the breadth of phylogenetic development, while PE and WE highlight the evolutionary uniqueness of region‐specific taxa. Therefore, despite the information redundancy among these four indices, each of them carries unique research value in this study. The specific analyses are as follows.

The high species richness of Asteraceae in southern and southwestern China (e.g., Yungui Plateau, Hengduan Mts, and Sichuan Basin) aligns with these regions being globally recognized biodiversity hotspots (Myers et al. 2000; López‐Pujol et al. 2011). Two key factors may contribute to this phenomenon. First, the complex topography of these areas—such as Hengduan Mts and Sichuan Basin, with their distinct altitudinal gradients (where mountains, canyons, and basins interweave)—creates diverse microhabitats, thereby promoting ecological specialization and allopatric speciation in Asteraceae (Xing and Ree 2017). Second, the relative climatic stability of southern China during Pleistocene glaciations served as a refuge for relict plant lineages (Qiu et al. 2011). The Qinling Mts, a microrefugia since the Pleistocene (Tang et al. 2017) and the boundary between warm temperate and temperate flora (Zhang et al. 2016), creates distinct climatic differences between the north and south; that is, the north is colder and drier, while the south is more humid and warmer. This likely contributes to the high Asteraceae species richness south of the Qinling Mts.

Our findings align with the current trend in macroecology towards integrating multisource data for spatial analysis. The studies by Das et al. (2022) and Antwi et al. (2024), which integrated space‐based parameters with field inventory data to assess forest biomass and compare soil fertility and management strategies in forest ecosystems across different continents, respectively, exemplify this approach. In contrast to their focus on ecosystem functional attributes, our work provides a nationwide assessment of evolutionary history as a unique dimension of biodiversity. This complements traditional functional assessments by addressing their potential oversight of unique evolutionary heritage. A region may exhibit high ecosystem functionality (e.g., biomass production) while harboring species that are evolutionarily redundant; whereas metrics such as PD and PE can identify areas like the Hengduan Mts that harbor a unique and irreplaceable evolutionary heritage.

Low phylogenetic diversity (PD) often results from habitat filtering (Webb et al. 2002; Mishler et al. 2014; Kraft et al. 2015), a hypothesis backed by numerous empirical studies (Thornhill et al. 2016, 2017; Scherson et al. 2017). Closely related taxa often exhibit evolutionarily conserved habitat preferences due to shared ancestry; conversely, recently diverged lineages tend to remain geographically restricted to their origin areas due to limited dispersal time. Given the arid habitats of western China, our study suggests that drought‐driven environmental filtering is the primary driver of low PD in Xinjiang, Qinghai, Gansu, Tibet, and western Inner Mongolia (Kraft et al. 2015). In contrast, higher PD values may arise from species being widely distributed across the phylogenetic tree, accumulating longer independent evolutionary branches that potentially retain more unique genetic and functional traits—thus holding great conservation value (Faith 1992; Mazel et al. 2018). Notably, species‐rich areas (e.g., the Hengduan Mts) also retain greater evolutionary history, indicating a potential correlation between PD and species richness (Pollock et al. 2017).

Northwest China (Altai Mts, Tianshan Mts, and Junggar Basin), South China (Hengduan Mts), and Taiwan Island exhibit high weighted endemism (WE) and phylogenetic endemism (PE) values (Figures 4, 6). High WE values indicate that these areas have numerous narrow‐range endemic species, potentially representing speciation or isolation hotspots, or featuring unique habitat conditions (e.g., islands, mountains) that support the long‐term preservation of endemic species (Stein et al. 2014). High PE values suggest the presence of evolutionarily distinct species or lineages with restricted distributions, implying these regions may preserve ancient relict taxa or have experienced prolonged independent evolution (Mishler et al. 2014). As one of the largest angiosperm families globally, Asteraceae frequently undergoes recent rapid radiations in isolated environments (e.g., islands, mountains), generating numerous endemic species (Baldwin and Wagner 2010). Molecular phylogenetics reveals that *Anaphalis morrisonicola* Hayata belongs to an early‐diverging clade within *Anaphalis*, showing substantial genetic divergence from mainland congeners (Nie et al. 2016). In studies of plant taxa, numerous independent glacial refugia have been identified in the Tianshan–Altai region, including *Gymnocarpos przewalskii* Maxim. (Ma et al. 2012) and *Reaumuria songarica* (Pall.) Maxim. (Li et al. 2012). Lower altitude valleys or peripheral mountainous areas were more likely to provide temporary shelters for plants to survive extreme cold (Meng et al. 2015). The intensification of aridification in northwestern China during the Pleistocene acted as an evolutionary driver, further promoting plant differentiation in the region (Meng and Zhang 2013; Xie and Zhang 2013; Jiang et al. 2014). These scattered findings on refugia across various taxa collectively support our conclusion that Tianshan–Altai Mts may be a center of phylogenetic endemism. Additionally, the Junggar Basin, surrounded by the Tianshan Mts and Altai Mts, forms a natural geographic barrier limiting species dispersal, which may contribute to the formation of endemic species. The Hengduan Mts, characterized by a series of north–south oriented deep valleys and high peaks (Ding et al. 2020), create complex habitats that promote localized adaptation and population divergence (Xing and Ree 2017), thereby fostering numerous narrowly endemic species (e.g., *Parasyncalathium* J. W. Zhang, Boufford & H. Sun, endemic to Himalayan–Hengduan Mts) (Zhang et al. 2011).

Underlying these macroevolutionary patterns are profound changes in molecular regulation that occur as species adapt to their environments. Recent research on epitranscriptomic mechanisms such as m^6^A RNA modification has revealed the central role of gene expression regulation in plant development and stress responses (Shan et al. 2025). We hypothesize that in hotspots of high PE and WE, such as the Hengduan Mts, the formation and adaptive evolution of endemic Asteraceae species may be closely associated with natural variation in the posttranscriptional regulation of their key developmental genes.

### 4.3 Conservation Priority Areas

Based on the spatial phylogenetic analysis, we found that three regions exhibit high values across all four indicators and thus warrant high conservation priority: the Tianshan–Altai Mts in northwestern China; most areas in southwestern China (particularly the Hengduan Mts); and Taiwan Island, which is geographically isolated from the mainland. Results from relative endemism analyses further indicate that these regions not only have high species richness but also have numerous endemic species and ancient evolutionary lineages restricted to these areas, retaining significant evolutionary potential (López‐Pujol et al. 2011; Kraft et al. 2015; Meng et al. 2015; Pollock et al. 2017; Xing and Ree 2017).

In addition to natural factors, human‐induced land‐use changes and habitat fragmentation may also have profoundly impacted the diversity patterns of Asteraceae in China (Xu et al. 2017). In the species‐rich southern and southwestern regions, rapid urbanization (e.g., urban sprawl in the Sichuan Basin) may fragment continuous habitats (Chen et al. 2023). Such fragmentation disrupts gene flow among Asteraceae populations and poses significant threats to narrow‐endemic species dependent on intact microhabitat gradients (López‐Pujol et al. 2011; Xu et al. 2017; Chen et al. 2023). In the arid northwest, overgrazing (e.g., on the Qinghai–Tibet Plateau) may lead to the degradation of grassland ecosystems, further exacerbating the homogenization of plant communities that already have low phylogenetic diversity (Wu et al. 2021). Similarly, in Taiwan, urban and tourism development in coastal and mountainous areas has severely reduced habitats of narrow‐endemic species such as *A. morrisonicola* (Nie et al. 2016), increasing their extinction risk. Collectively, these anthropogenic disturbances, combined with natural geographic barriers, not only impede species dispersal but may also accelerate the extinction of endemic lineages, thereby threatening the long‐term evolutionary potential of Asteraceae.

Therefore, when formulating conservation strategies, we recommend adopting an integrated approach. First, conservation decision‐making should directly incorporate spatial phylogenetic data to ensure priority protection of areas harboring irreplaceable evolutionary history. Second, building upon methodologies exemplified by Das et al. (2022) and Antwi et al. (2024), our layers of SR, PD, PE, and WE can be integrated with data on land‐use change and climate change scenarios to predict which evolutionary hotspots will face the most severe threats in the future. Finally, recognizing the fundamental role of molecular mechanisms in species adaptation, as elaborated by Shan et al. (2025), long‐term conservation biology should begin to focus on maintaining both genetic and epigenetic diversity, which is crucial for the long‐term persistence of species in rapidly changing environments.

## 5 Conclusions

Based on the reconstruction of the phylogenetic tree of Chinese Asteraceae, this study systematically analyzed the spatial distribution characteristics and conservation priorities of Asteraceae plants in China by integrating four indicators: species richness, phylogenetic diversity, weighted endemism, and phylogenetic endemism. The results suggest that the spatial distribution pattern of Chinese Asteraceae may be shaped by the combined effects of natural factors (e.g., topography and climate) and certain anthropogenic factors. Specifically, the Tianshan–Altai Mts in northwestern China, the southwest region (especially the Hengduan Mts), and Taiwan Island—geographically isolated from the mainland—harbor high ecological diversity due to their complex terrain and the relative stability of the Pleistocene climate, making them unique repositories of the phylogenetic history of Asteraceae. Thus, this study recommends prioritizing these areas. Moreover, research on the spatial phylogenetic patterns of Chinese Asteraceae remains in its early stages. Future studies should build upon the existing phylogenetic framework by integrating ecological modeling approaches widely used in macroecology, as well as multiomics methodologies such as transcriptomics and metabolomics. Such interdisciplinary integration will facilitate a deeper understanding of molecular adaptation mechanisms and support the development of more effective conservation strategies. This research direction is essential for addressing ongoing rapid environmental changes and for preserving both the current biodiversity and long‐term evolutionary potential of Asteraceae in China.

## Author Contributions

**Xinyi Zheng:** conceptualization (equal), writing – original draft (equal). **Xinyu Chen:** formal analysis (equal), software (equal). **Tianmeng Qu:** data curation (equal), formal analysis (equal). **Yanru Zhang:** formal analysis (equal). **Yizhen Shao:** data curation (equal). **Bing Zhang:** data curation (equal). **Zhixi Fu:** conceptualization (equal), writing – review and editing (equal). **Xiaoxia Zhang:** conceptualization (equal), writing – review and editing (equal).

## Conflicts of Interest

The authors declare no conflicts of interest.

## Supporting information


**Figure S1:** Phylogenetic tree of Asteraceae in China. 7 subfamilies and 22 tribes are shown.

**Table S1:** Voucher information and GenBank accession numbers for species sampled in this study.

## Data Availability

All data analyzed in this study are included within the article and attached to the Additional files.
