# Supplementary material for: Spatial Phylogenetics Reveals Endemism Hotspots and Conservation Priorities in Chinese Asteraceae
Source: Ecol Evol. 2025 Nov 2;15(11):e72403. doi: 10.1002/ece3.72403 (PMC12579969; doi:10.1002/ece3.72403)
Supplement: Supplementary file 2 — Table S1: Voucher information and GenBank accession numbers for species sampled in this study. [file ECE3-15-e72403-s002.docx]

**Table S1.** Voucher information and GenBank accession numbers for species sampled in this study.

| **Family** | **Subfamily** | **Tribe** | **Genus** | **Species** | **Voucher no** | **ITS** | ***mat*K** | ***ndh*F** | ***psb*A-*trn*H** | ***rbc*L** | ***rps*16** | ***trn*G** |
| --- | --- | --- | --- | --- | --- | --- | --- | --- | --- | --- | --- | --- |
| Asteraceae | Asteroideae | Millieae | *Acanthospermum* | *Acanthospermum hispidum* | NCBI | FJ696965.1 | FJ789804.1 | - | MK260836.1 | JQ933202.1 | - | - |
| Asteraceae | Asteroideae | Anthemideae | *Achillea* | *Achillea acuminata* | NCBI | EF577284.1 | MH660044.1 | EU334449.1 | JN224575.1 | MH658575.1 | - | - |
| Asteraceae | Asteroideae | Anthemideae | *Achillea* | *Achillea alpina* | NCBI | KX670802.1 | LC628094.1 | - | LC628114.1 | LC627470.1 | - | - |
| Asteraceae | Asteroideae | Anthemideae | *Achillea* | *Achillea asiatica* | NCBI | AY603209.1 | - | - | HQ451027.1 | - | EU129177.1 | - |
| Asteraceae | Asteroideae | Anthemideae | *Achillea* | *Achillea impatiens* | NCBI | AY603246.1 | - | - | - | - | - | - |
| Asteraceae | Asteroideae | Anthemideae | *Achillea* | *Achillea nobilis* | NCBI | AY603212.1 | - | - | - | - | - | - |
| Asteraceae | Asteroideae | Anthemideae | *Achillea* | *Achillea salicifolia* | NCBI | AY603251.1 | - | - | - | - | - | - |
| Asteraceae | Asteroideae | Anthemideae | *Achillea* | *Achillea setacea* | NCBI | AY603197.1 | - | - | HQ451070.1 | - | - | - |
| Asteraceae | Asteroideae | Anthemideae | *Achillea* | *Achillea wilsoniana* | NCBI | FJ980357.1 | HM989802.1 | EU334450.1 | JN224650.1 | MN192491.1 | - | - |
| Asteraceae | Asteroideae | Heliantheae | *Acmella* | *Acmella brachyglossa* | NCBI | KC981071.1 | - | - | - | - | - | - |
| Asteraceae | Asteroideae | Heliantheae | *Acmella* | *Acmella oleracea* | NCBI | MH765691.1 | MF350268.1 | - | MF348657.1 | MF349519.1 | - | - |
| Asteraceae | Asteroideae | Heliantheae | *Acmella* | *Acmella paniculata* | NCBI | KM887377.1 | HM989756.1 | - | OM677462.1 | OM127877.1 | - | - |
| Asteraceae | Mutisioideae | Mutisieae | *Adenocaulon* | *Adenocaulon himalaicum* | NCBI | MH711420.1 | MG710482.1 | L39401.1 | MG710469.1 | JQ933206.1 | - | - |
| Asteraceae | Asteroideae | Eupatorieae | *Adenostemma* | *Adenostemma lavenia* | NCBI | MH808069.1 | MK435673.1 | KX526881.1 | LC628949.1 | JQ933208.1 | - | - |
| Asteraceae | Asteroideae | Eupatorieae | *Ageratina* | *Ageratina adenophora* | NCBI | KY968839.1 | MT214803.1 | - | HQ221567.1 | HM849743.1 | - | - |
| Asteraceae | Asteroideae | Eupatorieae | *Ageratina* | *Ageratina riparia* | NCBI | AY576865.1 | - | - | - | MW818160.1 | - | - |
| Asteraceae | Asteroideae | Eupatorieae | *Ageratum* | *Ageratum conyzoides* | NCBI | MN889430.1 | MF159424.1 | KP454890.1 | MF143679.1 | JQ933210.1 | - | - |
| Asteraceae | Asteroideae | Eupatorieae | *Ageratum* | *Ageratum houstonianum* | NCBI | MH050129.1 | EU337054.1 | EU337042.1 | EU337031.1 | MH049903.1 | - | - |
| Asteraceae | Pertyoideae | Pertyeae | *Ainsliaea* | *Ainsliaea apiculata* | NCBI | LC605676.1 | EU385321.1 | EU385130.1 | LC605684.1 | EU384944.1 | - | - |
| Asteraceae | Pertyoideae | Pertyeae | *Ainsliaea* | *Ainsliaea aptera* | NCBI | AB288431.1 | MF786958.1 | - | MF785964.1 | MF786717.1 | - | - |
| Asteraceae | Pertyoideae | Pertyeae | *Ainsliaea* | *Ainsliaea apteroides* | NCBI | AB288432.1 | - | AB288514.1 | - | - | - | - |
| Asteraceae | Pertyoideae | Pertyeae | *Ainsliaea* | *Ainsliaea elegans* | NCBI | AB288435.1 | - | AB288517.1 | - | - | - | - |
| Asteraceae | Pertyoideae | Pertyeae | *Ainsliaea* | *Ainsliaea foliosa* | NCBI | AB288437.1 | - | - | - | - | - | - |
| Asteraceae | Pertyoideae | Pertyeae | *Ainsliaea* | *Ainsliaea fragrans* | NCBI | AB288439.1 | - | AB288519.1 | - | - | - | - |
| Asteraceae | Pertyoideae | Pertyeae | *Ainsliaea* | *Ainsliaea fulvipes* | NCBI | AB288440.1 | - | AB288521.1 | - | - | - | - |
| Asteraceae | Pertyoideae | Pertyeae | *Ainsliaea* | *Ainsliaea glabra* | NCBI | AB288441.1 | KF989914.1 | AB288522.1 | KF990024.1 | - | - | - |
| Asteraceae | Pertyoideae | Pertyeae | *Ainsliaea* | *Ainsliaea gracilis* | NCBI | AB288443.1 | - | AB535142.1 | - | - | - | - |
| Asteraceae | Pertyoideae | Pertyeae | *Ainsliaea* | *Ainsliaea grossedentata* | NCBI | AB288444.1 | - | AB288525.1 | - | - | - | - |
| Asteraceae | Pertyoideae | Pertyeae | *Ainsliaea* | *Ainsliaea henryi* | NCBI | AB288450.1 | - | AB288531.1 | - | - | - | - |
| Asteraceae | Pertyoideae | Pertyeae | *Ainsliaea* | *Ainsliaea latifolia* | NCBI | MH808074.1 | MK435676.1 | AB288528.1 | - | MN192494.1 | - | - |
| Asteraceae | Pertyoideae | Pertyeae | *Ainsliaea* | *Ainsliaea macrocephala* | NCBI | AB288452.1 | EU385322.1 | EU385131.1 | - | EU384945.1 | - | - |
| Asteraceae | Pertyoideae | Pertyeae | *Ainsliaea* | *Ainsliaea macroclinidioides* | NCBI | AB288456.1 | - | AB288534.1 | - | - | - | - |
| Asteraceae | Pertyoideae | Pertyeae | *Ainsliaea* | *Ainsliaea pertyoides* | NCBI | AB288458.1 | - | AB288538.1 | - | - | - | - |
| Asteraceae | Pertyoideae | Pertyeae | *Ainsliaea* | *Ainsliaea spicata* | NCBI | AB288459.1 | - | AB288539.1 | - | - | - | - |
| Asteraceae | Pertyoideae | Pertyeae | *Ainsliaea* | *Ainsliaea trinervis* | NCBI | AB288461.1 | - | AB288541.1 | - | - | - | - |
| Asteraceae | Pertyoideae | Pertyeae | *Ainsliaea* | *Ainsliaea walkeri* | NCBI | AB288464.1 | - | - | - | - | - | - |
| Asteraceae | Pertyoideae | Pertyeae | *Ainsliaea* | *Ainsliaea yunnanensis* | NCBI | AB288462.1 | - | AB288542.1 | - | - | - | - |
| Asteraceae | Asteroideae | Anthemideae | *Ajania* | *Ajania breviloba* | NCBI | EF577271.1 | - | - | KY312266.1 | - | - | - |
| Asteraceae | Asteroideae | Anthemideae | *Ajania* | *Ajania fastigiata* | NCBI | EF577272.1 | - | EU334451.1 | KY312267.1 | - | - | - |
| Asteraceae | Asteroideae | Anthemideae | *Ajania* | *Ajania fruticulosa* | NCBI | EF577273.1 | KX526529.1 | EU334452.1 | - | KX527160.1 | - | - |
| Asteraceae | Asteroideae | Anthemideae | *Ajania* | *Ajania khartensis* | NCBI | EF577274.1 | - | EU334453.1 | KY312268.1 | - | - | - |
| Asteraceae | Asteroideae | Anthemideae | *Ajania* | *Ajania myriantha* | NCBI | EF577275.1 | - | EU334454.1 | KY312269.1 | - | - | - |
| Asteraceae | Asteroideae | Anthemideae | *Ajania* | *Ajania nematoloba* | NCBI | EF577276.1 | - | EU334455.1 | KY312270.1 | - | - | - |
| Asteraceae | Asteroideae | Anthemideae | *Ajania* | *Ajania pallasiana* | NCBI | KX352152.1 | - | - | JN862002.1 | - | - | - |
| Asteraceae | Asteroideae | Anthemideae | *Ajania* | *Ajania przewalskii* | NCBI | EF577279.1 | - | EU334458.1 | KY312277.1 | - | - | - |
| Asteraceae | Asteroideae | Anthemideae | *Ajania* | *Ajania remotipinna* | NCBI | EF577280.1 | - | - | KY312280.1 | - | - | - |
| Asteraceae | Asteroideae | Anthemideae | *Ajania* | *Ajania salicifolia* | NCBI | EF577281.1 | MN273573.1 | EU334461.1 | KY312291.1 | MN192495.1 | - | - |
| Asteraceae | Asteroideae | Anthemideae | *Ajania* | *Ajania variifolia* | NCBI | EF577283.1 | - | EU334463.1 | KY312292.1 | - | - | - |
| Asteraceae | Carduoideae | Cardueae | *Alfredia* | *Alfredia acantholepis* | NCBI | AY826224.1 | - | - | - | - | - | - |
| Asteraceae | Carduoideae | Cardueae | *Alfredia* | *Alfredia cernua* | NCBI | AY826225.1 | AY013519.1 | KC589912.1 | - | KC589785.1 | - | - |
| Asteraceae | Carduoideae | Cardueae | *Alfredia* | *Alfredia nivea* | NCBI | AY826226.1 | AY785087.1 | KC589913.1 | - | KC589786.1 | MK598532.1 | - |
| Asteraceae | Asteroideae | Anthemideae | *Allardia* | *Allardia nivea* | NCBI | MK911862.1 | - | - | - | - | - | - |
| Asteraceae | Asteroideae | Anthemideae | *Allardia* | *Allardia tomentosa* | NCBI | LC313953.1 | LC548600.1 | - | - | LC554191.1 | - | - |
| Asteraceae | Asteroideae | Anthemideae | *Allardia* | *Allardia tridactylites* | NCBI | MK911864.1 | KX526530.1 | KX526966.1 | - | KX527158.1 | - | - |
| Asteraceae | Carduoideae | Cardueae | *Amberboa* | *Amberboa turanica* | NCBI | MK226415.1 | - | JF754832.1 | - | - | - | - |
| Asteraceae | Asteroideae | Heliantheae | *Ambrosia* | *Ambrosia artemisiifolia* | NCBI | MH518067.1 | HQ593164.1 | - | MH464303.1 | MN812528.1 | - | - |
| Asteraceae | Asteroideae | Heliantheae | *Ambrosia* | *Ambrosia psilostachya* | NCBI | MH984890.1 | MK509405.1 | - | MH985561.1 | KJ773262.1 | - | - |
| Asteraceae | Asteroideae | Heliantheae | *Ambrosia* | *Ambrosia trifida* | NCBI | MH984892.1 | AY215762.1 | AF384693.2 | EF590670.1 | AY215082.1 | - | AY865263.1 |
| Asteraceae | Asteroideae | Gnaphalieae | *Anaphalis* | *Anaphalis aureopunctata* | NCBI | MH712729.1 | MH714290.1 | - | - | MH657490.1 | - | - |
| Asteraceae | Asteroideae | Gnaphalieae | *Anaphalis* | *Anaphalis bicolor* | NCBI | JQ895409.1 | - | - | - | - | - | - |
| Asteraceae | Asteroideae | Gnaphalieae | *Anaphalis* | *Anaphalis bulleyana* | NCBI | JQ895412.1 | - | - | - | - | - | - |
| Asteraceae | Asteroideae | Gnaphalieae | *Anaphalis* | *Anaphalis busua* | NCBI | KT865445.1 | - | - | - | - | - | - |
| Asteraceae | Asteroideae | Gnaphalieae | *Anaphalis* | *Anaphalis contorta* | NCBI | JQ895420.1 | - | - | - | - | - | - |
| Asteraceae | Asteroideae | Gnaphalieae | *Anaphalis* | *Anaphalis contortiformis* | NCBI | JQ895428.1 | - | - | - | - | - | - |
| Asteraceae | Asteroideae | Gnaphalieae | *Anaphalis* | *Anaphalis delavayi* | NCBI | JQ895430.1 | - | - | - | - | - | - |
| Asteraceae | Asteroideae | Gnaphalieae | *Anaphalis* | *Anaphalis elegans* | NCBI | JQ895431.1 | - | - | - | - | - | - |
| Asteraceae | Asteroideae | Gnaphalieae | *Anaphalis* | *Anaphalis flaccida* | NCBI | MH117418.1 | MH116522.1 | - | MH116970.1 | MH116038.1 | - | - |
| Asteraceae | Asteroideae | Gnaphalieae | *Anaphalis* | *Anaphalis flavescens* | NCBI | MH808077.1 | - | - | - | - | - | - |
| Asteraceae | Asteroideae | Gnaphalieae | *Anaphalis* | *Anaphalis gracilis* | NCBI | HG797719.1 | - | HG797829.1 | - | - | - | - |
| Asteraceae | Asteroideae | Gnaphalieae | *Anaphalis* | *Anaphalis hancockii* | NCBI | MH808079.1 | MN251232.1 | - | - | MN192497.1 | - | - |
| Asteraceae | Asteroideae | Gnaphalieae | *Anaphalis* | *Anaphalis hondae* | NCBI | JQ895444.1 | - | - | - | - | - | - |
| Asteraceae | Asteroideae | Gnaphalieae | *Anaphalis* | *Anaphalis latialata* | NCBI | JQ895445.1 | - | - | - | - | - | - |
| Asteraceae | Asteroideae | Gnaphalieae | *Anaphalis* | *Anaphalis likiangensis* | NCBI | MH117422.1 | MH116524.1 | - | MH116972.1 | MH116041.1 | - | - |
| Asteraceae | Asteroideae | Gnaphalieae | *Anaphalis* | *Anaphalis margaritacea* | NCBI | MH711554.1 | HM445632.1 | HM445660.1 | MH116975.1 | MH116044.1 | - | - |
| Asteraceae | Asteroideae | Gnaphalieae | *Anaphalis* | *Anaphalis morrisonicola* | NCBI | KT865451.1 | MT384655.1 | - | - | - | - | - |
| Asteraceae | Asteroideae | Gnaphalieae | *Anaphalis* | *Anaphalis muliensis* | NCBI | JQ895467.1 | - | - | - | - | - | - |
| Asteraceae | Asteroideae | Gnaphalieae | *Anaphalis* | *Anaphalis nepalensis* | NCBI | EF107657.1 | MH116528.1 | - | MH116977.1 | MN192498.1 | - | - |
| Asteraceae | Asteroideae | Gnaphalieae | *Anaphalis* | *Anaphalis oxyphylla* | NCBI | JQ895478.1 | - | - | - | - | - | - |
| Asteraceae | Asteroideae | Gnaphalieae | *Anaphalis* | *Anaphalis pachylaena* | NCBI | JQ895479.1 | - | - | - | - | - | - |
| Asteraceae | Asteroideae | Gnaphalieae | *Anaphalis* | *Anaphalis pannosa* | NCBI | JQ895480.1 | - | - | - | - | - | - |
| Asteraceae | Asteroideae | Gnaphalieae | *Anaphalis* | *Anaphalis plicata* | NCBI | JQ895482.1 | - | - | - | - | - | - |
| Asteraceae | Asteroideae | Gnaphalieae | *Anaphalis* | *Anaphalis porphyrolepis* | NCBI | JQ895483.1 | - | - | - | - | - | - |
| Asteraceae | Asteroideae | Gnaphalieae | *Anaphalis* | *Anaphalis sinica* | NCBI | MH711217.1 | MH659676.1 | - | - | MN204715.1 | - | - |
| Asteraceae | Asteroideae | Gnaphalieae | *Anaphalis* | *Anaphalis souliei* | NCBI | JQ895486.1 | - | - | - | - | - | - |
| Asteraceae | Asteroideae | Gnaphalieae | *Anaphalis* | *Anaphalis stenocephala* | NCBI | KT865453.1 | - | - | - | - | - | - |
| Asteraceae | Asteroideae | Gnaphalieae | *Anaphalis* | *Anaphalis surculosa* | NCBI | JQ895492.1 | - | - | - | - | - | - |
| Asteraceae | Asteroideae | Gnaphalieae | *Anaphalis* | *Anaphalis szechuanensis* | NCBI | KT865454.1 | - | - | - | - | - | - |
| Asteraceae | Asteroideae | Gnaphalieae | *Anaphalis* | *Anaphalis tibetica* | NCBI | JQ895494.1 | - | - | - | - | - | - |
| Asteraceae | Asteroideae | Gnaphalieae | *Anaphalis* | *Anaphalis triplinervis* | NCBI | U95291.1 | - | - | - | JQ933215.1 | - | - |
| Asteraceae | Asteroideae | Gnaphalieae | *Anaphalis* | *Anaphalis virens* | NCBI | JQ895500.1 | - | - | - | - | - | - |
| Asteraceae | Asteroideae | Gnaphalieae | *Anaphalis* | *Anaphalis virgata* | NCBI | JQ895501.1 | - | - | - | - | - | - |
| Asteraceae | Asteroideae | Gnaphalieae | *Anaphalis* | *Anaphalis viridis* | NCBI | JQ895502.1 | - | - | - | - | - | - |
| Asteraceae | Asteroideae | Gnaphalieae | *Anaphalis* | *Anaphalis xylorhiza* | NCBI | JQ895505.1 | - | - | - | - | - | - |
| Asteraceae | Asteroideae | Gnaphalieae | *Anaphalis* | *Anaphalis yunnanensis* | NCBI | JQ895506.1 | - | - | - | - | - | - |
| Asteraceae | Carduoideae | Cardueae | *Ancathia* | *Ancathia igniaria* | NCBI | AY914811.1 | KX526531.1 | KC589916.1 | - | KC589789.1 | - | - |
| Asteraceae | Asteroideae | Athroismeae | *Anisopappus* | *Anisopappus chinensis* | NCBI | KY210057.1 | - | KY210098.1 | KY209974.1 | - | MT886601.1 | - |
| Asteraceae | Asteroideae | Gnaphalieae | *Antennaria* | *Antennaria dioica* | NCBI | MN443041.1 | HM445620.1 | HM445686.1 | - | HE574602.1 | - | - |
| Asteraceae | Carduoideae | Cardueae | *Arctium* | *Arctium lappa* | NCBI | MH710842.1 | AY013520.1 | AB530944.1 | AB727572.1 | JQ933225.1 | MK598531.1 | - |
| Asteraceae | Carduoideae | Cardueae | *Arctium* | *Arctium tomentosum* | NCBI | MN918904.1 | KT249934.1 | - | - | MG221512.1 | - | - |
| Asteraceae | Asteroideae | Astereae | *Arctogeron* | *Arctogeron gramineum* | NCBI | JN315928.1 | - | - | - | - | - | - |
| Asteraceae | Asteroideae | Anthemideae | *Artemisia* | *Artemisia absinthium* | NCBI | KX581790.1 | MG946952.1 | EU334460.1 | MG947068.1 | MK348958.1 | - | JN043291.1 |
| Asteraceae | Asteroideae | Anthemideae | *Artemisia* | *Artemisia adamsii* | NCBI | AM398844.1 | - | - | - | - | - | - |
| Asteraceae | Asteroideae | Anthemideae | *Artemisia* | *Artemisia anethifolia* | NCBI | LK391723.1 | - | - | - | - | - | - |
| Asteraceae | Asteroideae | Anthemideae | *Artemisia* | *Artemisia annua* | NCBI | KX421779.1 | JQ173387.1 | - | MT758518.1 | KJ667633.1 | - | - |
| Asteraceae | Asteroideae | Anthemideae | *Artemisia* | *Artemisia anomala* | NCBI | MN947286.1 | - | - | JX073845.1 | - | - | - |
| Asteraceae | Asteroideae | Anthemideae | *Artemisia* | *Artemisia argyi* | NCBI | MH711480.1 | HM989726.1 | - | KU555809.1 | GQ436429.1 | - | - |
| Asteraceae | Asteroideae | Anthemideae | *Artemisia* | *Artemisia atrovirens* | NCBI | MH808086.1 | MK435678.1 | - | - | MN192500.1 | - | - |
| Asteraceae | Asteroideae | Anthemideae | *Artemisia* | *Artemisia austriaca* | NCBI | KX581798.1 | MK954255.1 | - | MT758506.1 | MK954364.1 | - | - |
| Asteraceae | Asteroideae | Anthemideae | *Artemisia* | *Artemisia bargusinensis* | NCBI | JF326533.1 | - | - | - | - | - | - |
| Asteraceae | Asteroideae | Anthemideae | *Artemisia* | *Artemisia blepharolepis* | NCBI | LK391725.1 | - | - | - | - | - | - |
| Asteraceae | Asteroideae | Anthemideae | *Artemisia* | *Artemisia caespitosa* | NCBI | AM398855.1 | - | - | - | - | - | - |
| Asteraceae | Asteroideae | Anthemideae | *Artemisia* | *Artemisia campestris* | NCBI | MH095575.1 | MK925653.1 | - | MT637780.1 | JX848403.1 | - | - |
| Asteraceae | Asteroideae | Anthemideae | *Artemisia* | *Artemisia capillaris* | NCBI | KX421746.1 | JQ173388.1 | - | FJ418750.1 | JQ173395.1 | - | - |
| Asteraceae | Asteroideae | Anthemideae | *Artemisia* | *Artemisia caruifolia* | NCBI | MH711600.1 | MH660001.1 | - | FJ418751.1 | MH658685.1 | - | - |
| Asteraceae | Asteroideae | Anthemideae | *Artemisia* | *Artemisia comaiensis* | NCBI | MH293168.1 | MH293242.1 | - | - | MH293309.1 | - | - |
| Asteraceae | Asteroideae | Anthemideae | *Artemisia* | *Artemisia demissa* | NCBI | JF326539.1 | - | - | - | - | - | - |
| Asteraceae | Asteroideae | Anthemideae | *Artemisia* | *Artemisia desertorum* | NCBI | KC493082.1 | - | - | - | - | - | - |
| Asteraceae | Asteroideae | Anthemideae | *Artemisia* | *Artemisia divaricata* | NCBI | KC493076.1 | KJ372399.1 | - | KJ372429.1 | KT280075.1 | KU865582.1 | - |
| Asteraceae | Asteroideae | Anthemideae | *Artemisia* | *Artemisia dracunculus* | NCBI | ON685399.1 | MN167189.1 | MK800823.1 | KX581958.1 | KX582009.1 | - | - |
| Asteraceae | Asteroideae | Anthemideae | *Artemisia* | *Artemisia dubia* | NCBI | KT965653.1 | - | - | - | - | - | - |
| Asteraceae | Asteroideae | Anthemideae | *Artemisia* | *Artemisia eriopoda* | NCBI | MH711469.1 | MH660003.1 | - | KU555752.1 | MH658580.1 | - | - |
| Asteraceae | Asteroideae | Anthemideae | *Artemisia* | *Artemisia freyniana* | NCBI | AM398868.1 | - | - | - | - | - | - |
| Asteraceae | Asteroideae | Anthemideae | *Artemisia* | *Artemisia gilvescens* | NCBI | LC324740.1 | - | - | - | - | LC323148.1 | - |
| Asteraceae | Asteroideae | Anthemideae | *Artemisia* | *Artemisia giraldii* | NCBI | JF326549.1 | - | - | - | - | - | - |
| Asteraceae | Asteroideae | Anthemideae | *Artemisia* | *Artemisia globosoides* | NCBI | JF326552.1 | - | - | - | - | - | - |
| Asteraceae | Asteroideae | Anthemideae | *Artemisia* | *Artemisia gmelinii* | NCBI | FJ980317.1 | KX581908.1 | - | KX581961.1 | GQ436432.1 | - | - |
| Asteraceae | Asteroideae | Anthemideae | *Artemisia* | *Artemisia igniaria* | NCBI | MH711500.1 | JQ173389.1 | - | JQ173376.1 | JQ173396.1 | - | - |
| Asteraceae | Asteroideae | Anthemideae | *Artemisia* | *Artemisia incisa* | NCBI | KC493079.1 | - | - | - | - | - | - |
| Asteraceae | Asteroideae | Anthemideae | *Artemisia* | *Artemisia indica* | NCBI | MH100676.1 | MH116553.1 | - | MH116997.1 | MH116071.1 | LC323147.1 | - |
| Asteraceae | Asteroideae | Anthemideae | *Artemisia* | *Artemisia integrifolia* | NCBI | AM398880.1 | - | - | JN862046.1 | - | - | - |
| Asteraceae | Asteroideae | Anthemideae | *Artemisia* | *Artemisia japonica* | NCBI | KP856178.1 | KF530805.1 | - | EF091612.1 | MN185069.1 | - | - |
| Asteraceae | Asteroideae | Anthemideae | *Artemisia* | *Artemisia keiskeana* | NCBI | KT965665.1 | - | - | - | - | - | - |
| Asteraceae | Asteroideae | Anthemideae | *Artemisia* | *Artemisia klementzae* | NCBI | JF326558.1 | - | - | - | - | - | - |
| Asteraceae | Asteroideae | Anthemideae | *Artemisia* | *Artemisia lactiflora* | NCBI | MH711655.1 | HM989729.1 | - | JQ173378.1 | GU724219.1 | - | - |
| Asteraceae | Asteroideae | Anthemideae | *Artemisia* | *Artemisia lagocephala* | NCBI | JX051673.1 | - | - | JN862051.1 | - | - | - |
| Asteraceae | Asteroideae | Anthemideae | *Artemisia* | *Artemisia lancea* | NCBI | MH711476.1 | MH660013.1 | - | - | MH658543.1 | - | - |
| Asteraceae | Asteroideae | Anthemideae | *Artemisia* | *Artemisia lavandulifolia* | NCBI | KX421742.1 | - | - | KU555823.1 | GQ436484.1 | - | - |
| Asteraceae | Asteroideae | Anthemideae | *Artemisia* | *Artemisia leucophylla* | NCBI | AM398890.1 | - | - | JN862055.1 | - | - | - |
| Asteraceae | Asteroideae | Anthemideae | *Artemisia* | *Artemisia macilenta* | NCBI | JF326563.1 | - | - | - | - | - | - |
| Asteraceae | Asteroideae | Anthemideae | *Artemisia* | *Artemisia macrantha* | NCBI | AM398892.1 | - | - | - | - | - | - |
| Asteraceae | Asteroideae | Anthemideae | *Artemisia* | *Artemisia macrocephala* | NCBI | KX581808.1 | KX581909.1 | - | KX581963.1 | KX582014.1 | - | - |
| Asteraceae | Asteroideae | Anthemideae | *Artemisia* | *Artemisia manshurica* | NCBI | JF326564.1 | - | - | - | - | - | - |
| Asteraceae | Asteroideae | Anthemideae | *Artemisia* | *Artemisia marschalliana* | NCBI | MT161424.1 | MK954223.1 | - | MT637763.1 | MK954326.1 | - | - |
| Asteraceae | Asteroideae | Anthemideae | *Artemisia* | *Artemisia mongolica* | NCBI | JX051683.1 | - | - | JX073853.1 | - | - | - |
| Asteraceae | Asteroideae | Anthemideae | *Artemisia* | *Artemisia montana* | NCBI | KT965654.1 | - | - | JX073848.1 | - | - | - |
| Asteraceae | Asteroideae | Anthemideae | *Artemisia* | *Artemisia nanschanica* | NCBI | JF326567.1 | - | - | - | - | - | - |
| Asteraceae | Asteroideae | Anthemideae | *Artemisia* | *Artemisia nilagirica* | NCBI | MK481441.1 | KF648716.1 | AF153632.1 | HE966492.1 | KM360653.1 | - | - |
| Asteraceae | Asteroideae | Anthemideae | *Artemisia* | *Artemisia obtusiloba* | NCBI | AM398900.1 | - | - | JN862065.1 | - | - | - |
| Asteraceae | Asteroideae | Anthemideae | *Artemisia* | *Artemisia ordosica* | NCBI | JF326568.1 | - | - | - | - | - | - |
| Asteraceae | Asteroideae | Anthemideae | *Artemisia* | *Artemisia palustris* | NCBI | JF326570.1 | - | - | - | - | - | - |
| Asteraceae | Asteroideae | Anthemideae | *Artemisia* | *Artemisia pewzowii* | NCBI | JF326572.1 | - | - | - | - | - | - |
| Asteraceae | Asteroideae | Anthemideae | *Artemisia* | *Artemisia phaeolepis* | NCBI | AM398905.1 | - | - | - | - | - | - |
| Asteraceae | Asteroideae | Anthemideae | *Artemisia* | *Artemisia pontica* | NCBI | KX581809.1 | KX581913.1 | - | KX581967.1 | KX582018.1 | - | - |
| Asteraceae | Asteroideae | Anthemideae | *Artemisia* | *Artemisia prattii* | NCBI | JF342550.1 | MF694828.1 | - | - | MF694951.1 | - | - |
| Asteraceae | Asteroideae | Anthemideae | *Artemisia* | *Artemisia princeps* | NCBI | KT965667.1 | - | - | KJ025046.1 | KM218339.1 | - | - |
| Asteraceae | Asteroideae | Anthemideae | *Artemisia* | *Artemisia pubescens* | NCBI | KX581813.1 | KX581916.1 | - | KX581970.1 | KX582021.1 | - | - |
| Asteraceae | Asteroideae | Anthemideae | *Artemisia* | *Artemisia rupestris* | NCBI | AJ297261.1 | MG224957.1 | - | JN862078.1 | KX582023.1 | - | - |
| Asteraceae | Asteroideae | Anthemideae | *Artemisia* | *Artemisia rutifolia* | NCBI | MH092832.1 | - | - | MH330161.1 | - | - | - |
| Asteraceae | Asteroideae | Anthemideae | *Artemisia* | *Artemisia saposhnikovii* | NCBI | JX051716.1 | - | - | JX073879.1 | - | - | - |
| Asteraceae | Asteroideae | Anthemideae | *Artemisia* | *Artemisia scoparia* | NCBI | MN505001.1 | HM989797.1 | - | MN505003.1 | GU724242.1 | - | - |
| Asteraceae | Asteroideae | Anthemideae | *Artemisia* | *Artemisia selengensis* | NCBI | MW306626.1 | - | - | MW306642.1 | - | - | - |
| Asteraceae | Asteroideae | Anthemideae | *Artemisia* | *Artemisia sericea* | NCBI | EF577290.1 | - | - | JN862086.1 | - | - | - |
| Asteraceae | Asteroideae | Anthemideae | *Artemisia* | *Artemisia sieversiana* | NCBI | KX581824.1 | JQ173391.1 | - | KX581981.1 | JQ173398.1 | - | - |
| Asteraceae | Asteroideae | Anthemideae | *Artemisia* | *Artemisia songarica* | NCBI | JF326577.1 | - | - | - | - | - | - |
| Asteraceae | Asteroideae | Anthemideae | *Artemisia* | *Artemisia stolonifera* | NCBI | JX051680.1 | LC618841.1 | - | KU555821.1 | - | - | - |
| Asteraceae | Asteroideae | Anthemideae | *Artemisia* | *Artemisia stracheyi* | NCBI | AM774421.1 | - | - | AB683350.1 | - | - | - |
| Asteraceae | Asteroideae | Anthemideae | *Artemisia* | *Artemisia stricta* | NCBI | KC493073.1 | - | - | - | - | - | - |
| Asteraceae | Asteroideae | Anthemideae | *Artemisia* | *Artemisia sylvatica* | NCBI | JX051685.1 | - | - | JX073854.1 | - | - | - |
| Asteraceae | Asteroideae | Anthemideae | *Artemisia* | *Artemisia tanacetifolia* | NCBI | AM398923.1 | - | - | - | - | - | - |
| Asteraceae | Asteroideae | Anthemideae | *Artemisia* | *Artemisia tangutica* | NCBI | KC493072.1 | - | - | KU555838.1 | - | - | - |
| Asteraceae | Asteroideae | Anthemideae | *Artemisia* | *Artemisia tournefortiana* | NCBI | KX581826.1 | KX581931.1 | - | KX581985.1 | KX582036.1 | - | - |
| Asteraceae | Asteroideae | Anthemideae | *Artemisia* | *Artemisia verbenacea* | NCBI | MK481441.1 | KF648716.1 | AF153632.1 | HE966492.1 | KM360653.1 | - | - |
| Asteraceae | Asteroideae | Anthemideae | *Artemisia* | *Artemisia vestita* | NCBI | KX421722.1 | - | - | KU555751.1 | - | - | - |
| Asteraceae | Asteroideae | Anthemideae | *Artemisia* | *Artemisia vulgaris* | NCBI | MK481441.1 | KF648716.1 | AF153632.1 | HE966492.1 | KM360653.1 | - | - |
| Asteraceae | Asteroideae | Anthemideae | *Artemisia* | *Artemisia waltonii* | NCBI | JF326582.1 | - | - | - | - | - | - |
| Asteraceae | Asteroideae | Anthemideae | *Artemisia* | *Artemisia xanthochroa* | NCBI | JF326584.1 | - | - | - | - | - | - |
| Asteraceae | Asteroideae | Anthemideae | *Artemisia* | *Artemisia xerophytica* | NCBI | AM398929.1 | - | - | - | - | - | - |
| Asteraceae | Cichorioideae | Cichorieae | *Askellia* | *Askellia flexuosa* | NCBI | EU363596.1 | KX526532.1 | KX526960.1 | - | KX527149.1 | KC968108.1 | - |
| Asteraceae | Cichorioideae | Cichorieae | *Askellia* | *Askellia pygmaea* | NCBI | MG217936.1 | - | MK800641.1 | - | MG222906.1 | - | - |
| Asteraceae | Asteroideae | Astereae | *Aster* | *Aster ageratoides* | NCBI | MH711534.1 | MH660083.1 | - | MH117003.1 | GQ436475.1 | - | - |
| Asteraceae | Asteroideae | Astereae | *Aster* | *Aster albescens* | NCBI | MH117454.1 | MK435682.1 | - | MH117001.1 | MH116075.1 | - | - |
| Asteraceae | Asteroideae | Astereae | *Aster* | *Aster alpinus* | NCBI | MH398798.1 | KP175137.1 | - | LC027423.1 | KX371936.1 | MH398901.1 | - |
| Asteraceae | Asteroideae | Astereae | *Aster* | *Aster altaicus* | NCBI | LC027396.1 | MF158683.1 | - | - | MF688977.1 | - | - |
| Asteraceae | Asteroideae | Astereae | *Aster* | *Aster argyropholis* | NCBI | JN543793.1 | - | - | - | - | - | - |
| Asteraceae | Asteroideae | Astereae | *Aster* | *Aster asteroides* | NCBI | JN543841.1 | - | - | - | - | - | - |
| Asteraceae | Asteroideae | Astereae | *Aster* | *Aster auriculatus* | NCBI | JN543754.1 | - | - | - | - | - | - |
| Asteraceae | Asteroideae | Astereae | *Aster* | *Aster baccharoides* | NCBI | JN543805.1 | - | - | - | - | - | - |
| Asteraceae | Asteroideae | Astereae | *Aster* | *Aster batangensis* | NCBI | JN543859.1 | - | - | - | - | - | - |
| Asteraceae | Asteroideae | Astereae | *Aster* | *Aster brachytrichus* | NCBI | JN543838.1 | - | - | - | - | - | - |
| Asteraceae | Asteroideae | Astereae | *Aster* | *Aster brevis* | NCBI | MH808097.1 | KX526535.1 | KX526923.1 | - | KX527153.1 | - | - |
| Asteraceae | Asteroideae | Astereae | *Aster* | *Aster crenatifolius* | NCBI | JN543712.1 | - | - | - | - | - | - |
| Asteraceae | Asteroideae | Astereae | *Aster* | *Aster diplostephioides* | NCBI | JN543847.1 | KT280183.1 | - | - | KT280076.1 | - | - |
| Asteraceae | Asteroideae | Astereae | *Aster* | *Aster dolichophyllus* | NCBI | MH807107.1 | - | - | - | - | - | - |
| Asteraceae | Asteroideae | Astereae | *Aster* | *Aster dolichopodus* | NCBI | JN543775.1 | - | - | - | - | - | - |
| Asteraceae | Asteroideae | Astereae | *Aster* | *Aster falcifolius* | NCBI | JN543802.1 | - | - | - | - | - | - |
| Asteraceae | Asteroideae | Astereae | *Aster* | *Aster fanjingshanicus* | NCBI | JN543829.1 | - | - | - | - | - | - |
| Asteraceae | Asteroideae | Astereae | *Aster* | *Aster farreri* | NCBI | MH808095.1 | MK435683.1 | - | - | - | - | - |
| Asteraceae | Asteroideae | Astereae | *Aster* | *Aster fuscescens* | NCBI | JN543751.1 | - | - | - | - | - | - |
| Asteraceae | Asteroideae | Astereae | *Aster* | *Aster handelii* | NCBI | JN543820.1 | - | - | - | - | - | - |
| Asteraceae | Asteroideae | Astereae | *Aster* | *Aster hersileoides* | NCBI | JN543787.1 | - | - | - | - | - | - |
| Asteraceae | Asteroideae | Astereae | *Aster* | *Aster heterolepis* | NCBI | JN543823.1 | - | - | - | - | - | - |
| Asteraceae | Asteroideae | Astereae | *Aster* | *Aster hispidus* | NCBI | HQ154044.1 | MH659694.1 | - | - | MH658213.1 | - | - |
| Asteraceae | Asteroideae | Astereae | *Aster* | *Aster homochlamydeus* | NCBI | JN543784.1 | - | - | - | - | - | - |
| Asteraceae | Asteroideae | Astereae | *Aster* | *Aster incisus* | NCBI | JN543721.1 | - | - | - | - | - | - |
| Asteraceae | Asteroideae | Astereae | *Aster* | *Aster indicus* | NCBI | EF108396.1 | - | - | GU724256.1 | GU724231.1 | - | - |
| Asteraceae | Asteroideae | Astereae | *Aster* | *Aster jishouensis* | NCBI | JN543808.1 | - | - | - | - | - | - |
| Asteraceae | Asteroideae | Astereae | *Aster* | *Aster lautureanus* | NCBI | MT731680.1 | - | - | - | - | - | - |
| Asteraceae | Asteroideae | Astereae | *Aster* | *Aster lavandulifolius* | NCBI | JN543796.1 | - | - | - | - | - | - |
| Asteraceae | Asteroideae | Astereae | *Aster* | *Aster longipetiolatus* | NCBI | JN315936.1 | - | - | - | - | - | - |
| Asteraceae | Asteroideae | Astereae | *Aster* | *Aster maackii* | NCBI | LC482292.1 | - | - | LC623610.1 | LC623598.1 | - | - |
| Asteraceae | Asteroideae | Astereae | *Aster* | *Aster mangshanensis* | NCBI | JN543760.1 | - | - | - | - | - | - |
| Asteraceae | Asteroideae | Astereae | *Aster* | *Aster megalanthus* | NCBI | MK693188.1 | - | - | - | - | - | - |
| Asteraceae | Asteroideae | Astereae | *Aster* | *Aster meyendorffii* | NCBI | KJ587604.1 | - | - | - | KM218354.1 | - | - |
| Asteraceae | Asteroideae | Astereae | *Aster* | *Aster mongolicus* | NCBI | MH711404.1 | MH659906.1 | - | - | MH658430.1 | - | - |
| Asteraceae | Asteroideae | Astereae | *Aster* | *Aster moupinensis* | NCBI | MH807113.1 | - | - | - | - | - | - |
| Asteraceae | Asteroideae | Astereae | *Aster* | *Aster nitidus* | NCBI | JN543790.1 | - | - | - | - | - | - |
| Asteraceae | Asteroideae | Astereae | *Aster* | *Aster oreophilus* | NCBI | JN543826.1 | - | - | - | - | - | - |
| Asteraceae | Asteroideae | Astereae | *Aster* | *Aster panduratus* | NCBI | JN543757.1 | - | - | - | - | - | - |
| Asteraceae | Asteroideae | Astereae | *Aster* | *Aster pekinensis* | NCBI | MK128489.1 | - | - | - | - | - | - |
| Asteraceae | Asteroideae | Astereae | *Aster* | *Aster piccolii* | NCBI | MH710712.1 | MH659031.1 | - | - | MH657460.1 | - | - |
| Asteraceae | Asteroideae | Astereae | *Aster* | *Aster poliothamnus* | NCBI | JN543763.1 | MK435685.1 | - | - | - | - | - |
| Asteraceae | Asteroideae | Astereae | *Aster* | *Aster procerus* | NCBI | KP313683.1 | - | - | - | - | - | - |
| Asteraceae | Asteroideae | Astereae | *Aster* | *Aster pseudosimplex* | NCBI | JN543733.1 | - | - | - | - | - | - |
| Asteraceae | Asteroideae | Astereae | *Aster* | *Aster pycnophyllus* | NCBI | JN543799.1 | - | - | - | - | - | - |
| Asteraceae | Asteroideae | Astereae | *Aster* | *Aster salwinensis* | NCBI | KP313689.1 | - | - | - | - | - | - |
| Asteraceae | Asteroideae | Astereae | *Aster* | *Aster sampsonii* | NCBI | JN543811.1 | - | - | - | - | - | - |
| Asteraceae | Asteroideae | Astereae | *Aster* | *Aster scaber* | NCBI | HQ154049.1 | AB262046.1 | - | LC484279.1 | - | - | - |
| Asteraceae | Asteroideae | Astereae | *Aster* | *Aster senecioides* | NCBI | JN543856.1 | - | - | - | - | - | - |
| Asteraceae | Asteroideae | Astereae | *Aster* | *Aster setchuenensis* | NCBI | ON527427.1 | - | - | - | - | - | - |
| Asteraceae | Asteroideae | Astereae | *Aster* | *Aster shimadae* | NCBI | MT731683.1 | - | - | - | - | - | - |
| Asteraceae | Asteroideae | Astereae | *Aster* | *Aster sikuensis* | NCBI | JN543766.1 | - | - | - | - | - | - |
| Asteraceae | Asteroideae | Astereae | *Aster* | *Aster sinoangustifolius* | NCBI | KP313684.1 | - | - | - | - | - | - |
| Asteraceae | Asteroideae | Astereae | *Aster* | *Aster smithianus* | NCBI | MH710810.1 | MH659154.1 | - | - | MH657585.1 | - | - |
| Asteraceae | Asteroideae | Astereae | *Aster* | *Aster souliei* | NCBI | JN543835.1 | KT280184.1 | - | - | KT280077.1 | - | - |
| Asteraceae | Asteroideae | Astereae | *Aster* | *Aster taiwanensis* | NCBI | LC482291.1 | MT384659.1 | - | LC484271.1 | - | - | - |
| Asteraceae | Asteroideae | Astereae | *Aster* | *Aster taliangshanensis* | NCBI | JN543772.1 | - | - | - | - | - | - |
| Asteraceae | Asteroideae | Astereae | *Aster* | *Aster tataricus* | NCBI | MH711581.1 | HM989804.1 | - | GQ435142.1 | GQ436492.1 | - | - |
| Asteraceae | Asteroideae | Astereae | *Aster* | *Aster tianmenshanensis* | NCBI | KP313679.1 | - | - | - | - | - | - |
| Asteraceae | Asteroideae | Astereae | *Aster* | *Aster tongolensis* | NCBI | GU444006.1 | MF786966.1 | - | MF785965.1 | MF786732.1 | - | - |
| Asteraceae | Asteroideae | Astereae | *Aster* | *Aster turbinatus* | NCBI | JN543814.1 | - | - | - | - | - | - |
| Asteraceae | Asteroideae | Astereae | *Aster* | *Aster verticillatus* | NCBI | JN543706.1 | - | - | - | JQ933465.1 | - | - |
| Asteraceae | Asteroideae | Astereae | *Aster* | *Aster vestitus* | NCBI | JN543769.1 | - | - | - | - | - | - |
| Asteraceae | Asteroideae | Astereae | *Aster* | *Aster yunnanensis* | NCBI | JN543853.1 | - | - | - | - | - | - |
| Asteraceae | Asteroideae | Astereae | *Asterothamnus* | *Asterothamnus centraliasiaticus* | NCBI | JN315930.1 | KX526534.1 | - | - | KX527385.1 | - | - |
| Asteraceae | Asteroideae | Astereae | *Asterothamnus* | *Asterothamnus fruticosus* | NCBI | JN315929.1 | - | - | - | - | - | - |
| Asteraceae | Asteroideae | Astereae | *Asterothamnus* | *Asterothamnus poliifolius* | NCBI | KJ711904.1 | - | - | - | - | - | - |
| Asteraceae | Carduoideae | Cardueae | *Atractylodes* | *Atractylodes koreana* | NCBI | KC416904.1 | AB008760.1 | - | EU571452.1 | MG237907.1 | - | - |
| Asteraceae | Carduoideae | Cardueae | *Atractylodes* | *Atractylodes lancea* | NCBI | MH710865.1 | AB008762.1 | - | GQ435071.1 | MG237909.1 | - | - |
| Asteraceae | Carduoideae | Cardueae | *Atractylodes* | *Atractylodes macrocephala* | NCBI | MH808098.1 | MG237897.1 | - | KX346974.2 | MG237910.1 | - | - |
| Asteraceae | Asteroideae | Eupatorieae | *Austroeupatorium* | *Austroeupatorium inulifolium* | NCBI | AB032052.1 | KJ637186.1 | KP454899.1 | - | MG833448.1 | - | - |
| Asteraceae | Asteroideae | Coreopsideae | *Bidens* | *Bidens bipinnata* | NCBI | MK545692.1 | MK435688.1 | - | MK546068.1 | KJ773309.1 | - | - |
| Asteraceae | Asteroideae | Coreopsideae | *Bidens* | *Bidens biternata* | NCBI | MK545693.1 | MK435690.1 | - | MK546069.1 | MK903548.1 | - | - |
| Asteraceae | Asteroideae | Coreopsideae | *Bidens* | *Bidens cernua* | NCBI | MK545697.1 | JN893870.1 | - | MK546073.1 | HQ589973.1 | - | - |
| Asteraceae | Asteroideae | Coreopsideae | *Bidens* | *Bidens frondosa* | NCBI | MK545714.1 | MF159456.1 | - | KP643559.1 | MF135475.1 | - | - |
| Asteraceae | Asteroideae | Coreopsideae | *Bidens* | *Bidens parviflora* | NCBI | KF731840.1 | MK435692.1 | - | KF731857.1 | MN204724.1 | - | - |
| Asteraceae | Asteroideae | Coreopsideae | *Bidens* | *Bidens pilosa* | NCBI | MH711608.1 | MT214842.1 | - | MF143646.1 | MH767488.1 | - | - |
| Asteraceae | Asteroideae | Coreopsideae | *Bidens* | *Bidens tripartita* | NCBI | MH711585.1 | AY551475.1 | - | KF731864.1 | KM360671.1 | - | - |
| Asteraceae | Asteroideae | Heliantheae | *Blainvillea* | *Blainvillea acmella* | NCBI | MH152213.1 | - | - | MH152126.1 | JQ933237.1 | - | - |
| Asteraceae | Asteroideae | Inuleae | *Blumea* | *Blumea aromatica* | NCBI | KP052657.1 | - | - | EU195586.1 | - | - | - |
| Asteraceae | Asteroideae | Inuleae | *Blumea* | *Blumea balsamifera* | NCBI | KF443294.1 | KF443303.1 | LN607601.1 | EF211006.1 | KF443314.1 | - | - |
| Asteraceae | Asteroideae | Inuleae | *Blumea* | *Blumea clarkei* | NCBI | EU195648.1 | - | FM208908.1 | EU195587.1 | - | - | - |
| Asteraceae | Asteroideae | Inuleae | *Blumea* | *Blumea fistulosa* | NCBI | KP052661.1 | - | - | EU195589.1 | - | - | - |
| Asteraceae | Asteroideae | Inuleae | *Blumea* | *Blumea flava* | NCBI | EF210960.1 | - | LN607607.1 | EU195590.1 | JQ933238.1 | - | - |
| Asteraceae | Asteroideae | Inuleae | *Blumea* | *Blumea formosana* | NCBI | KP052665.1 | MT384653.1 | - | - | - | - | - |
| Asteraceae | Asteroideae | Inuleae | *Blumea* | *Blumea hieraciifolia* | NCBI | KP052662.1 | - | FM208911.1 | EF210985.1 | - | - | - |
| Asteraceae | Asteroideae | Inuleae | *Blumea* | *Blumea hookeri* | NCBI | EF210934.1 | - | FM208909.1 | EU195588.1 | - | - | - |
| Asteraceae | Asteroideae | Inuleae | *Blumea* | *Blumea lacera* | NCBI | LN607422.1 | - | LN607603.1 | EF210987.1 | - | - | - |
| Asteraceae | Asteroideae | Inuleae | *Blumea* | *Blumea lanceolaria* | NCBI | KP052664.1 | - | FM208912.1 | EF210988.1 | - | - | - |
| Asteraceae | Asteroideae | Inuleae | *Blumea* | *Blumea martiniana* | NCBI | EU195657.1 | - | - | EU195592.1 | - | - | - |
| Asteraceae | Asteroideae | Inuleae | *Blumea* | *Blumea megacephala* | NCBI | KP052670.1 | - | - | EF211010.1 | - | - | - |
| Asteraceae | Asteroideae | Inuleae | *Blumea* | *Blumea mollis* | NCBI | KP052666.1 | - | - | EF210991.1 | - | - | - |
| Asteraceae | Asteroideae | Inuleae | *Blumea* | *Blumea napifolia* | NCBI | EU195664.1 | - | FM208913.1 | EF211007.1 | - | - | - |
| Asteraceae | Asteroideae | Inuleae | *Blumea* | *Blumea oxyodonta* | NCBI | EU195665.1 | - | - | EF211004.1 | - | - | - |
| Asteraceae | Asteroideae | Inuleae | *Blumea* | *Blumea riparia* | NCBI | KP052668.1 | KX526537.1 | LN607605.1 | EU195600.1 | KX527129.1 | - | - |
| Asteraceae | Asteroideae | Inuleae | *Blumea* | *Blumea saussureoides* | NCBI | KP052669.1 | - | - | - | - | - | - |
| Asteraceae | Asteroideae | Inuleae | *Blumea* | *Blumea sessiliflora* | NCBI | EF210947.1 | - | - | EF210995.1 | - | - | - |
| Asteraceae | Asteroideae | Inuleae | *Blumea* | *Blumea virens* | NCBI | EU195673.1 | - | - | EU195602.1 | - | - | - |
| Asteraceae | Asteroideae | Anthemideae | *Brachanthemum* | *Brachanthemum pulvinatum* | NCBI | EF577291.1 | - | EU334464.1 | JF940250.1 | - | - | - |
| Asteraceae | Asteroideae | Calenduleae | *Calendula* | *Calendula officinalis* | NCBI | ON685403.1 | AF151446.1 | L39439.1 | MF419685.1 | JQ933250.1 | - | AY865251.1 |
| Asteraceae | Asteroideae | Astereae | *Callistephus* | *Callistephus chinensis* | NCBI | JN315931.1 | KP175070.1 | AB530920.1 | - | AB530954.1 | - | - |
| Asteraceae | Asteroideae | Heliantheae | *Calyptocarpus* | *Calyptocarpus vialis* | NCBI | MT335834.1 | AY215770.1 | AF384698.3 | OK469555.1 | AY215090.1 | - | - |
| Asteraceae | Asteroideae | Anthemideae | *Cancrinia* | *Cancrinia chrysocephala* | NCBI | LC313958.1 | - | KX526962.1 | - | KX527401.1 | - | - |
| Asteraceae | Carduoideae | Cardueae | *Carduus* | *Carduus acanthoides* | NCBI | JX867641.1 | KT249939.1 | - | - | MN601439.1 | - | - |
| Asteraceae | Carduoideae | Cardueae | *Carduus* | *Carduus crispus* | NCBI | MH711464.1 | JN894376.1 | - | AY914835.1 | MH658527.1 | - | - |
| Asteraceae | Carduoideae | Cardueae | *Carduus* | *Carduus nutans* | NCBI | KY242485.1 | KT176586.1 | KT176826.1 | - | KT178106.1 | KT179020.1 | - |
| Asteraceae | Carduoideae | Cardueae | *Carlina* | *Carlina biebersteinii* | NCBI | MK238394.1 | - | - | EU571429.1 | - | - | - |
| Asteraceae | Asteroideae | Inuleae | *Carpesium* | *Carpesium cernuum* | NCBI | KY696283.1 | MH659176.1 | FM208917.1 | - | MH657607.1 | - | - |
| Asteraceae | Asteroideae | Inuleae | *Carpesium* | *Carpesium cordatum* | NCBI | MW116504.1 | - | - | MW137581.1 | - | - | - |
| Asteraceae | Asteroideae | Inuleae | *Carpesium* | *Carpesium divaricatum* | NCBI | MW116511.1 | - | LN607612.1 | EF211013.1 | - | - | - |
| Asteraceae | Asteroideae | Inuleae | *Carpesium* | *Carpesium faberi* | NCBI | MH674408.1 | - | - | MH674418.1 | - | - | - |
| Asteraceae | Asteroideae | Inuleae | *Carpesium* | *Carpesium longifolium* | NCBI | MH674409.1 | - | - | MH674419.1 | - | - | - |
| Asteraceae | Asteroideae | Inuleae | *Carpesium* | *Carpesium macrocephalum* | NCBI | FM995368.1 | MH659790.1 | FM208918.1 | FM998660.1 | MH658311.1 | - | - |
| Asteraceae | Asteroideae | Inuleae | *Carpesium* | *Carpesium minus* | NCBI | MW116506.1 | - | - | - | - | - | - |
| Asteraceae | Asteroideae | Inuleae | *Carpesium* | *Carpesium scapiforme* | NCBI | MH674415.1 | KX526541.1 | KX526875.1 | MH674425.1 | KX527011.1 | - | - |
| Asteraceae | Asteroideae | Inuleae | *Carpesium* | *Carpesium szechuanense* | NCBI | MH674416.1 | KX526542.1 | KX526915.1 | - | KX527002.1 | - | - |
| Asteraceae | Asteroideae | Inuleae | *Carpesium* | *Carpesium tracheliifolium* | NCBI | KY696283.1 | MH659176.1 | FM208917.1 | - | MH657607.1 | - | - |
| Asteraceae | Asteroideae | Inuleae | *Carpesium* | *Carpesium triste* | NCBI | MH117477.1 | MH116577.1 | - | MH117025.1 | MH116101.1 | - | - |
| Asteraceae | Asteroideae | Inuleae | *Carpesium* | *Carpesium velutinum* | NCBI | MH674411.1 | - | - | MH674421.1 | - | - | - |
| Asteraceae | Carduoideae | Cardueae | *Carthamus* | *Carthamus tinctorius* | NCBI | KY397481.1 | EU385331.1 | EU385139.1 | KX108703.1 | EU384953.1 | GU990463.1 | - |
| Asteraceae | Gymnarrhenoideae | Gymnarrheneae | *Cavea* | *Cavea tanguensis* | NCBI | - | - | JQ922540.1 | - | - | - | - |
| Asteraceae | Carduoideae | Cardueae | *Centaurea* | *Centaurea diffusa* | NCBI | MW383496.1 | KC969483.1 | - | - | MG224346.1 | AY316613.1 | - |
| Asteraceae | Carduoideae | Cardueae | *Centaurea* | *Centaurea iberica* | NCBI | KY676857.1 | KY676852.1 | - | - | JQ933258.1 | - | - |
| Asteraceae | Carduoideae | Cardueae | *Centaurea* | *Centaurea scabiosa* | NCBI | MW383492.1 | JN894134.1 | - | FJ459726.1 | KJ746251.1 | - | - |
| Asteraceae | Carduoideae | Cardueae | *Centaurea* | *Centaurea virgata* | NCBI | DQ319174.1 | KY676851.1 | - | - | - | - | - |
| Asteraceae | Asteroideae | Athroismeae | *Centipeda* | *Centipeda minima* | NCBI | KY210084.1 | JX564842.1 | HE862360.1 | GQ435087.1 | MH203365.1 | - | - |
| Asteraceae | Cichorioideae | Vernonieae | *Centratherum* | *Centratherum punctatum* | NCBI | EF155754.1 | JN837403.1 | EU385141.1 | - | EU384955.1 | - | - |
| Asteraceae | Asteroideae | Eupatorieae | *Chromolaena* | *Chromolaena odorata* | NCBI | MN889428.1 | MN558588.1 | KP454921.1 | MH837933.1 | MH767491.1 | - | - |
| Asteraceae | Asteroideae | Anthemideae | *Chrysanthemum* | *Chrysanthemum arcticum* | NCBI | EF577286.1 | JN966105.1 | EU334448.1 | EF091604.1 | MG221983.1 | - | AB234652.1 |
| Asteraceae | Asteroideae | Anthemideae | *Chrysanthemum* | *Chrysanthemum chanetii* | NCBI | EF577295.1 | - | - | EF091605.1 | - | - | - |
| Asteraceae | Asteroideae | Anthemideae | *Chrysanthemum* | *Chrysanthemum dichroum* | NCBI | KX352148.1 | - | EU334471.1 | EF091607.1 | - | - | - |
| Asteraceae | Asteroideae | Anthemideae | *Chrysanthemum* | *Chrysanthemum indicum* | NCBI | MH808111.1 | - | EU334475.1 | - | MN185071.1 | - | AB234647.1 |
| Asteraceae | Asteroideae | Anthemideae | *Chrysanthemum* | *Chrysanthemum lavandulifolium* | NCBI | MH710799.1 | HM989791.1 | EU334474.1 | EF091614.1 | GQ436485.1 | - | - |
| Asteraceae | Asteroideae | Anthemideae | *Chrysanthemum* | *Chrysanthemum mongolicum* | NCBI | AF314593.1 | - | - | JF940266.1 | - | - | - |
| Asteraceae | Asteroideae | Anthemideae | *Chrysanthemum* | *Chrysanthemum morifolium* | NCBI | KC215400.1 | - | - | MK353561.1 | - | - | AY865254.1 |
| Asteraceae | Asteroideae | Anthemideae | *Chrysanthemum* | *Chrysanthemum naktongense* | NCBI | KJ183121.1 | MH714228.1 | - | JF940270.1 | MH714041.1 | - | - |
| Asteraceae | Asteroideae | Anthemideae | *Chrysanthemum* | *Chrysanthemum oreastrum* | NCBI | EF577304.1 | - | EU334472.1 | JF940288.1 | - | - | - |
| Asteraceae | Asteroideae | Anthemideae | *Chrysanthemum* | *Chrysanthemum potentilloides* | NCBI | KC694535.1 | - | - | KC695271.1 | - | - | - |
| Asteraceae | Asteroideae | Anthemideae | *Chrysanthemum* | *Chrysanthemum rhombifolium* | NCBI | KC694542.1 | - | - | KC695278.1 | - | - | - |
| Asteraceae | Asteroideae | Anthemideae | *Chrysanthemum* | *Chrysanthemum vestitum* | NCBI | KX352149.1 | - | EU334478.1 | EF091619.1 | - | - | - |
| Asteraceae | Cichorioideae | Cichorieae | *Cicerbita* | *Cicerbita auriculiformis* | NCBI | OP070154.1 | - | - | LT722090.1 | - | - | - |
| Asteraceae | Cichorioideae | Cichorieae | *Cicerbita* | *Cicerbita azurea* | NCBI | OP070153.1 | - | - | KF485806.1 | - | - | - |
| Asteraceae | Cichorioideae | Cichorieae | *Cicerbita* | *Cicerbita roborowskii* | NCBI | LT722009.1 | KX526544.1 | - | HQ436159.1 | KX527006.1 | - | - |
| Asteraceae | Cichorioideae | Cichorieae | *Cichorium* | *Cichorium intybus* | NCBI | ON685428.1 | JN895731.1 | L39390.1 | FJ493262.1 | MN192512.1 | GQ983984.1 | - |
| Asteraceae | Carduoideae | Cardueae | *Cirsium* | *Cirsium arvense* | NCBI | MH711113.1 | HQ593238.1 | - | FJ395528.1 | MN601440.1 | - | - |
| Asteraceae | Carduoideae | Cardueae | *Cirsium* | *Cirsium botryodes* | NCBI | MH710807.1 | MH659150.1 | - | - | MH657581.1 | - | - |
| Asteraceae | Carduoideae | Cardueae | *Cirsium* | *Cirsium eriophoroides* | NCBI | MH293131.1 | MH293203.1 | - | - | - | - | - |
| Asteraceae | Carduoideae | Cardueae | *Cirsium* | *Cirsium handelii* | NCBI | MT914292.1 | MT928687.1 | - | - | MT921019.1 | - | - |
| Asteraceae | Carduoideae | Cardueae | *Cirsium* | *Cirsium henryi* | NCBI | AF443697.1 | - | - | - | - | - | - |
| Asteraceae | Carduoideae | Cardueae | *Cirsium* | *Cirsium interpositum* | NCBI | MN335066.1 | - | - | - | - | - | - |
| Asteraceae | Carduoideae | Cardueae | *Cirsium* | *Cirsium japonicum* | NCBI | KM051436.1 | HM989744.1 | - | LC365329.1 | GQ436443.1 | - | - |
| Asteraceae | Carduoideae | Cardueae | *Cirsium* | *Cirsium leo* | NCBI | MH808117.1 | MK435700.1 | - | - | MN163305.1 | - | - |
| Asteraceae | Carduoideae | Cardueae | *Cirsium* | *Cirsium lidjiangense* | NCBI | AY914828.1 | - | - | AY914836.1 | - | - | - |
| Asteraceae | Carduoideae | Cardueae | *Cirsium* | *Cirsium lineare* | NCBI | AF443727.1 | KX526545.1 | KX526977.1 | - | - | - | - |
| Asteraceae | Carduoideae | Cardueae | *Cirsium* | *Cirsium monocephalum* | NCBI | MH711731.1 | MH658940.1 | - | - | MH657366.1 | - | - |
| Asteraceae | Carduoideae | Cardueae | *Cirsium* | *Cirsium pendulum* | NCBI | JX274260.1 | - | - | - | - | - | - |
| Asteraceae | Carduoideae | Cardueae | *Cirsium* | *Cirsium shansiense* | NCBI | MN335097.1 | - | - | - | - | - | - |
| Asteraceae | Carduoideae | Cardueae | *Cirsium* | *Cirsium vulgare* | NCBI | JX867638.1 | MT214892.1 | MN275364.1 | MN275426.1 | MN601441.1 | - | - |
| Asteraceae | Asteroideae | Eupatorieae | *Conoclinium* | *Conoclinium coelestinum* | NCBI | MN556262.1 | EU337056.1 | EU337044.1 | MF348603.1 | DQ006064.1 | - | - |
| Asteraceae | Asteroideae | Astereae | *Conyza* | *Conyza bonariensis* | NCBI | LC574986.1 | MT214833.1 | - | MK261017.1 | KJ773397.1 | MH559530.1 | - |
| Asteraceae | Asteroideae | Astereae | *Conyza* | *Conyza canadensis* | NCBI | MH734253.1 | MT214835.1 | - | MH464294.1 | MH767498.1 | MH559528.1 | - |
| Asteraceae | Asteroideae | Astereae | *Conyza* | *Conyza japonica* | NCBI | JN315938.1 | KX526551.1 | - | - | KX527398.1 | - | - |
| Asteraceae | Asteroideae | Astereae | *Conyza* | *Conyza stricta* | NCBI | GU045827.1 | KR734807.1 | - | KR735966.1 | KR737032.1 | - | - |
| Asteraceae | Asteroideae | Coreopsideae | *Coreopsis* | *Coreopsis grandiflora* | NCBI | KY968944.1 | AY551493.1 | - | KR817857.1 | MF135451.1 | KR824946.1 | - |
| Asteraceae | Asteroideae | Coreopsideae | *Coreopsis* | *Coreopsis lanceolata* | NCBI | KY968882.1 | AY551495.1 | - | MK546188.1 | MF135450.1 | KR824948.1 | - |
| Asteraceae | Asteroideae | Coreopsideae | *Coreopsis* | *Coreopsis tinctoria* | NCBI | GU724273.1 | AY551490.1 | L39461.1 | KR817875.1 | JX848408.1 | - | AY865262.1 |
| Asteraceae | Asteroideae | Coreopsideae | *Cosmos* | *Cosmos bipinnatus* | NCBI | FJ980345.1 | EU049361.1 | L39462.1 | MK546193.1 | KM218349.1 | - | - |
| Asteraceae | Asteroideae | Coreopsideae | *Cosmos* | *Cosmos sulphureus* | NCBI | MZ411546.1 | EU049362.1 | - | - | - | - | - |
| Asteraceae | Asteroideae | Anthemideae | *Cotula* | *Cotula australis* | NCBI | LC088733.1 | MT214861.1 | - | LC088736.1 | KT626684.1 | - | - |
| Asteraceae | Carduoideae | Cardueae | *Cousinia* | *Cousinia polycephala* | NCBI | AY826273.1 | AY373668.1 | KC589958.1 | - | KC589837.1 | - | - |
| Asteraceae | Asteroideae | Senecioneae | *Crassocephalum* | *Crassocephalum crepidioides* | NCBI | KY968855.1 | MF159418.1 | EF537958.2 | AY155640.1 | GU817753.1 | KU750236.1 | - |
| Asteraceae | Asteroideae | Senecioneae | *Cremanthodium* | *Cremanthodium brunneopilosum* | NCBI | AY176131.1 | - | - | - | - | - | KY970562.1 |
| Asteraceae | Asteroideae | Senecioneae | *Cremanthodium* | *Cremanthodium bulbilliferum* | NCBI | MH293169.1 | MH293243.1 | - | - | MH293310.1 | - | KY970563.1 |
| Asteraceae | Asteroideae | Senecioneae | *Cremanthodium* | *Cremanthodium campanulatum* | NCBI | MH293136.1 | MH293211.1 | - | - | MH293279.1 | KU750237.1 | KY970521.1 |
| Asteraceae | Asteroideae | Senecioneae | *Cremanthodium* | *Cremanthodium daochengense* | NCBI | MH293137.1 | MH293212.1 | - | - | MH293280.1 | - | - |
| Asteraceae | Asteroideae | Senecioneae | *Cremanthodium* | *Cremanthodium decaisnei* | NCBI | AY723269.1 | - | AY723232.1 | - | - | - | KY970565.1 |
| Asteraceae | Asteroideae | Senecioneae | *Cremanthodium* | *Cremanthodium discoideum* | NCBI | AY723271.1 | - | AY723235.1 | - | - | - | KY970566.1 |
| Asteraceae | Asteroideae | Senecioneae | *Cremanthodium* | *Cremanthodium ellisii* | NCBI | AY723272.1 | - | AY723236.1 | - | - | - | KY970567.1 |
| Asteraceae | Asteroideae | Senecioneae | *Cremanthodium* | *Cremanthodium helianthus* | NCBI | KY979037.1 | - | - | - | - | - | KY970396.1 |
| Asteraceae | Asteroideae | Senecioneae | *Cremanthodium* | *Cremanthodium humile* | NCBI | EF538175.1 | EF537915.1 | AY723234.1 | EF538061.1 | - | - | KY970524.1 |
| Asteraceae | Asteroideae | Senecioneae | *Cremanthodium* | *Cremanthodium lineare* | NCBI | AY176134.1 | KT280194.1 | AY723237.1 | - | KT280088.1 | - | KY970530.1 |
| Asteraceae | Asteroideae | Senecioneae | *Cremanthodium* | *Cremanthodium microglossum* | NCBI | AY176135.1 | - | AY723238.1 | - | - | - | - |
| Asteraceae | Asteroideae | Senecioneae | *Cremanthodium* | *Cremanthodium nobile* | NCBI | KY979059.1 | - | - | - | - | - | KY970425.1 |
| Asteraceae | Asteroideae | Senecioneae | *Cremanthodium* | *Cremanthodium principis* | NCBI | KY979060.1 | - | - | - | - | KU750238.1 | KY970426.1 |
| Asteraceae | Asteroideae | Senecioneae | *Cremanthodium* | *Cremanthodium rhodocephalum* | NCBI | KY979099.1 | - | - | - | - | KU750239.1 | KY970522.1 |
| Asteraceae | Asteroideae | Senecioneae | *Cremanthodium* | *Cremanthodium stenoglossum* | NCBI | AY176136.1 | - | AY723233.1 | MF688696.1 | - | MF688710.1 | KY970574.1 |
| Asteraceae | Cichorioideae | Cichorieae | *Crepidiastrum* | *Crepidiastrum chelidoniifolium* | NCBI | LC656558.1 | LC656555.1 | - | LC656565.1 | LC656548.1 | - | - |
| Asteraceae | Cichorioideae | Cichorieae | *Crepidiastrum* | *Crepidiastrum denticulatum* | NCBI | MW938645.1 | AJ633139.1 | - | KF196116.1 | KF196028.1 | - | - |
| Asteraceae | Cichorioideae | Cichorieae | *Crepidiastrum* | *Crepidiastrum diversifolium* | NCBI | KC968073.1 | - | - | - | KC968017.1 | KC968150.1 | - |
| Asteraceae | Cichorioideae | Cichorieae | *Crepidiastrum* | *Crepidiastrum humifusum* | NCBI | KC968034.1 | - | - | - | KC968020.1 | KC968115.1 | - |
| Asteraceae | Cichorioideae | Cichorieae | *Crepidiastrum* | *Crepidiastrum lanceolatum* | NCBI | MW938642.1 | MF064310.1 | - | MF064865.1 | LC693418.1 | AB598601.1 | - |
| Asteraceae | Cichorioideae | Cichorieae | *Crepidiastrum* | *Crepidiastrum sonchifolium* | NCBI | MH808121.1 | KT250094.1 | - | - | MN192515.1 | - | - |
| Asteraceae | Cichorioideae | Cichorieae | *Crepidiastrum* | *Crepidiastrum taiwanianum* | NCBI | AB002616.1 | KX526546.1 | - | - | KX527376.1 | - | - |
| Asteraceae | Cichorioideae | Cichorieae | *Crepidiastrum* | *Crepidiastrum tenuifolium* | NCBI | LT721999.1 | LR743493.1 | - | LT722058.1 | - | - | - |
| Asteraceae | Cichorioideae | Cichorieae | *Crepis* | *Crepis chrysantha* | NCBI | EU363622.1 | EU363560.1 | - | - | - | - | - |
| Asteraceae | Cichorioideae | Cichorieae | *Crepis* | *Crepis crocea* | NCBI | EU363590.1 | - | - | - | - | - | - |
| Asteraceae | Cichorioideae | Cichorieae | *Crepis* | *Crepis darvazica* | NCBI | EU363600.1 | EU363558.1 | - | - | - | - | - |
| Asteraceae | Cichorioideae | Cichorieae | *Crepis* | *Crepis elongata* | NCBI | KC968071.1 | MN764302.1 | - | - | JQ933285.1 | KC968132.1 | - |
| Asteraceae | Cichorioideae | Cichorieae | *Crepis* | *Crepis lyrata* | NCBI | MT234673.1 | - | - | - | - | MT234701.1 | - |
| Asteraceae | Cichorioideae | Cichorieae | *Crepis* | *Crepis multicaulis* | NCBI | KF485539.1 | LR743494.1 | - | KF485794.1 | - | - | - |
| Asteraceae | Cichorioideae | Cichorieae | *Crepis* | *Crepis oreades* | NCBI | EU363640.1 | EU363572.1 | - | - | - | - | - |
| Asteraceae | Cichorioideae | Cichorieae | *Crepis* | *Crepis sibirica* | NCBI | AJ633358.1 | - | - | - | - | MT234723.1 | - |
| Asteraceae | Cichorioideae | Cichorieae | *Crepis* | *Crepis tectorum* | NCBI | EU363643.1 | EU363536.1 | AF218348.1 | - | JX848409.1 | JN177472.1 | - |
| Asteraceae | Asteroideae | Astereae | *Crinitina* | *Crinitina villosa* | NCBI | MT703647.1 | - | - | MT682324.1 | - | - | - |
| Asteraceae | Asteroideae | Anthemideae | *Crossostephium* | *Crossostephium chinensis* | NCBI | EF577293.1 | MF064315.1 | EU334467.1 | MF064867.1 | KX527375.1 | - | - |
| Asteraceae | Carduoideae | Cardueae | *Crupina* | *Crupina vulgaris* | NCBI | KC768345.1 | KC969524.1 | JF754842.1 | - | - | - | - |
| Asteraceae | Carduoideae | Cardueae | *Cyanus* | *Cyanus segetum* | NCBI | MZ191013.1 | KC969482.1 | AB530921.1 | KJ652211.1 | AB530955.1 | - | - |
| Asteraceae | Asteroideae | Inuleae | *Cyathocline* | *Cyathocline purpurea* | NCBI | KF684943.1 | - | - | - | JQ933291.1 | - | - |
| Asteraceae | Asteroideae | Astereae | *Dichrocephala* | *Dichrocephala auriculata* | NCBI | JN315919.1 | KP175090.1 | - | - | - | - | - |
| Asteraceae | Asteroideae | Astereae | *Dichrocephala* | *Dichrocephala benthamii* | NCBI | FJ980350.1 | HM989788.1 | - | GQ435127.1 | JQ933302.1 | - | - |
| Asteraceae | Asteroideae | Astereae | *Dichrocephala* | *Dichrocephala integrifolia* | NCBI | LC027384.1 | KP175082.1 | - | - | KP110336.1 | - | - |
| Asteraceae | Carduoideae | Cardueae | *Dolomiaea* | *Dolomiaea berardioidea* | NCBI | EU239684.1 | - | - | AY914837.1 | - | - | - |
| Asteraceae | Carduoideae | Cardueae | *Dolomiaea* | *Dolomiaea calophylla* | NCBI | AY914816.1 | - | - | AY914838.1 | - | - | - |
| Asteraceae | Carduoideae | Cardueae | *Dolomiaea* | *Dolomiaea edulis* | NCBI | AY914817.1 | - | - | AY914839.1 | - | - | - |
| Asteraceae | Carduoideae | Cardueae | *Dolomiaea* | *Dolomiaea georgei* | NCBI | MT914280.1 | MT928695.1 | - | - | MT921020.1 | - | - |
| Asteraceae | Asteroideae | Doroniceae | *Doronicum* | *Doronicum altaicum* | NCBI | MG748356.1 | MK435704.1 | - | MF785888.1 | MN185073.1 | - | - |
| Asteraceae | Asteroideae | Doroniceae | *Doronicum* | *Doronicum calotum* | NCBI | MH293142.1 | MH293217.1 | - | - | - | - | - |
| Asteraceae | Asteroideae | Doroniceae | *Doronicum* | *Doronicum oblongifolium* | NCBI | MG748394.1 | - | - | - | - | - | - |
| Asteraceae | Asteroideae | Doroniceae | *Doronicum* | *Doronicum stenoglossum* | NCBI | KU696021.1 | - | AY723253.1 | - | - | KU750240.1 | - |
| Asteraceae | Cichorioideae | Cichorieae | *Dubyaea* | *Dubyaea amoena* | NCBI | MK043351.1 | - | - | MK046024.1 | - | MK046054.1 | - |
| Asteraceae | Cichorioideae | Cichorieae | *Dubyaea* | *Dubyaea atropurpurea* | NCBI | MK382839.1 | KF195996.1 | - | KF196128.1 | KF196040.1 | MK382859.1 | - |
| Asteraceae | Cichorioideae | Cichorieae | *Dubyaea* | *Dubyaea blinii* | NCBI | MK043355.1 | KF195989.1 | - | KF196121.1 | KF196033.1 | MK046058.1 | - |
| Asteraceae | Cichorioideae | Cichorieae | *Dubyaea* | *Dubyaea emeiensis* | NCBI | MK382842.1 | KF195991.1 | - | KF196123.1 | KF196035.1 | MK382874.1 | - |
| Asteraceae | Cichorioideae | Cichorieae | *Dubyaea* | *Dubyaea glaucescens* | NCBI | KF154382.1 | KF195997.1 | - | KF196129.1 | KF196041.1 | - | - |
| Asteraceae | Cichorioideae | Cichorieae | *Dubyaea* | *Dubyaea gombalana* | NCBI | MK043353.1 | - | - | MK046025.1 | - | MK046055.1 | - |
| Asteraceae | Cichorioideae | Cichorieae | *Dubyaea* | *Dubyaea hispida* | NCBI | MK043357.1 | KF195995.1 | - | KF196127.1 | JQ933313.1 | MK046059.1 | - |
| Asteraceae | Cichorioideae | Cichorieae | *Dubyaea* | *Dubyaea rubra* | NCBI | MK382843.1 | - | - | MK382888.1 | - | MK382884.1 | - |
| Asteraceae | Cichorioideae | Cichorieae | *Dubyaea* | *Dubyaea tsarongensis* | NCBI | MK382846.1 | KF195993.1 | - | KF196125.1 | KF196037.1 | MK382889.1 | - |
| Asteraceae | Asteroideae | Inuleae | *Duhaldea* | *Duhaldea cappa* | NCBI | KP092567.1 | KX526548.1 | LN607713.1 | EF211019.1 | KX527020.1 | - | - |
| Asteraceae | Asteroideae | Inuleae | *Duhaldea* | *Duhaldea eupatorioides* | NCBI | LN607443.1 | - | FM208929.1 | EF211009.1 | - | - | - |
| Asteraceae | Asteroideae | Inuleae | *Duhaldea* | *Duhaldea nervosa* | NCBI | EF210932.1 | - | FM208930.1 | EF210981.1 | - | - | - |
| Asteraceae | Asteroideae | Inuleae | *Duhaldea* | *Duhaldea rubricaulis* | NCBI | FM995372.1 | - | FM208931.1 | FM998666.1 | - | - | - |
| Asteraceae | Carduoideae | Cardueae | *Echinops* | *Echinops gmelinii* | NCBI | MH808124.1 | MN273585.1 | - | - | MN192516.1 | - | - |
| Asteraceae | Carduoideae | Cardueae | *Echinops* | *Echinops humilis* | NCBI | GU116514.1 | - | - | - | - | - | - |
| Asteraceae | Carduoideae | Cardueae | *Echinops* | *Echinops integrifolius* | NCBI | GU116517.1 | - | - | - | - | - | - |
| Asteraceae | Carduoideae | Cardueae | *Echinops* | *Echinops nanus* | NCBI | AY538654.1 | - | - | - | - | - | - |
| Asteraceae | Carduoideae | Cardueae | *Echinops* | *Echinops przewalskyi* | NCBI | AY914820.1 | - | - | AY914841.1 | - | - | - |
| Asteraceae | Carduoideae | Cardueae | *Echinops* | *Echinops ritro* | NCBI | MN918972.1 | EU385350.1 | EU385158.1 | - | EU384971.1 | JF272234.1 | - |
| Asteraceae | Carduoideae | Cardueae | *Echinops* | *Echinops setifer* | NCBI | GU116540.1 | - | - | - | - | - | - |
| Asteraceae | Carduoideae | Cardueae | *Echinops* | *Echinops sphaerocephalus* | NCBI | MN918973.1 | KC969526.1 | - | - | MW649086.1 | JF792579.1 | - |
| Asteraceae | Carduoideae | Cardueae | *Echinops* | *Echinops talassicus* | NCBI | AY538631.1 | - | - | - | - | - | - |
| Asteraceae | Carduoideae | Cardueae | *Echinops* | *Echinops tjanschanicus* | NCBI | GU116545.1 | - | - | - | - | - | - |
| Asteraceae | Asteroideae | Heliantheae | *Eclipta* | *Eclipta prostrata* | NCBI | MH711606.1 | MG641049.1 | AF384716.3 | MH152098.1 | JQ933318.1 | - | EU586869.1 |
| Asteraceae | Cichorioideae | Vernonieae | *Elephantopus* | *Elephantopus scaber* | NCBI | DQ813304.1 | JN407102.1 | HQ158507.1 | JN406936.2 | JQ933320.1 | - | - |
| Asteraceae | Cichorioideae | Vernonieae | *Elephantopus* | *Elephantopus tomentosus* | NCBI | EF108394.1 | JN407104.1 | HQ158504.1 | JN406940.2 | KX397783.1 | - | - |
| Asteraceae | Asteroideae | Senecioneae | *Emilia* | *Emilia coccinea* | NCBI | MG932777.1 | AF460026.1 | EF537964.1 | EF538029.1 | GU817758.1 | - | - |
| Asteraceae | Asteroideae | Senecioneae | *Emilia* | *Emilia fosbergii* | NCBI | GQ478091.1 | MH621585.1 | - | MH621889.1 | KJ773474.1 | - | - |
| Asteraceae | Asteroideae | Senecioneae | *Emilia* | *Emilia praetermissa* | NCBI | MG932776.1 | - | - | - | MW194982.1 | - | - |
| Asteraceae | Asteroideae | Senecioneae | *Emilia* | *Emilia prenanthoidea* | NCBI | EF538196.1 | MN783921.1 | - | - | MN783800.1 | - | - |
| Asteraceae | Asteroideae | Senecioneae | *Emilia* | *Emilia sonchifolia* | NCBI | LC503591.1 | LC503592.1 | - | LC503594.1 | LC503593.1 | KU750241.1 | - |
| Asteraceae | Asteroideae | Inuleae | *Epaltes* | *Epaltes australis* | NCBI | LN607444.1 | AY143606.1 | LN607796.1 | - | - | - | - |
| Asteraceae | Asteroideae | Inuleae | *Epaltes* | *Epaltes divaricata* | NCBI | LN607446.1 | - | LN607799.1 | - | - | - | - |
| Asteraceae | Cichorioideae | Cichorieae | *Epilasia* | *Epilasia hemilasia* | NCBI | AY508207.1 | - | - | - | - | - | - |
| Asteraceae | Asteroideae | Senecioneae | *Erechtites* | *Erechtites hieraciifolius* | NCBI | EF107652.1 | MH621597.1 | - | KP643306.1 | KJ773481.1 | - | - |
| Asteraceae | Asteroideae | Senecioneae | *Erechtites* | *Erechtites valerianifolius* | NCBI | EF108401.1 | EF537920.1 | EF537965.1 | EF538030.1 | JQ933328.1 | KU750242.1 | - |
| Asteraceae | Asteroideae | Astereae | *Erigeron* | *Erigeron acer* | NCBI | LC387648.1 | KP175121.1 | - | - | KJ746254.1 | - | - |
| Asteraceae | Asteroideae | Astereae | *Erigeron* | *Erigeron acris* | NCBI | OK376287.1 | KP175121.1 | - | - | KJ746254.1 | - | - |
| Asteraceae | Asteroideae | Astereae | *Erigeron* | *Erigeron altaicus* | NCBI | KJ711868.1 | - | - | - | - | - | - |
| Asteraceae | Asteroideae | Astereae | *Erigeron* | *Erigeron annuus* | NCBI | EF107653.1 | KX272483.1 | - | EU337693.1 | MH203367.1 | EU337663.1 | - |
| Asteraceae | Asteroideae | Astereae | *Erigeron* | *Erigeron bellioides* | NCBI | AF118522.1 | - | - | - | - | - | - |
| Asteraceae | Asteroideae | Astereae | *Erigeron* | *Erigeron breviscapus* | NCBI | GU213433.1 | - | - | - | KF482865.1 | - | - |
| Asteraceae | Asteroideae | Astereae | *Erigeron* | *Erigeron canadensis* | NCBI | OL853489.1 | XM | - | MH464294.1 | MH767498.1 | MH559528.1 | - |
| Asteraceae | Asteroideae | Astereae | *Erigeron* | *Erigeron elongatus* | NCBI | KJ711906.1 | - | - | - | - | - | - |
| Asteraceae | Asteroideae | Astereae | *Erigeron* | *Erigeron karvinskianus* | NCBI | AF118487.1 | KP175064.1 | - | - | JQ933521.1 | - | - |
| Asteraceae | Asteroideae | Astereae | *Erigeron* | *Erigeron lonchophyllus* | NCBI | AF118505.1 | MG225359.1 | MK800876.1 | - | MK749341.1 | - | - |
| Asteraceae | Asteroideae | Astereae | *Erigeron* | *Erigeron morrisonensis* | NCBI | EF107654.1 | MT384657.1 | - | - | - | - | - |
| Asteraceae | Asteroideae | Astereae | *Erigeron* | *Erigeron multiradiatus* | NCBI | ON527431.1 | - | - | - | JQ933329.1 | - | - |
| Asteraceae | Asteroideae | Astereae | *Erigeron* | *Erigeron strigosus* | NCBI | JF937689.1 | MG225023.1 | - | HQ596691.1 | MN601447.1 | - | - |
| Asteraceae | Asteroideae | Astereae | *Erigeron* | *Erigeron sumatrensis* | NCBI | KY968841.1 | KP175125.1 | - | HE966619.1 | MF135335.1 | - | - |
| Asteraceae | Cichorioideae | Vernonieae | *Ethulia* | *Ethulia conyzoides* | NCBI | EF155772.1 | - | EF155684.1 | - | - | - | - |
| Asteraceae | Asteroideae | Eupatorieae | *Eupatorium* | *Eupatorium chinense* | NCBI | MH808129.1 | - | - | - | - | - | - |
| Asteraceae | Asteroideae | Eupatorieae | *Eupatorium* | *Eupatorium formosanum* | NCBI | AB032032.1 | MT384669.1 | - | - | - | - | - |
| Asteraceae | Asteroideae | Eupatorieae | *Eupatorium* | *Eupatorium fortunei* | NCBI | GU724297.1 | AB217689.1 | - | GQ435109.1 | MN185074.1 | - | - |
| Asteraceae | Asteroideae | Eupatorieae | *Eupatorium* | *Eupatorium japonicum* | NCBI | AB032034.1 | MT384660.1 | - | - | - | - | - |
| Asteraceae | Asteroideae | Eupatorieae | *Eupatorium* | *Eupatorium lindleyanum* | NCBI | MH710778.1 | AB217690.1 | - | - | MH657547.1 | - | - |
| Asteraceae | Asteroideae | Astereae | *Eurybia* | *Eurybia sibirica* | NCBI | AY772421.1 | MG225201.1 | - | - | MG222926.1 | - | GU480733.1 |
| Asteraceae | Cichorioideae | Cichorieae | *Faberia* | *Faberia cavaleriei* | NCBI | KF739618.1 | KF196012.1 | - | KF196144.1 | KF739633.1 | - | - |
| Asteraceae | Cichorioideae | Cichorieae | *Faberia* | *Faberia faberi* | NCBI | KF485546.1 | KF196013.1 | - | KF196143.1 | KF739634.1 | - | - |
| Asteraceae | Cichorioideae | Cichorieae | *Faberia* | *Faberia nanchuanensis* | NCBI | KF739614.1 | KF739650.1 | - | KF739623.1 | KF739632.1 | KF732175.1 | - |
| Asteraceae | Cichorioideae | Cichorieae | *Faberia* | *Faberia sinensis* | NCBI | KF485543.1 | KF739652.1 | - | KF196149.1 | KF196061.1 | - | - |
| Asteraceae | Cichorioideae | Cichorieae | *Faberia* | *Faberia thibetica* | NCBI | KF751832.1 | KF739653.1 | - | KF739626.1 | KF196062.1 | KF732174.1 | - |
| Asteraceae | Asteroideae | Senecioneae | *Farfugium* | *Farfugium japonicum* | NCBI | GU724279.1 | KX526553.1 | AY723242.1 | GU818374.1 | KX527289.1 | KU750243.1 | KY970495.1 |
| Asteraceae | Asteroideae | Gnaphalieae | *Filago* | *Filago arvensis* | NCBI | FN645886.1 | KX676802.1 | - | - | KX678888.1 | - | - |
| Asteraceae | Asteroideae | Tageteae | *Flaveria* | *Flaveria bidentis* | NCBI | DQ122544.1 | - | HQ534109.1 | - | X55830.1 | - | - |
| Asteraceae | Asteroideae | Astereae | *Formania* | *Formania mekongensis* | NCBI | AY572951.1 | - | KX526891.1 | - | KX527027.1 | - | - |
| Asteraceae | Carduoideae | Cardueae | *Frolovia* | *Frolovia frolowii* | NCBI | AY914822.1 | JQ303114.1 | - | AY914842.1 | - | - | - |
| Asteraceae | Asteroideae | Coreopsideae | *Gaillardia* | *Gaillardia pulchella* | NCBI | EF108395.1 | HQ593303.1 | - | OK469564.1 | KJ773526.1 | - | - |
| Asteraceae | Asteroideae | Astereae | *Galatella* | *Galatella altaica* | NCBI | KJ711905.1 | - | - | - | - | - | - |
| Asteraceae | Asteroideae | Astereae | *Galatella* | *Galatella angustissima* | NCBI | KJ711880.1 | - | - | - | - | - | - |
| Asteraceae | Asteroideae | Astereae | *Galatella* | *Galatella biflora* | NCBI | KJ711882.1 | - | - | - | - | - | - |
| Asteraceae | Asteroideae | Astereae | *Galatella* | *Galatella dahurica* | NCBI | JN315935.1 | - | - | - | - | - | - |
| Asteraceae | Asteroideae | Astereae | *Galatella* | *Galatella hauptii* | NCBI | KJ711878.1 | - | - | - | - | - | - |
| Asteraceae | Asteroideae | Astereae | *Galatella* | *Galatella punctata* | NCBI | LC027394.1 | - | - | - | - | - | - |
| Asteraceae | Asteroideae | Astereae | *Galatella* | *Galatella scoparia* | NCBI | LC027395.1 | - | - | - | - | - | - |
| Asteraceae | Asteroideae | Millieae | *Galinsoga* | *Galinsoga parviflora* | NCBI | FJ696962.1 | MT214821.1 | - | DQ006151.1 | KM360797.1 | - | - |
| Asteraceae | Asteroideae | Millieae | *Galinsoga* | *Galinsoga quadriradiata* | NCBI | GU818550.1 | AY215801.1 | AF384727.3 | MF143660.1 | GU817762.1 | - | - |
| Asteraceae | Asteroideae | Gnaphalieae | *Gamochaeta* | *Gamochaeta coarctata* | NCBI | KT865473.1 | HM445618.1 | HM445710.1 | - | - | - | - |
| Asteraceae | Asteroideae | Gnaphalieae | *Gamochaeta* | *Gamochaeta pensylvanica* | NCBI | JX524600.1 | EU385354.1 | EU385162.1 | - | EU384977.1 | - | - |
| Asteraceae | Asteroideae | Gnaphalieae | *Gamochaeta* | *Gamochaeta purpurea* | NCBI | AY947411.1 | HM850638.1 | - | MK260969.1 | HM850036.1 | - | - |
| Asteraceae | Mutisioideae | Mutisieae | *Gerbera* | *Gerbera delavayi* | NCBI | KX932430.1 | - | - | - | - | - | - |
| Asteraceae | Mutisioideae | Mutisieae | *Gerbera* | *Gerbera maxima* | NCBI | KX349402.1 | - | - | - | - | - | - |
| Asteraceae | Mutisioideae | Mutisieae | *Gerbera* | *Gerbera nivea* | NCBI | MG661703.1 | - | - | - | - | - | - |
| Asteraceae | Asteroideae | Anthemideae | *Glebionis* | *Glebionis coronaria* | NCBI | EF577292.1 | KX401337.1 | EU334465.1 | EF091603.1 | HM849892.1 | - | - |
| Asteraceae | Asteroideae | Anthemideae | *Glebionis* | *Glebionis segetum* | NCBI | AF218883.1 | JN896039.1 | - | - | KM360717.1 | - | - |
| Asteraceae | Asteroideae | Tageteae | *Glossocardia* | *Glossocardia bidens* | NCBI | JX564727.1 | JX564839.1 | - | MK546196.1 | - | - | - |
| Asteraceae | Asteroideae | Gnaphalieae | *Gnaphalium* | *Gnaphalium polycaulon* | NCBI | KT865478.1 | - | - | - | - | - | - |
| Asteraceae | Asteroideae | Gnaphalieae | *Gnaphalium* | *Gnaphalium supinum* | NCBI | AY445230.1 | HM445621.1 | HM445683.1 | - | KF997337.1 | - | - |
| Asteraceae | Asteroideae | Gnaphalieae | *Gnaphalium* | *Gnaphalium uliginosum* | NCBI | FN645823.1 | HM445642.1 | HM445680.1 | - | KM360808.1 | - | - |
| Asteraceae | Asteroideae | Astereae | *Grindelia* | *Grindelia squarrosa* | NCBI | JQ011937.1 | KT176593.1 | KT176833.1 | - | KT178113.1 | KT179027.1 | - |
| Asteraceae | Asteroideae | Millieae | *Guizotia* | *Guizotia abyssinica* | NCBI | KY397487.1 | AM411125.1 | - | - | KM360809.1 | FJ589153.1 | - |
| Asteraceae | Asteroideae | Eupatorieae | *Gymnocoronis* | *Gymnocoronis spilanthoides* | NCBI | MH050167.1 | KJ637188.1 | KP454940.1 | - | MH049938.1 | - | - |
| Asteraceae | Asteroideae | Senecioneae | *Gynura* | *Gynura bicolor* | NCBI | LC716853.1 | GQ434114.1 | - | LC716855.1 | GQ436450.1 | - | - |
| Asteraceae | Asteroideae | Senecioneae | *Gynura* | *Gynura japonica* | NCBI | MH712680.1 | KX526555.1 | KX526880.1 | - | KX527000.1 | - | - |
| Asteraceae | Asteroideae | Senecioneae | *Gynura* | *Gynura procumbens* | NCBI | FJ980339.1 | HM989778.1 | - | MH069932.1 | GQ436466.1 | - | - |
| Asteraceae | Asteroideae | Anthemideae | *Handelia* | *Handelia trichophylla* | NCBI | LC313948.1 | - | - | AB683354.1 | - | - | - |
| Asteraceae | Asteroideae | Heliantheae | *Helianthus* | *Helianthus debilis* | NCBI | JX121488.1 | MF350116.1 | - | MF348525.1 | KJ773553.1 | - | - |
| Asteraceae | Asteroideae | Heliantheae | *Helianthus* | *Helianthus tuberosus* | NCBI | KY968959.1 | AY009464.1 | KT176829.1 | GU818386.1 | GU817765.1 | KT179023.1 | - |
| Asteraceae | Asteroideae | Gnaphalieae | *Helichrysum* | *Helichrysum arenarium* | NCBI | AY445207.1 | KJ746174.1 | - | - | - | - | - |
| Asteraceae | Asteroideae | Gnaphalieae | *Helichrysum* | *Helichrysum thianschanicum* | NCBI | AY445200.1 | - | HG797959.1 | - | - | - | - |
| Asteraceae | Carduoideae | Cardueae | *Hemistepta* | *Hemistepta lyrata* | NCBI | AB118139.1 | AB118153.1 | AB254899.1 | AY914844.1 | JQ933358.1 | - | - |
| Asteraceae | Cichorioideae | Cichorieae | *Heteracia* | *Heteracia szovitsii* | NCBI | AJ633283.1 | KX526556.1 | - | HQ436161.1 | KX527542.1 | - | - |
| Asteraceae | Asteroideae | Astereae | *Heteroplexis* | *Heteroplexis impressinervia* | NCBI | MW703710.1 | - | - | - | - | - | - |
| Asteraceae | Asteroideae | Astereae | *Heteroplexis* | *Heteroplexis incana* | NCBI | MW703708.1 | - | - | - | - | - | - |
| Asteraceae | Asteroideae | Astereae | *Heteroplexis* | *Heteroplexis microcephala* | NCBI | MW703707.1 | - | - | - | - | - | - |
| Asteraceae | Asteroideae | Astereae | *Heteroplexis* | *Heteroplexis sericophylla* | NCBI | MW703709.1 | - | - | - | - | - | - |
| Asteraceae | Asteroideae | Astereae | *Heteroplexis* | *Heteroplexis vernonioides* | NCBI | MW703706.1 | - | - | - | - | - | - |
| Asteraceae | Cichorioideae | Cichorieae | *Hieracium* | *Hieracium umbellatum* | NCBI | KM372116.1 | KX526557.1 | KX526885.1 | KF196107.1 | JQ933362.1 | - | - |
| Asteraceae | Cichorioideae | Cichorieae | *Hieracium* | *Hieracium virosum* | NCBI | MH808135.1 | MK435711.1 | - | - | MN204742.1 | - | - |
| Asteraceae | Carduoideae | Cardueae | *Himalaiella* | *Himalaiella auriculata* | NCBI | AB118111.1 | AB118142.1 | - | - | - | - | - |
| Asteraceae | Carduoideae | Cardueae | *Himalaiella* | *Himalaiella deltoidea* | NCBI | AB118114.1 | AB118143.1 | - | HQ690928.1 | - | - | - |
| Asteraceae | Carduoideae | Cardueae | *Himalaiella* | *Himalaiella nivea* | NCBI | GU724304.1 | HM989798.1 | - | GQ435137.1 | GQ436489.1 | - | - |
| Asteraceae | Cichorioideae | Cichorieae | *Hypochaeris* | *Hypochaeris albiflora* | NCBI | KT735373.1 | - | - | - | - | - | - |
| Asteraceae | Cichorioideae | Cichorieae | *Hypochaeris* | *Hypochaeris chillensis* | NCBI | KT735379.1 | AF528407.1 | - | - | HQ644047.1 | AJ627456.1 | - |
| Asteraceae | Cichorioideae | Cichorieae | *Hypochaeris* | *Hypochaeris ciliata* | NCBI | AF528449.1 | MK435713.1 | - | - | MN204744.1 | - | - |
| Asteraceae | Cichorioideae | Cichorieae | *Hypochaeris* | *Hypochaeris glabra* | NCBI | MF405670.1 | AJ633232.1 | AY504734.1 | - | HM850068.1 | MF405716.1 | - |
| Asteraceae | Cichorioideae | Cichorieae | *Hypochaeris* | *Hypochaeris maculata* | NCBI | AF528453.1 | AF528413.1 | - | JX501950.1 | MK925396.1 | AJ627468.1 | - |
| Asteraceae | Cichorioideae | Cichorieae | *Hypochaeris* | *Hypochaeris radicata* | NCBI | AF528460.1 | AF528421.1 | KX526910.1 | FJ395488.1 | KM360829.1 | MF405724.1 | - |
| Asteraceae | Asteroideae | Inuleae | *Inula* | *Inula britannica* | NCBI | KY696297.1 | AY215812.1 | AY780826.1 | AY215561.1 | AY215130.1 | - | - |
| Asteraceae | Asteroideae | Inuleae | *Inula* | *Inula caspica* | NCBI | LN607475.1 | - | LN607647.1 | - | - | - | - |
| Asteraceae | Asteroideae | Inuleae | *Inula* | *Inula helenium* | NCBI | KY696284.1 | AF151473.1 | FM208945.1 | HQ596734.1 | HQ590141.1 | - | - |
| Asteraceae | Asteroideae | Inuleae | *Inula* | *Inula hupehensis* | NCBI | MW116496.1 | - | - | MW137573.1 | - | - | - |
| Asteraceae | Asteroideae | Inuleae | *Inula* | *Inula japonica* | NCBI | MH710804.1 | HM989790.1 | - | GU724262.1 | GU724238.1 | - | - |
| Asteraceae | Asteroideae | Inuleae | *Inula* | *Inula linariifolia* | NCBI | MW116497.1 | - | - | MW137574.1 | MN204746.1 | - | - |
| Asteraceae | Asteroideae | Inuleae | *Inula* | *Inula obtusifolia* | NCBI | LN607479.1 | - | LN607654.1 | - | - | - | - |
| Asteraceae | Asteroideae | Inuleae | *Inula* | *Inula racemosa* | NCBI | MH808140.1 | MK435714.1 | - | - | MN185077.1 | - | - |
| Asteraceae | Asteroideae | Inuleae | *Inula* | *Inula salicina* | NCBI | KY696323.1 | LC628089.1 | FM208961.1 | FR865070.1 | FR865116.1 | - | - |
| Asteraceae | Cichorioideae | Cichorieae | *Ixeridium* | *Ixeridium beauverdianum* | NCBI | AB972273.1 | - | - | - | JQ933369.1 | - | - |
| Asteraceae | Cichorioideae | Cichorieae | *Ixeridium* | *Ixeridium dentatum* | NCBI | MH711064.1 | LC625506.1 | - | LC365348.1 | MH203373.1 | - | - |
| Asteraceae | Cichorioideae | Cichorieae | *Ixeridium* | *Ixeridium laevigatum* | NCBI | AY862582.1 | - | - | - | - | - | - |
| Asteraceae | Cichorioideae | Cichorieae | *Ixeridium* | *Ixeridium transnokoense* | NCBI | AB972297.1 | KX526559.1 | - | - | KX527110.1 | - | - |
| Asteraceae | Cichorioideae | Cichorieae | *Ixeris* | *Ixeris chinensis* | NCBI | MH808144.1 | KX526560.1 | KX526887.1 | KJ025052.1 | KX527023.1 | KC968144.1 | - |
| Asteraceae | Cichorioideae | Cichorieae | *Ixeris* | *Ixeris japonica* | NCBI | AB972298.1 | - | - | - | - | - | - |
| Asteraceae | Cichorioideae | Cichorieae | *Ixeris* | *Ixeris polycephala* | NCBI | MH808145.1 | KT250087.1 | - | - | KU958562.1 | - | - |
| Asteraceae | Cichorioideae | Cichorieae | *Ixeris* | *Ixeris repens* | NCBI | HQ436225.1 | MT384652.1 | - | - | - | - | - |
| Asteraceae | Cichorioideae | Cichorieae | *Ixeris* | *Ixeris stolonifera* | NCBI | AB766226.1 | AJ633156.1 | - | KF739629.1 | - | - | - |
| Asteraceae | Cichorioideae | Cichorieae | *Ixeris* | *Ixeris tamagawaensis* | NCBI | KT634252.1 | MT384666.1 | - | - | - | - | - |
| Asteraceae | Carduoideae | Cardueae | *Jurinea* | *Jurinea lanipes* | NCBI | LS974053.1 | AY373686.1 | - | - | - | - | - |
| Asteraceae | Carduoideae | Cardueae | *Jurinea* | *Jurinea multiflora* | NCBI | MH003704.1 | LT898399.1 | - | MH070742.1 | MH070869.1 | MH070995.1 | - |
| Asteraceae | Asteroideae | Inuleae | *Karelinia* | *Karelinia caspia* | NCBI | LN607483.1 | - | LN607658.1 | - | - | - | - |
| Asteraceae | Asteroideae | Anthemideae | *Kaschgaria* | *Kaschgaria komarovii* | NCBI | JX051764.1 | - | AF153631.1 | JX073912.1 | - | - | - |
| Asteraceae | Carduoideae | Cardueae | *Klasea* | *Klasea dissecta* | NCBI | MN443046.1 | MT862706.1 | - | - | - | - | - |
| Asteraceae | Cichorioideae | Cichorieae | *Koelpinia* | *Koelpinia linearis* | NCBI | AJ633492.1 | AJ633263.1 | AF218341.1 | JX501951.1 | KX282827.1 | - | - |
| Asteraceae | Cichorioideae | Cichorieae | *Lactuca* | *Lactuca dissecta* | NCBI | KF485649.1 | EU363580.1 | - | MF785890.1 | MF786672.1 | - | - |
| Asteraceae | Cichorioideae | Cichorieae | *Lactuca* | *Lactuca dolichophylla* | NCBI | KF485650.1 | GU109314.1 | - | GU109328.1 | KT308114.1 | - | - |
| Asteraceae | Cichorioideae | Cichorieae | *Lactuca* | *Lactuca formosana* | NCBI | MH711571.1 | MH660122.1 | - | GU109333.1 | MH658654.1 | - | - |
| Asteraceae | Cichorioideae | Cichorieae | *Lactuca* | *Lactuca orientalis* | NCBI | OP070103.1 | - | - | KF485915.1 | - | - | - |
| Asteraceae | Cichorioideae | Cichorieae | *Lactuca* | *Lactuca raddeana* | NCBI | KF485658.1 | MH659923.1 | - | KF485914.1 | MH658447.1 | - | - |
| Asteraceae | Cichorioideae | Cichorieae | *Lactuca* | *Lactuca serriola* | NCBI | KF850588.1 | GU109315.1 | - | HQ596742.1 | KM360838.1 | LS975624.1 | - |
| Asteraceae | Cichorioideae | Cichorieae | *Lactuca* | *Lactuca sibirica* | NCBI | OP070085.1 | KU586769.1 | - | LS991547.1 | - | LS975604.1 | - |
| Asteraceae | Cichorioideae | Cichorieae | *Lactuca* | *Lactuca tatarica* | NCBI | MH808146.1 | MK435719.1 | - | GU109331.1 | MN623758.1 | LS975602.1 | - |
| Asteraceae | Cichorioideae | Cichorieae | *Lactuca* | *Lactuca undulata* | NCBI | OP070077.1 | MK954290.1 | - | KF485904.1 | MK954400.1 | - | - |
| Asteraceae | Asteroideae | Astereae | *Lagenophora* | *Lagenophora stipitata* | NCBI | AB435145.1 | AB436888.1 | - | - | - | - | - |
| Asteraceae | Asteroideae | Inuleae | *Laggera* | *Laggera alata* | NCBI | FJ980335.1 | - | - | EF210979.1 | JQ933375.1 | - | - |
| Asteraceae | Asteroideae | Inuleae | *Laggera* | *Laggera crispata* | NCBI | LN607485.1 | - | LN607780.1 | EF210977.1 | - | - | - |
| Asteraceae | Asteroideae | Inuleae | *Laggera* | *Laggera pterodonta* | NCBI | EF210929.1 | - | AY780862.1 | EF210978.1 | - | - | - |
| Asteraceae | Cichorioideae | Cichorieae | *Lapsanastrum* | *Lapsanastrum apogonoides* | NCBI | MH808148.1 | - | - | - | MN185079.1 | - | - |
| Asteraceae | Cichorioideae | Cichorieae | *Launaea* | *Launaea sarmentosa* | NCBI | MZ292982.1 | DQ507983.1 | - | KF485792.1 | MH767501.1 | - | - |
| Asteraceae | Mutisioideae | Mutisieae | *Leibnitzia* | *Leibnitzia anandria* | NCBI | GU724275.1 | HM989740.1 | L39402.1 | GQ435075.1 | GQ436437.1 | - | AY865228.1 |
| Asteraceae | Asteroideae | Gnaphalieae | *Leontopodium* | *Leontopodium andersonii* | NCBI | FJ639921.1 | FJ640038.1 | - | - | - | - | - |
| Asteraceae | Asteroideae | Gnaphalieae | *Leontopodium* | *Leontopodium artemisiifolium* | NCBI | FJ639923.1 | - | - | - | - | - | - |
| Asteraceae | Asteroideae | Gnaphalieae | *Leontopodium* | *Leontopodium caespitosum* | NCBI | FJ639927.1 | FJ640025.1 | - | - | - | - | - |
| Asteraceae | Asteroideae | Gnaphalieae | *Leontopodium* | *Leontopodium calocephalum* | NCBI | MH808149.1 | FJ640026.1 | - | - | - | - | - |
| Asteraceae | Asteroideae | Gnaphalieae | *Leontopodium* | *Leontopodium chui* | NCBI | JQ895513.1 | - | - | - | - | - | - |
| Asteraceae | Asteroideae | Gnaphalieae | *Leontopodium* | *Leontopodium dedekensii* | NCBI | FJ639932.1 | - | - | - | - | - | - |
| Asteraceae | Asteroideae | Gnaphalieae | *Leontopodium* | *Leontopodium forrestianum* | NCBI | KT865533.1 | - | - | - | - | - | - |
| Asteraceae | Asteroideae | Gnaphalieae | *Leontopodium* | *Leontopodium franchetii* | NCBI | FJ639935.1 | - | - | - | - | - | - |
| Asteraceae | Asteroideae | Gnaphalieae | *Leontopodium* | *Leontopodium haastioides* | NCBI | KT865535.1 | - | - | - | - | - | - |
| Asteraceae | Asteroideae | Gnaphalieae | *Leontopodium* | *Leontopodium himalayanum* | NCBI | FJ639938.1 | - | - | - | - | - | - |
| Asteraceae | Asteroideae | Gnaphalieae | *Leontopodium* | *Leontopodium jacotianum* | NCBI | FJ639942.1 | - | - | - | JQ933384.1 | - | - |
| Asteraceae | Asteroideae | Gnaphalieae | *Leontopodium* | *Leontopodium japonicum* | NCBI | MH808150.1 | MK435720.1 | - | - | - | KP713377.1 | - |
| Asteraceae | Asteroideae | Gnaphalieae | *Leontopodium* | *Leontopodium leontopodioides* | NCBI | FJ980329.1 | FJ640027.1 | - | GQ435092.1 | GQ436452.1 | - | - |
| Asteraceae | Asteroideae | Gnaphalieae | *Leontopodium* | *Leontopodium microphyllum* | NCBI | KT865539.1 | GU943480.1 | - | GU943342.1 | - | GU943390.1 | - |
| Asteraceae | Asteroideae | Gnaphalieae | *Leontopodium* | *Leontopodium nanum* | NCBI | MH808152.1 | FJ640029.1 | - | - | MN185080.1 | - | - |
| Asteraceae | Asteroideae | Gnaphalieae | *Leontopodium* | *Leontopodium niveum* | NCBI | KT865541.1 | - | - | - | - | - | - |
| Asteraceae | Asteroideae | Gnaphalieae | *Leontopodium* | *Leontopodium ochroleucum* | NCBI | KT865542.1 | GU943485.1 | - | GU943344.1 | - | GU943407.1 | - |
| Asteraceae | Asteroideae | Gnaphalieae | *Leontopodium* | *Leontopodium pusillum* | NCBI | FJ639956.1 | FJ640034.1 | - | - | - | - | - |
| Asteraceae | Asteroideae | Gnaphalieae | *Leontopodium* | *Leontopodium sinense* | NCBI | FJ639958.1 | FJ640035.1 | - | - | - | - | - |
| Asteraceae | Asteroideae | Gnaphalieae | *Leontopodium* | *Leontopodium souliei* | NCBI | MH808154.1 | MK435721.1 | - | - | MK749923.1 | - | - |
| Asteraceae | Asteroideae | Gnaphalieae | *Leontopodium* | *Leontopodium stoechas* | NCBI | KT865545.1 | - | - | - | - | - | - |
| Asteraceae | Asteroideae | Gnaphalieae | *Leontopodium* | *Leontopodium stracheyi* | NCBI | KT865546.1 | FJ640037.1 | - | - | - | - | - |
| Asteraceae | Asteroideae | Gnaphalieae | *Leontopodium* | *Leontopodium subulatum* | NCBI | KT865547.1 | - | - | - | - | - | - |
| Asteraceae | Asteroideae | Anthemideae | *Leucanthemella* | *Leucanthemella linearis* | NCBI | KJ183129.1 | - | - | - | - | - | - |
| Asteraceae | Asteroideae | Anthemideae | *Leucanthemum* | *Leucanthemum vulgare* | NCBI | MK481551.1 | HQ593344.1 | EU334481.1 | EF091629.1 | MG222974.1 | KY778057.1 | - |
| Asteraceae | Wunderlichioideae | Hyalideae | *Leucomeris* | *Leucomeris decora* | NCBI | - | KX438067.1 | KX438071.1 | - | KX438075.1 | HQ671097.1 | - |
| Asteraceae | Asteroideae | Senecioneae | *Ligularia* | *Ligularia anoleuca* | NCBI | AB557884.1 | - | - | - | - | - | - |
| Asteraceae | Asteroideae | Senecioneae | *Ligularia* | *Ligularia atroviolacea* | NCBI | DQ272322.1 | - | - | - | - | - | KY970577.1 |
| Asteraceae | Asteroideae | Senecioneae | *Ligularia* | *Ligularia botryodes* | NCBI | MH808155.1 | - | - | - | - | - | - |
| Asteraceae | Asteroideae | Senecioneae | *Ligularia* | *Ligularia caloxantha* | NCBI | DQ272345.1 | - | - | - | - | - | - |
| Asteraceae | Asteroideae | Senecioneae | *Ligularia* | *Ligularia curvisquama* | NCBI | DQ272324.1 | - | - | - | - | - | KY970547.1 |
| Asteraceae | Asteroideae | Senecioneae | *Ligularia* | *Ligularia cyathiceps* | NCBI | DQ272328.1 | - | - | KX779178.1 | - | KX779148.1 | KY970444.1 |
| Asteraceae | Asteroideae | Senecioneae | *Ligularia* | *Ligularia cymbulifera* | NCBI | KM036266.1 | JF954341.1 | AY723222.1 | JN045185.1 | JF942252.1 | KU750246.1 | KY970390.1 |
| Asteraceae | Asteroideae | Senecioneae | *Ligularia* | *Ligularia dentata* | NCBI | MH808156.1 | MK435722.1 | AY723219.1 | DQ131856.1 | KR080513.1 | - | KY970378.1 |
| Asteraceae | Asteroideae | Senecioneae | *Ligularia* | *Ligularia dictyoneura* | NCBI | JF976815.1 | JF954344.1 | - | JN045190.1 | JF942255.1 | KU750248.1 | KY970516.1 |
| Asteraceae | Asteroideae | Senecioneae | *Ligularia* | *Ligularia dolichobotrys* | NCBI | MH711312.1 | MN251235.1 | - | - | MN185081.1 | - | KY970440.1 |
| Asteraceae | Asteroideae | Senecioneae | *Ligularia* | *Ligularia duciformis* | NCBI | KY307735.1 | MF786893.1 | - | MF785893.1 | MF786644.1 | - | KY970497.1 |
| Asteraceae | Asteroideae | Senecioneae | *Ligularia* | *Ligularia_dux* | NCBI | - | - | - | - | - | - | KY970579.1 |
| Asteraceae | Asteroideae | Senecioneae | *Ligularia* | *Ligularia fischeri* | NCBI | MH808160.1 | MK435723.1 | - | AB369636.1 | MN192527.1 | - | KY970393.1 |
| Asteraceae | Asteroideae | Senecioneae | *Ligularia* | *Ligularia franchetiana* | NCBI | AB375311.1 | - | - | - | - | KU750249.1 | KY970503.1 |
| Asteraceae | Asteroideae | Senecioneae | *Ligularia* | *Ligularia hodgsonii* | NCBI | FJ980336.1 | MK435724.1 | - | HQ419274.1 | MN185082.1 | KU750250.1 | - |
| Asteraceae | Asteroideae | Senecioneae | *Ligularia* | *Ligularia hookeri* | NCBI | MH117590.1 | MK435727.1 | - | JN045193.1 | MH116229.1 | - | KY970438.1 |
| Asteraceae | Asteroideae | Senecioneae | *Ligularia* | *Ligularia intermedia* | NCBI | KY979066.1 | MK435728.1 | - | JF708226.1 | - | - | KY970433.1 |
| Asteraceae | Asteroideae | Senecioneae | *Ligularia* | *Ligularia jaluensis* | NCBI | KU696033.1 | - | - | - | - | KU750251.1 | KY970397.1 |
| Asteraceae | Asteroideae | Senecioneae | *Ligularia* | *Ligularia jamesii* | NCBI | KY979038.1 | - | - | - | - | - | KY970398.1 |
| Asteraceae | Asteroideae | Senecioneae | *Ligularia* | *Ligularia japonica* | NCBI | AY458825.1 | - | - | - | - | - | KY970432.1 |
| Asteraceae | Asteroideae | Senecioneae | *Ligularia* | *Ligularia kanaitzensis* | NCBI | JF976816.1 | JF954349.1 | - | JN045196.1 | JF942260.1 | - | KY970520.1 |
| Asteraceae | Asteroideae | Senecioneae | *Ligularia* | *Ligularia lamarum* | NCBI | KY307742.1 | - | AY723225.1 | KY307383.1 | - | - | KY970415.1 |
| Asteraceae | Asteroideae | Senecioneae | *Ligularia* | *Ligularia lankongensis* | NCBI | JF976819.1 | JF954351.1 | - | JN045198.1 | JF942262.1 | - | KY970523.1 |
| Asteraceae | Asteroideae | Senecioneae | *Ligularia* | *Ligularia lapathifolia* | NCBI | KY979063.1 | - | - | - | - | - | KY970430.1 |
| Asteraceae | Asteroideae | Senecioneae | *Ligularia* | *Ligularia latihastata* | NCBI | DQ272334.1 | - | - | - | - | - | KY970517.1 |
| Asteraceae | Asteroideae | Senecioneae | *Ligularia* | *Ligularia liatroides* | NCBI | AY723268.1 | - | AY723231.1 | - | - | - | KY970428.1 |
| Asteraceae | Asteroideae | Senecioneae | *Ligularia* | *Ligularia lidjiangensis* | NCBI | MT914296.1 | MT928688.1 | - | - | MT921021.1 | - | KY970439.1 |
| Asteraceae | Asteroideae | Senecioneae | *Ligularia* | *Ligularia lingiana* | NCBI | JF767247.1 | - | - | - | - | JF767227.1 | - |
| Asteraceae | Asteroideae | Senecioneae | *Ligularia* | *Ligularia longihastata* | NCBI | DQ272339.1 | - | - | - | - | - | - |
| Asteraceae | Asteroideae | Senecioneae | *Ligularia* | *Ligularia microcephala* | NCBI | MH293151.1 | MH293225.1 | - | - | MH293293.1 | - | - |
| Asteraceae | Asteroideae | Senecioneae | *Ligularia* | *Ligularia narynensis* | NCBI | AY456130.1 | - | - | - | - | - | MT240744.1 |
| Asteraceae | Asteroideae | Senecioneae | *Ligularia* | *Ligularia nelumbifolia* | NCBI | JF767251.1 | JF954353.1 | - | JN045200.1 | JF942264.1 | MF688711.1 | KY970518.1 |
| Asteraceae | Asteroideae | Senecioneae | *Ligularia* | *Ligularia oligonema* | NCBI | KY979041.1 | - | - | - | - | - | KY970401.1 |
| Asteraceae | Asteroideae | Senecioneae | *Ligularia* | *Ligularia paradoxa* | NCBI | KY307784.1 | - | - | KY307412.1 | - | - | - |
| Asteraceae | Asteroideae | Senecioneae | *Ligularia* | *Ligularia platyglossa* | NCBI | KY979096.1 | - | - | - | - | - | KY970519.1 |
| Asteraceae | Asteroideae | Senecioneae | *Ligularia* | *Ligularia pleurocaulis* | NCBI | AB212735.1 | KT280229.1 | AY723230.1 | JN045202.1 | KT280124.1 | - | KY970379.1 |
| Asteraceae | Asteroideae | Senecioneae | *Ligularia* | *Ligularia przewalskii* | NCBI | KY397492.1 | MK435731.1 | AY723226.1 | - | MN204755.1 | - | KY970414.1 |
| Asteraceae | Asteroideae | Senecioneae | *Ligularia* | *Ligularia pubifolia* | NCBI | GU444022.1 | - | - | - | - | - | - |
| Asteraceae | Asteroideae | Senecioneae | *Ligularia* | *Ligularia purdomii* | NCBI | AY723257.1 | - | AY723220.1 | - | - | - | KY970434.1 |
| Asteraceae | Asteroideae | Senecioneae | *Ligularia* | *Ligularia rumicifolia* | NCBI | AY723260.1 | - | AY723223.1 | - | - | - | - |
| Asteraceae | Asteroideae | Senecioneae | *Ligularia* | *Ligularia sagitta* | NCBI | MH808169.1 | MK435732.1 | AY723228.1 | MF785966.1 | MF786754.1 | KU750252.1 | KY970416.1 |
| Asteraceae | Asteroideae | Senecioneae | *Ligularia* | *Ligularia sibirica* | NCBI | AY176146.1 | - | - | - | - | - | KY970496.1 |
| Asteraceae | Asteroideae | Senecioneae | *Ligularia* | *Ligularia songarica* | NCBI | MT240670.1 | - | - | - | - | - | MT240740.1 |
| Asteraceae | Asteroideae | Senecioneae | *Ligularia* | *Ligularia stenocephala* | NCBI | AF459961.1 | AF460023.1 | EF537975.2 | EF538078.1 | - | KU750253.1 | KY970514.1 |
| Asteraceae | Asteroideae | Senecioneae | *Ligularia* | *Ligularia stenoglossa* | NCBI | AB523365.1 | - | - | - | - | - | KY970437.1 |
| Asteraceae | Asteroideae | Senecioneae | *Ligularia* | *Ligularia subspicata* | NCBI | KY307785.1 | JF954361.1 | - | JN045206.1 | JF942272.1 | KU750254.1 | KY970435.1 |
| Asteraceae | Asteroideae | Senecioneae | *Ligularia* | *Ligularia thomsonii* | NCBI | MT240677.1 | - | - | - | - | - | - |
| Asteraceae | Asteroideae | Senecioneae | *Ligularia* | *Ligularia thyrsoidea* | NCBI | MT240673.1 | - | - | - | - | - | MT240746.1 |
| Asteraceae | Asteroideae | Senecioneae | *Ligularia* | *Ligularia tianschanica* | NCBI | MT240671.1 | - | - | - | MF158770.1 | - | MT240741.1 |
| Asteraceae | Asteroideae | Senecioneae | *Ligularia* | *Ligularia tsangchanensis* | NCBI | AB284129.1 | - | AY723227.1 | - | - | KU750256.1 | KY970399.1 |
| Asteraceae | Asteroideae | Senecioneae | *Ligularia* | *Ligularia veitchiana* | NCBI | MH808171.1 | MK435735.1 | - | - | - | KU750257.1 | KY970413.1 |
| Asteraceae | Asteroideae | Senecioneae | *Ligularia* | *Ligularia vellerea* | NCBI | KM036281.1 | JF954368.1 | AY723224.1 | JQ220169.1 | JF942279.1 | KU750258.1 | KY970389.1 |
| Asteraceae | Asteroideae | Senecioneae | *Ligularia* | *Ligularia villosa* | NCBI | KY979070.1 | - | - | - | - | - | KY970445.1 |
| Asteraceae | Asteroideae | Senecioneae | *Ligularia* | *Ligularia virgaurea* | NCBI | JF976840.1 | KT280230.1 | - | JN045219.1 | KT280125.1 | KU750259.1 | KY970400.1 |
| Asteraceae | Asteroideae | Senecioneae | *Ligularia* | *Ligularia wilsoniana* | NCBI | KY979069.1 | - | - | - | - | - | KY970441.1 |
| Asteraceae | Asteroideae | Senecioneae | *Ligularia* | *Ligularia yunnanensis* | NCBI | AY723258.1 | - | AY723221.1 | KX779194.1 | - | MF688695.1 | KY970392.1 |
| Asteraceae | Asteroideae | Senecioneae | *Ligulariopsis* | *Ligulariopsis shichuana* | NCBI | AY176148.1 | - | AY723241.1 | - | - | - | - |
| Asteraceae | Asteroideae | Anthemideae | *Matricaria* | *Matricaria matricarioides* | NCBI | MH537768.1 | AF151481.1 | EU334482.1 | EU547791.1 | MN204756.1 | - | - |
| Asteraceae | Asteroideae | Anthemideae | *Matricaria* | *Matricaria recutita* | NCBI | MG740676.1 | JN894233.1 | - | EU547790.1 | MK090287.1 | - | - |
| Asteraceae | Cichorioideae | Cichorieae | *Melanoseris* | *Melanoseris atropurpurea* | NCBI | KF485635.1 | KX526562.1 | KX526890.1 | HQ436157.1 | - | - | - |
| Asteraceae | Cichorioideae | Cichorieae | *Melanoseris* | *Melanoseris bracteata* | NCBI | LT722042.1 | - | - | LT722131.1 | - | - | - |
| Asteraceae | Cichorioideae | Cichorieae | *Melanoseris* | *Melanoseris cyanea* | NCBI | KF485623.1 | KF195999.1 | - | KF196131.1 | JQ933267.1 | - | - |
| Asteraceae | Cichorioideae | Cichorieae | *Melanoseris* | *Melanoseris graciliflora* | NCBI | KF485646.1 | KX526563.1 | KX526873.1 | LT722136.1 | - | KF732161.1 | - |
| Asteraceae | Cichorioideae | Cichorieae | *Melanoseris* | *Melanoseris henryi* | NCBI | KU746878.1 | - | - | - | - | - | - |
| Asteraceae | Cichorioideae | Cichorieae | *Melanoseris* | *Melanoseris hirsuta* | NCBI | HQ172901.1 | - | - | LS991546.1 | MG224202.1 | LS991568.1 | - |
| Asteraceae | Cichorioideae | Cichorieae | *Melanoseris* | *Melanoseris leptantha* | NCBI | KF739607.1 | - | - | - | - | - | - |
| Asteraceae | Cichorioideae | Cichorieae | *Melanoseris* | *Melanoseris lessertiana* | NCBI | KF485606.1 | - | - | KF485861.1 | - | - | - |
| Asteraceae | Cichorioideae | Cichorieae | *Melanoseris* | *Melanoseris likiangensis* | NCBI | KF485632.1 | KF196000.1 | - | KF196132.1 | KF196044.1 | - | - |
| Asteraceae | Cichorioideae | Cichorieae | *Melanoseris* | *Melanoseris macrantha* | NCBI | KF485610.1 | - | - | KF485865.1 | - | - | - |
| Asteraceae | Cichorioideae | Cichorieae | *Melanoseris* | *Melanoseris macrorhiza* | NCBI | KF485609.1 | - | - | KF485863.1 | - | - | - |
| Asteraceae | Cichorioideae | Cichorieae | *Melanoseris* | *Melanoseris monocephala* | NCBI | LT721950.1 | - | - | - | - | - | - |
| Asteraceae | Cichorioideae | Cichorieae | *Melanoseris* | *Melanoseris oligolepis* | NCBI | KF485558.1 | - | - | KF485813.1 | - | - | - |
| Asteraceae | Cichorioideae | Cichorieae | *Melanoseris* | *Melanoseris qinghaica* | NCBI | KF485613.1 | - | - | KF485868.1 | - | - | - |
| Asteraceae | Cichorioideae | Cichorieae | *Melanoseris* | *Melanoseris rhombiformis* | NCBI | KF732072.1 | - | - | - | - | KF732185.1 | - |
| Asteraceae | Cichorioideae | Cichorieae | *Melanoseris* | *Melanoseris taliensis* | NCBI | KF154386.1 | KF196001.1 | - | KF196133.1 | KF196045.1 | - | - |
| Asteraceae | Cichorioideae | Cichorieae | *Melanoseris* | *Melanoseris tenuis* | NCBI | KF485641.1 | - | - | KF485896.1 | - | - | - |
| Asteraceae | Cichorioideae | Cichorieae | *Melanoseris* | *Melanoseris triflora* | NCBI | KF485562.1 | EU046564.1 | - | KF485817.1 | - | - | - |
| Asteraceae | Cichorioideae | Cichorieae | *Melanoseris* | *Melanoseris violifolia* | NCBI | KF485612.1 | - | - | LT722139.1 | - | - | - |
| Asteraceae | Cichorioideae | Cichorieae | *Melanoseris* | *Melanoseris yunnanensis* | NCBI | KF485616.1 | - | - | KF485871.1 | - | - | - |
| Asteraceae | Asteroideae | Eupatorieae | *Mikania* | *Mikania cordata* | NCBI | AF540013.1 | - | KX526973.1 | - | KX527290.1 | - | - |
| Asteraceae | Asteroideae | Eupatorieae | *Mikania* | *Mikania micrantha* | NCBI | KY909250.1 | MF159393.1 | - | MF143645.1 | JQ933409.1 | KY909179.1 | - |
| Asteraceae | Asteroideae | Astereae | *Miyamayomena* | *Miyamayomena simplex* | NCBI | JN543733.1 | - | - | - | - | - | - |
| Asteraceae | Asteroideae | Astereae | *Myriactis* | *Myriactis delavayi* | NCBI | MH117613.1 | MH116720.1 | - | MH117171.1 | MH116262.1 | - | - |
| Asteraceae | Asteroideae | Astereae | *Myriactis* | *Myriactis humilis* | NCBI | AF046959.1 | - | - | - | - | - | - |
| Asteraceae | Asteroideae | Astereae | *Myriactis* | *Myriactis nepalensis* | NCBI | MH117614.1 | MK435737.1 | - | MH117172.1 | JQ933415.1 | - | - |
| Asteraceae | Asteroideae | Astereae | *Myriactis* | *Myriactis wallichii* | NCBI | LC027399.1 | - | - | - | - | - | - |
| Asteraceae | Asteroideae | Astereae | *Myriactis* | *Myriactis wightii* | NCBI | MH117616.1 | MH116722.1 | - | MH117174.1 | MH116266.1 | - | - |
| Asteraceae | Pertyoideae | Pertyeae | *Myripnois* | *Myripnois dioica* | NCBI | KF989607.1 | KF989916.1 | AF233846.1 | MW380893.1 | KP088698.1 | - | - |
| Asteraceae | Cichorioideae | Cichorieae | *Nabalus* | *Nabalus tatarinowii* | NCBI | FJ980337.1 | MK435738.1 | - | KF196115.1 | KF196027.1 | KF732150.1 | - |
| Asteraceae | Asteroideae | Astereae | *Nannoglottis* | *Nannoglottis carpesioides* | NCBI | AY017161.1 | - | - | - | - | - | - |
| Asteraceae | Asteroideae | Astereae | *Nannoglottis* | *Nannoglottis delavayi* | NCBI | AY017167.1 | - | - | - | - | - | - |
| Asteraceae | Asteroideae | Astereae | *Nannoglottis* | *Nannoglottis gynura* | NCBI | AY017162.1 | KX526565.1 | AY017151.1 | - | KX527013.1 | - | - |
| Asteraceae | Asteroideae | Astereae | *Nannoglottis* | *Nannoglottis latisquama* | NCBI | AY017164.1 | - | - | - | - | - | - |
| Asteraceae | Asteroideae | Astereae | *Nannoglottis* | *Nannoglottis macrocarpa* | NCBI | AY017166.1 | KX526567.1 | KX526906.1 | - | KX527007.1 | - | - |
| Asteraceae | Asteroideae | Astereae | *Nannoglottis* | *Nannoglottis ravida* | NCBI | AY017160.1 | - | AY017150.1 | - | - | - | - |
| Asteraceae | Asteroideae | Astereae | *Nannoglottis* | *Nannoglottis souliei* | NCBI | AY017163.1 | - | - | - | - | - | - |
| Asteraceae | Asteroideae | Astereae | *Nannoglottis* | *Nannoglottis yunnanensis* | NCBI | AY017165.1 | - | - | - | - | - | - |
| Asteraceae | Asteroideae | Senecioneae | *Nemosenecio* | *Nemosenecio formosanus* | NCBI | KU696044.1 | - | - | - | - | KU750261.1 | KY970403.1 |
| Asteraceae | Asteroideae | Senecioneae | *Nemosenecio* | *Nemosenecio incisifolius* | NCBI | KU696045.1 | KX526568.1 | KX526902.1 | - | KX527017.1 | KU750262.1 | - |
| Asteraceae | Asteroideae | Senecioneae | *Nemosenecio* | *Nemosenecio yunnanensis* | NCBI | KU696047.1 | - | - | - | - | KU750263.1 | KY970559.1 |
| Asteraceae | Asteroideae | Astereae | *Neobrachyactis* | *Neobrachyactis roylei* | NCBI | LC027400.1 | - | - | - | - | - | - |
| Asteraceae | Asteroideae | Anthemideae | *Neopallasia* | *Neopallasia pectinata* | NCBI | EF577316.1 | - | EU334484.1 | - | - | - | - |
| Asteraceae | Cichorioideae | Cichorieae | *Notoseris* | *Notoseris macilenta* | NCBI | LT722038.1 | KF196005.1 | - | KF196137.1 | KF196049.1 | - | - |
| Asteraceae | Cichorioideae | Cichorieae | *Notoseris* | *Notoseris scandens* | NCBI | KF485587.1 | - | - | LT722087.1 | - | - | - |
| Asteraceae | Cichorioideae | Cichorieae | *Notoseris* | *Notoseris triflora* | NCBI | KF485596.1 | KF196002.1 | - | KF196134.1 | KF196046.1 | - | - |
| Asteraceae | Cichorioideae | Cichorieae | *Notoseris* | *Notoseris yakoensis* | NCBI | KF485590.1 | - | - | KF485845.1 | - | - | - |
| Asteraceae | Wunderlichioideae | Hyalideae | *Nouelia* | *Nouelia insignis* | NCBI | MN457831.1 | EU385378.1 | EU385187.1 | - | EU385000.1 | - | - |
| Asteraceae | Carduoideae | Cardueae | *Olgaea* | *Olgaea leucophylla* | NCBI | FJ007876.1 | - | - | - | - | - | - |
| Asteraceae | Carduoideae | Cardueae | *Olgaea* | *Olgaea pectinata* | NCBI | AY826305.1 | AY785108.1 | KC589985.1 | - | KC589870.1 | - | - |
| Asteraceae | Carduoideae | Cardueae | *Oligochaeta* | *Oligochaeta minima* | NCBI | AY826307.1 | - | - | - | - | - | - |
| Asteraceae | Carduoideae | Cardueae | *Onopordum* | *Onopordum acanthium* | NCBI | MH808178.1 | KC969528.1 | - | AY914847.1 | KM360904.1 | - | - |
| Asteraceae | Asteroideae | Anthemideae | *Opisthopappus* | *Opisthopappus taihangensis* | NCBI | EF577318.1 | - | EU334485.1 | - | - | - | - |
| Asteraceae | Cichorioideae | Cichorieae | *Paraprenanthes* | *Paraprenanthes diversifolia* | NCBI | KF739611.1 | KF196007.1 | - | KF196139.1 | KF196051.1 | - | - |
| Asteraceae | Cichorioideae | Cichorieae | *Paraprenanthes* | *Paraprenanthes longiloba* | NCBI | KF485577.1 | - | - | KF485832.1 | - | - | - |
| Asteraceae | Cichorioideae | Cichorieae | *Paraprenanthes* | *Paraprenanthes melanantha* | NCBI | KF485582.1 | KF196006.1 | - | KF196138.1 | KF196050.1 | - | - |
| Asteraceae | Cichorioideae | Cichorieae | *Paraprenanthes* | *Paraprenanthes meridionalis* | NCBI | KF485571.1 | - | - | KF485826.1 | - | - | - |
| Asteraceae | Cichorioideae | Cichorieae | *Paraprenanthes* | *Paraprenanthes oligolepis* | NCBI | KF485558.1 | - | - | KF485813.1 | - | - | - |
| Asteraceae | Cichorioideae | Cichorieae | *Paraprenanthes* | *Paraprenanthes prenanthoides* | NCBI | KF485570.1 | KX526574.1 | KX526898.1 | KF485825.1 | KX526998.1 | - | - |
| Asteraceae | Cichorioideae | Cichorieae | *Paraprenanthes* | *Paraprenanthes sororia* | NCBI | MH808179.1 | KF196008.1 | - | KF196140.1 | KF196052.1 | - | - |
| Asteraceae | Cichorioideae | Cichorieae | *Paraprenanthes* | *Paraprenanthes triflora* | NCBI | KF485562.1 | EU046564.1 | - | KF485817.1 | - | - | - |
| Asteraceae | Cichorioideae | Cichorieae | *Paraprenanthes* | *Paraprenanthes umbrosa* | NCBI | KF485565.1 | - | - | KF485820.1 | - | - | - |
| Asteraceae | Cichorioideae | Cichorieae | *Paraprenanthes* | *Paraprenanthes wilsonii* | NCBI | KF485585.1 | KX526572.1 | KX526901.1 | KF485841.1 | KX527001.1 | - | - |
| Asteraceae | Cichorioideae | Cichorieae | *Paraprenanthes* | *Paraprenanthes yunnanensis* | NCBI | LT722024.1 | - | - | GU109330.1 | - | - | - |
| Asteraceae | Asteroideae | Senecioneae | *Parasenecio* | *Parasenecio ainsliaeiflorus* | NCBI | KY979030.1 | - | - | - | - | KU750264.1 | KY970387.1 |
| Asteraceae | Asteroideae | Senecioneae | *Parasenecio* | *Parasenecio albus* | NCBI | KY979025.1 | - | - | - | - | - | KY970380.1 |
| Asteraceae | Asteroideae | Senecioneae | *Parasenecio* | *Parasenecio ambiguus* | NCBI | KY979056.1 | - | - | - | - | - | KY970421.1 |
| Asteraceae | Asteroideae | Senecioneae | *Parasenecio* | *Parasenecio auriculatus* | NCBI | KY979053.1 | - | - | - | - | - | KY970418.1 |
| Asteraceae | Asteroideae | Senecioneae | *Parasenecio* | *Parasenecio bulbiferoides* | NCBI | KY979031.1 | - | - | - | - | - | KY970388.1 |
| Asteraceae | Asteroideae | Senecioneae | *Parasenecio* | *Parasenecio cyclotus* | NCBI | KY979028.1 | - | AY723251.1 | - | - | - | KY970385.1 |
| Asteraceae | Asteroideae | Senecioneae | *Parasenecio* | *Parasenecio dasythyrsus* | NCBI | KY979073.1 | - | - | - | - | - | KY970451.1 |
| Asteraceae | Asteroideae | Senecioneae | *Parasenecio* | *Parasenecio delphiniifolius* | NCBI | EF538281.1 | - | - | - | - | - | KY970509.1 |
| Asteraceae | Asteroideae | Senecioneae | *Parasenecio* | *Parasenecio deltophyllus* | NCBI | KU696051.1 | - | AY723248.1 | - | - | KU750265.1 | KY970406.1 |
| Asteraceae | Asteroideae | Senecioneae | *Parasenecio* | *Parasenecio firmus* | NCBI | KT119542.1 | - | - | - | - | KU750266.1 | KY970381.1 |
| Asteraceae | Asteroideae | Senecioneae | *Parasenecio* | *Parasenecio forrestii* | NCBI | KY979029.1 | - | - | - | - | KU750267.1 | KY970386.1 |
| Asteraceae | Asteroideae | Senecioneae | *Parasenecio* | *Parasenecio gansuensis* | NCBI | KY979077.1 | - | - | - | - | - | KY970458.1 |
| Asteraceae | Asteroideae | Senecioneae | *Parasenecio* | *Parasenecio hastatus* | NCBI | KY979052.1 | LC628098.1 | - | LC628119.1 | LC627475.1 | - | KY970417.1 |
| Asteraceae | Asteroideae | Senecioneae | *Parasenecio* | *Parasenecio hastiformis* | NCBI | AY723278.1 | - | AY723252.1 | - | - | - | - |
| Asteraceae | Asteroideae | Senecioneae | *Parasenecio* | *Parasenecio hwangshanicus* | NCBI | KY979057.1 | - | - | - | - | - | KY970422.1 |
| Asteraceae | Asteroideae | Senecioneae | *Parasenecio* | *Parasenecio ianthophyllus* | NCBI | KY979071.1 | - | - | - | - | - | KY970447.1 |
| Asteraceae | Asteroideae | Senecioneae | *Parasenecio* | *Parasenecio koualapensis* | NCBI | KY979045.1 | - | - | - | - | KU750268.1 | KY970408.1 |
| Asteraceae | Asteroideae | Senecioneae | *Parasenecio* | *Parasenecio latipes* | NCBI | MH117634.1 | KX526576.1 | KX526917.1 | MH117193.1 | KX527004.1 | - | KY970466.1 |
| Asteraceae | Asteroideae | Senecioneae | *Parasenecio* | *Parasenecio lidjiangensis* | NCBI | KY979043.1 | KX526577.1 | KX526894.1 | - | KX526995.1 | - | KY970404.1 |
| Asteraceae | Asteroideae | Senecioneae | *Parasenecio* | *Parasenecio longispicus* | NCBI | KY979072.1 | - | - | - | - | KU750269.1 | KY970448.1 |
| Asteraceae | Asteroideae | Senecioneae | *Parasenecio* | *Parasenecio maowenensis* | NCBI | AY723275.1 | - | AY723249.1 | - | - | - | - |
| Asteraceae | Asteroideae | Senecioneae | *Parasenecio* | *Parasenecio otopteryx* | NCBI | KY979054.1 | - | - | - | - | - | KY970419.1 |
| Asteraceae | Asteroideae | Senecioneae | *Parasenecio* | *Parasenecio palmatisectus* | NCBI | MH808182.1 | KX526578.1 | KX526878.1 | - | KX527015.1 | KU750270.1 | KY970465.1 |
| Asteraceae | Asteroideae | Senecioneae | *Parasenecio* | *Parasenecio phyllolepis* | NCBI | KU696060.1 | - | - | - | - | KU750271.1 | KY970464.1 |
| Asteraceae | Asteroideae | Senecioneae | *Parasenecio* | *Parasenecio pilgerianus* | NCBI | MH710824.1 | MH714256.1 | - | - | MH658329.1 | - | KY970423.1 |
| Asteraceae | Asteroideae | Senecioneae | *Parasenecio* | *Parasenecio profundorum* | NCBI | KU696061.1 | - | - | - | - | KU750272.1 | KY970410.1 |
| Asteraceae | Asteroideae | Senecioneae | *Parasenecio* | *Parasenecio quinquelobus* | NCBI | KY979046.1 | KX526579.1 | KX526931.1 | - | KX527018.1 | - | KY970409.1 |
| Asteraceae | Asteroideae | Senecioneae | *Parasenecio* | *Parasenecio roborowskii* | NCBI | MH711541.1 | MH660091.1 | - | - | MK749925.1 | - | KY970452.1 |
| Asteraceae | Asteroideae | Senecioneae | *Parasenecio* | *Parasenecio rubescens* | NCBI | KY979078.1 | - | - | - | - | - | KY970462.1 |
| Asteraceae | Asteroideae | Senecioneae | *Parasenecio* | *Parasenecio rufipilis* | NCBI | KU696062.1 | - | - | - | - | KU750273.1 | KY970461.1 |
| Asteraceae | Asteroideae | Senecioneae | *Parasenecio* | *Parasenecio sinicus* | NCBI | KY979055.1 | - | - | - | - | - | KY970420.1 |
| Asteraceae | Asteroideae | Senecioneae | *Parasenecio* | *Parasenecio taliensis* | NCBI | KY979076.1 | - | AY723250.1 | - | - | KU750275.1 | KY970457.1 |
| Asteraceae | Asteroideae | Senecioneae | *Parasenecio* | *Parasenecio tripteris* | NCBI | KY979075.1 | - | - | - | - | - | KY970454.1 |
| Asteraceae | Asteroideae | Senecioneae | *Parasenecio* | *Parasenecio tsinlingensis* | NCBI | KY979079.1 | - | - | - | - | - | KY970463.1 |
| Asteraceae | Asteroideae | Senecioneae | *Parasenecio* | *Parasenecio vespertilio* | NCBI | KU696065.1 | - | - | - | - | KU750276.1 | KY970377.1 |
| Asteraceae | Cichorioideae | Cichorieae | *Parasyncalathium* | *Parasyncalathium souliei* | NCBI | LT721955.1 | JF956608.1 | - | HQ436173.1 | JF944577.1 | KR733633.1 | - |
| Asteraceae | Asteroideae | Heliantheae | *Parthenium* | *Parthenium hysterophorus* | NCBI | MW466703.1 | MH017987.1 | AF384759.2 | MH017888.1 | AY215155.1 | FJ041061.1 | EU440122.1 |
| Asteraceae | Asteroideae | Tageteae | *Pectis* | *Pectis prostrata* | NCBI | KJ524982.1 | MT214853.1 | AF405278.1 | - | KJ773729.1 | - | - |
| Asteraceae | Asteroideae | Inuleae | *Pentanema* | *Pentanema cernuum* | NCBI | EF210975.1 | - | - | - | - | - | - |
| Asteraceae | Asteroideae | Inuleae | *Pentanema* | *Pentanema indicum* | NCBI | KY696329.1 | - | FM208980.1 | EF211001.1 | - | - | - |
| Asteraceae | Asteroideae | Senecioneae | *Pericallis* | *Pericallis hybrida* | NCBI | - | KX783744.1 | - | - | KX783956.1 | - | - |
| Asteraceae | Pertyoideae | Pertyeae | *Pertya* | *Pertya scandens* | NCBI | LC605681.1 | EU385386.1 | EU385195.1 | LC605692.1 | EU385008.1 | - | - |
| Asteraceae | Pertyoideae | Pertyeae | *Pertya* | *Pertya sinensis* | NCBI | MH711279.1 | MH659741.1 | - | - | MH658261.1 | - | - |
| Asteraceae | Asteroideae | Senecioneae | *Petasites* | *Petasites formosanus* | NCBI | KU570781.1 | - | - | - | - | - | - |
| Asteraceae | Asteroideae | Senecioneae | *Petasites* | *Petasites japonicus* | NCBI | KY387918.1 | MH659524.1 | AY723240.1 | GQ435096.1 | GQ436454.1 | KU750277.1 | - |
| Asteraceae | Asteroideae | Senecioneae | *Petasites* | *Petasites rubellus* | NCBI | KU570796.1 | - | - | - | - | - | - |
| Asteraceae | Asteroideae | Senecioneae | *Petasites* | *Petasites tricholobus* | NCBI | MH808185.1 | KT149956.1 | - | - | JQ933441.1 | - | KY970391.1 |
| Asteraceae | Asteroideae | Gnaphalieae | *Phagnalon* | *Phagnalon niveum* | NCBI | HM246028.1 | - | - | - | JQ933443.1 | - | - |
| Asteraceae | Cichorioideae | Cichorieae | *Picris* | *Picris hieracioides* | NCBI | MH711178.1 | MH659153.1 | - | HE966737.1 | KF196021.1 | MT234736.1 | - |
| Asteraceae | Cichorioideae | Cichorieae | *Picris* | *Picris japonica* | NCBI | KC121976.1 | MF158671.1 | - | - | MF158757.1 | - | - |
| Asteraceae | Cichorioideae | Cichorieae | *Picris* | *Picris junnanensis* | NCBI | KX643613.1 | - | - | - | - | - | - |
| Asteraceae | Cichorioideae | Cichorieae | *Picris* | *Picris morrisonensis* | NCBI | EF107658.1 | - | - | - | - | - | - |
| Asteraceae | Cichorioideae | Cichorieae | *Picris* | *Picris nuristanica* | NCBI | KC121979.1 | DQ451737.1 | - | - | - | - | - |
| Asteraceae | Cichorioideae | Cichorieae | *Pilosella* | *Pilosella echioides* | NCBI | KM372112.1 | - | - | - | - | - | - |
| Asteraceae | Carduoideae | Cardueae | *Plagiobasis* | *Plagiobasis centauroides* | NCBI | AY826312.1 | - | JF754852.1 | - | - | - | - |
| Asteraceae | Asteroideae | Inuleae | *Pluchea* | *Pluchea carolinensis* | NCBI | AF437851.1 | EU385389.1 | EU385198.1 | HG963709.1 | EU385011.1 | - | - |
| Asteraceae | Asteroideae | Inuleae | *Pluchea* | *Pluchea pteropoda* | NCBI | AF437852.1 | - | - | - | - | - | - |
| Asteraceae | Asteroideae | Inuleae | *Pluchea* | *Pluchea sagittalis* | NCBI | EF108399.1 | MH070451.1 | LN607676.1 | - | MH049945.1 | - | - |
| Asteraceae | Asteroideae | Eupatorieae | *Praxelis* | *Praxelis clematidea* | NCBI | MN889429.1 | KX526581.1 | KP454975.1 | - | KX527419.1 | - | - |
| Asteraceae | Cichorioideae | Vernonieae | *Pseudelephantopus* | *Pseudelephantopus spicatus* | NCBI | HQ158410.1 | JQ586852.1 | HQ158514.1 | HG963589.1 | JQ590628.1 | - | - |
| Asteraceae | Asteroideae | Gnaphalieae | *Pseudognaphalium* | *Pseudognaphalium adnatum* | NCBI | KT865444.1 | - | - | - | - | - | - |
| Asteraceae | Asteroideae | Gnaphalieae | *Pseudognaphalium* | *Pseudognaphalium affine* | NCBI | EF108400.1 | HM989784.1 | - | MF785889.1 | MH767499.1 | - | - |
| Asteraceae | Asteroideae | Gnaphalieae | *Pseudognaphalium* | *Pseudognaphalium chrysocephalum* | NCBI | KJ459260.1 | AF151452.1 | - | KF263823.1 | - | - | - |
| Asteraceae | Asteroideae | Gnaphalieae | *Pseudognaphalium* | *Pseudognaphalium flavescens* | NCBI | KT865574.1 | - | - | - | - | - | - |
| Asteraceae | Asteroideae | Gnaphalieae | *Pseudognaphalium* | *Pseudognaphalium hypoleucum* | NCBI | EF108392.1 | MK435743.1 | - | - | MK749926.1 | - | - |
| Asteraceae | Asteroideae | Gnaphalieae | *Pseudognaphalium* | *Pseudognaphalium luteoalbum* | NCBI | AY445227.1 | HM445639.1 | HM445674.1 | - | KT626769.1 | - | - |
| Asteraceae | Asteroideae | Anthemideae | *Pseudohandelia* | *Pseudohandelia umbellifera* | NCBI | LC313949.1 | - | AF153629.1 | AB683368.1 | - | - | - |
| Asteraceae | Asteroideae | Astereae | *Psychrogeton* | *Psychrogeton nigromontanus* | NCBI | LC027414.1 | - | - | LC027433.1 | - | - | - |
| Asteraceae | Asteroideae | Inuleae | *Pterocaulon* | *Pterocaulon redolens* | NCBI | LN607558.1 | - | LN607682.1 | - | - | - | - |
| Asteraceae | Asteroideae | Inuleae | *Pulicaria* | *Pulicaria dysenterica* | NCBI | FM995395.1 | JN895636.1 | LN607688.1 | FM998693.1 | KM360946.1 | - | - |
| Asteraceae | Asteroideae | Inuleae | *Pulicaria* | *Pulicaria vulgaris* | NCBI | KJ004326.1 | MK926087.1 | FM209029.1 | FM998705.1 | MK925449.1 | - | - |
| Asteraceae | Carduoideae | Cardueae | *Rhaponticum* | *Rhaponticum carthamoides* | NCBI | MK049257.1 | - | - | - | MZ346035.1 | - | - |
| Asteraceae | Carduoideae | Cardueae | *Rhaponticum* | *Rhaponticum repens* | NCBI | EU409919.1 | AY013489.1 | JF754831.1 | - | KC589889.1 | - | - |
| Asteraceae | Carduoideae | Cardueae | *Rhaponticum* | *Rhaponticum uniflorum* | NCBI | MH711158.1 | MK435744.1 | - | GQ435113.1 | GQ436467.1 | - | - |
| Asteraceae | Asteroideae | Astereae | *Rhinactinidia* | *Rhinactinidia eremophila* | NCBI | JN543727.1 | - | - | - | - | - | - |
| Asteraceae | Asteroideae | Anthemideae | *Richteria* | *Richteria pyrethroides* | NCBI | LC313951.1 | - | - | AB683369.1 | - | - | - |
| Asteraceae | Asteroideae | Heliantheae | *Rudbeckia* | *Rudbeckia hirta* | NCBI | KX671869.1 | AY215856.1 | AF384778.3 | HQ596825.1 | KT178110.1 | KT179024.1 | - |
| Asteraceae | Asteroideae | Heliantheae | *Rudbeckia* | *Rudbeckia laciniata* | NCBI | KX671870.1 | MG225258.1 | - | KP643579.1 | KJ773848.1 | - | EU440118.1 |
| Asteraceae | Carduoideae | Cardueae | *Russowia* | *Russowia sogdiana* | NCBI | AY826320.1 | - | JF754860.1 | - | - | - | - |
| Asteraceae | Carduoideae | Cardueae | *Saussurea* | *Saussurea acromelaena* | NCBI | MH711376.1 | MH659864.1 | - | - | MH658386.1 | - | - |
| Asteraceae | Carduoideae | Cardueae | *Saussurea* | *Saussurea acuminata* | NCBI | AY366331.1 | - | - | - | - | - | - |
| Asteraceae | Carduoideae | Cardueae | *Saussurea* | *Saussurea alata* | NCBI | EF420929.1 | - | - | EF420894.1 | - | - | - |
| Asteraceae | Carduoideae | Cardueae | *Saussurea* | *Saussurea alpina* | NCBI | AB118124.1 | MK925743.1 | KC589996.1 | AY914848.1 | KC589890.1 | - | - |
| Asteraceae | Carduoideae | Cardueae | *Saussurea* | *Saussurea amurensis* | NCBI | AB254645.1 | - | - | - | - | - | - |
| Asteraceae | Carduoideae | Cardueae | *Saussurea* | *Saussurea aster* | NCBI | AB118110.1 | AB118141.1 | - | - | - | - | - |
| Asteraceae | Carduoideae | Cardueae | *Saussurea* | *Saussurea bhutkesh* | NCBI | AB254664.1 | - | - | - | - | - | - |
| Asteraceae | Carduoideae | Cardueae | *Saussurea* | *Saussurea bracteata* | NCBI | MK225642.1 | MH070634.1 | AY466416.1 | MH070760.1 | MH070887.1 | MH071013.1 | - |
| Asteraceae | Carduoideae | Cardueae | *Saussurea* | *Saussurea brunneopilosa* | NCBI | EF420942.1 | - | - | EF420906.1 | - | - | - |
| Asteraceae | Carduoideae | Cardueae | *Saussurea* | *Saussurea candolleana* | NCBI | MK225639.1 | - | - | HQ690925.1 | - | - | - |
| Asteraceae | Carduoideae | Cardueae | *Saussurea* | *Saussurea ceterach* | NCBI | AY366318.1 | - | - | - | - | - | - |
| Asteraceae | Carduoideae | Cardueae | *Saussurea* | *Saussurea conaensis* | NCBI | AB254651.1 | - | - | - | - | - | - |
| Asteraceae | Carduoideae | Cardueae | *Saussurea* | *Saussurea coriacea* | NCBI | AY366316.1 | - | AY466404.1 | - | - | - | - |
| Asteraceae | Carduoideae | Cardueae | *Saussurea* | *Saussurea daurica* | NCBI | AY366347.1 | - | AY466413.1 | - | - | - | - |
| Asteraceae | Carduoideae | Cardueae | *Saussurea* | *Saussurea delavayi* | NCBI | AB254648.1 | - | - | EF420887.1 | - | - | - |
| Asteraceae | Carduoideae | Cardueae | *Saussurea* | *Saussurea depsangensis* | NCBI | AY366325.1 | - | AY466420.1 | - | - | - | - |
| Asteraceae | Carduoideae | Cardueae | *Saussurea* | *Saussurea dzeurensis* | NCBI | EF420950.1 | - | - | EF420912.1 | - | - | - |
| Asteraceae | Carduoideae | Cardueae | *Saussurea* | *Saussurea elegans* | NCBI | AB118115.1 | AB118144.1 | - | - | MK954375.1 | - | - |
| Asteraceae | Carduoideae | Cardueae | *Saussurea* | *Saussurea erubescens* | NCBI | MH003727.1 | MH070639.1 | AY466415.1 | MH070765.1 | MH070892.1 | MH071018.1 | - |
| Asteraceae | Carduoideae | Cardueae | *Saussurea* | *Saussurea fastuosa* | NCBI | DQ874337.1 | - | - | DQ874339.1 | - | - | - |
| Asteraceae | Carduoideae | Cardueae | *Saussurea* | *Saussurea georgei* | NCBI | MH293161.1 | MH293234.1 | - | - | - | - | - |
| Asteraceae | Carduoideae | Cardueae | *Saussurea* | *Saussurea glacialis* | NCBI | AB118121.1 | AB118146.1 | - | - | - | - | - |
| Asteraceae | Carduoideae | Cardueae | *Saussurea* | *Saussurea globosa* | NCBI | MH003735.1 | MH070646.1 | AY466426.1 | MH070772.1 | MH070899.1 | MH071025.1 | - |
| Asteraceae | Carduoideae | Cardueae | *Saussurea* | *Saussurea gnaphalodes* | NCBI | AB118122.2 | AB118147.1 | - | EF420884.1 | - | - | - |
| Asteraceae | Carduoideae | Cardueae | *Saussurea* | *Saussurea graminifolia* | NCBI | AB254654.1 | - | - | - | - | - | - |
| Asteraceae | Carduoideae | Cardueae | *Saussurea* | *Saussurea grandiceps* | NCBI | EF420921.1 | - | - | EF420885.1 | - | - | - |
| Asteraceae | Carduoideae | Cardueae | *Saussurea* | *Saussurea grandifolia* | NCBI | JX274506.1 | - | - | - | - | - | - |
| Asteraceae | Carduoideae | Cardueae | *Saussurea* | *Saussurea gymnocephala* | NCBI | AB254671.1 | MF680728.1 | AY466411.1 | MF680768.1 | - | MF680808.1 | - |
| Asteraceae | Carduoideae | Cardueae | *Saussurea* | *Saussurea haoi* | NCBI | AY366326.1 | - | AY466421.1 | - | - | - | - |
| Asteraceae | Carduoideae | Cardueae | *Saussurea* | *Saussurea hieracioides* | NCBI | AB254655.1 | KT280256.1 | - | - | KT280153.1 | - | - |
| Asteraceae | Carduoideae | Cardueae | *Saussurea* | *Saussurea hookeri* | NCBI | AB118118.1 | - | AY466407.1 | - | - | - | - |
| Asteraceae | Carduoideae | Cardueae | *Saussurea* | *Saussurea hwangshanensis* | NCBI | AB254657.1 | - | - | - | - | - | - |
| Asteraceae | Carduoideae | Cardueae | *Saussurea* | *Saussurea hypsipeta* | NCBI | AF257788.1 | - | - | EF420883.1 | - | - | - |
| Asteraceae | Carduoideae | Cardueae | *Saussurea* | *Saussurea involucrata* | NCBI | MH003747.1 | MH070658.1 | AY466424.1 | MH070783.1 | GQ436481.1 | MH071037.1 | - |
| Asteraceae | Carduoideae | Cardueae | *Saussurea* | *Saussurea iodostegia* | NCBI | MH711512.1 | MH660056.1 | - | MH070792.1 | MH070919.1 | MH071045.1 | - |
| Asteraceae | Carduoideae | Cardueae | *Saussurea* | *Saussurea japonica* | NCBI | MH712612.1 | MH660020.1 | - | - | MH713960.1 | - | - |
| Asteraceae | Carduoideae | Cardueae | *Saussurea* | *Saussurea katochaete* | NCBI | AB118120.1 | MZ152232.1 | AY466423.1 | EF420900.1 | - | - | - |
| Asteraceae | Carduoideae | Cardueae | *Saussurea* | *Saussurea kingii* | NCBI | EF420915.1 | - | AY466408.1 | EF420878.1 | - | - | - |
| Asteraceae | Carduoideae | Cardueae | *Saussurea* | *Saussurea kungii* | NCBI | EF420946.1 | - | - | - | - | - | - |
| Asteraceae | Carduoideae | Cardueae | *Saussurea* | *Saussurea laniceps* | NCBI | AB254659.1 | - | - | - | - | - | - |
| Asteraceae | Carduoideae | Cardueae | *Saussurea* | *Saussurea leiocarpa* | NCBI | MH293176.1 | - | - | - | MH293315.1 | - | - |
| Asteraceae | Carduoideae | Cardueae | *Saussurea* | *Saussurea leontodontoides* | NCBI | AB254660.1 | JQ303115.1 | JQ303119.1 | - | JQ303117.1 | - | - |
| Asteraceae | Carduoideae | Cardueae | *Saussurea* | *Saussurea leucoma* | NCBI | AB254661.1 | - | - | - | - | - | - |
| Asteraceae | Carduoideae | Cardueae | *Saussurea* | *Saussurea luae* | NCBI | MH293162.1 | MH293235.1 | - | MH070794.1 | MH293305.1 | MH071047.1 | - |
| Asteraceae | Carduoideae | Cardueae | *Saussurea* | *Saussurea manshurica* | NCBI | KC969558.1 | KC969533.1 | - | - | - | - | - |
| Asteraceae | Carduoideae | Cardueae | *Saussurea* | *Saussurea maximowiczii* | NCBI | AY826324.1 | AY373682.1 | JF754861.1 | - | - | - | - |
| Asteraceae | Carduoideae | Cardueae | *Saussurea* | *Saussurea medusa* | NCBI | AB118123.1 | - | AY466414.1 | EF420882.1 | - | - | - |
| Asteraceae | Carduoideae | Cardueae | *Saussurea* | *Saussurea muliensis* | NCBI | AB254665.1 | - | - | - | - | - | - |
| Asteraceae | Carduoideae | Cardueae | *Saussurea* | *Saussurea mutabilis* | NCBI | MH711521.1 | MN273602.1 | - | - | MN185086.1 | - | - |
| Asteraceae | Carduoideae | Cardueae | *Saussurea* | *Saussurea nematolepis* | NCBI | AB254666.1 | - | AY466428.1 | EF420896.1 | - | - | - |
| Asteraceae | Carduoideae | Cardueae | *Saussurea* | *Saussurea neoserrata* | NCBI | AB254668.1 | - | - | - | - | - | - |
| Asteraceae | Carduoideae | Cardueae | *Saussurea* | *Saussurea nidularis* | NCBI | AB254675.1 | MH070696.1 | AY466422.1 | MH070823.1 | MH070949.1 | MH071075.1 | - |
| Asteraceae | Carduoideae | Cardueae | *Saussurea* | *Saussurea nigrescens* | NCBI | MH003767.1 | MK435745.1 | - | MH070801.1 | MN185087.1 | MH071054.1 | - |
| Asteraceae | Carduoideae | Cardueae | *Saussurea* | *Saussurea nishiokae* | NCBI | AB254670.1 | - | - | - | - | - | - |
| Asteraceae | Carduoideae | Cardueae | *Saussurea* | *Saussurea obvallata* | NCBI | AB254671.1 | MF680728.1 | AY466411.1 | MF680768.1 | - | MF680808.1 | - |
| Asteraceae | Carduoideae | Cardueae | *Saussurea* | *Saussurea ochrochlaena* | NCBI | MH293163.1 | MH293236.1 | - | - | - | - | - |
| Asteraceae | Carduoideae | Cardueae | *Saussurea* | *Saussurea orgaadayi* | NCBI | ON394567.1 | MH070683.1 | - | MT624065.1 | MT624053.1 | MH071062.1 | - |
| Asteraceae | Carduoideae | Cardueae | *Saussurea* | *Saussurea pachyneura* | NCBI | AB254672.1 | - | - | EF420907.1 | KT280155.1 | - | - |
| Asteraceae | Carduoideae | Cardueae | *Saussurea* | *Saussurea parviflora* | NCBI | JX274503.1 | - | - | - | - | - | - |
| Asteraceae | Carduoideae | Cardueae | *Saussurea* | *Saussurea paxiana* | NCBI | MH293164.1 | MH293237.1 | - | - | - | - | - |
| Asteraceae | Carduoideae | Cardueae | *Saussurea* | *Saussurea peduncularis* | NCBI | MH117745.1 | MH116849.1 | - | MH117306.1 | MH116400.1 | - | - |
| Asteraceae | Carduoideae | Cardueae | *Saussurea* | *Saussurea phaeantha* | NCBI | MG386450.1 | MH070690.1 | - | MG386447.1 | MH070943.1 | MH071069.1 | - |
| Asteraceae | Carduoideae | Cardueae | *Saussurea* | *Saussurea pinetorum* | NCBI | AB254674.1 | - | - | - | - | - | - |
| Asteraceae | Carduoideae | Cardueae | *Saussurea* | *Saussurea polypodioides* | NCBI | AY366317.1 | - | AY466406.1 | - | - | - | - |
| Asteraceae | Carduoideae | Cardueae | *Saussurea* | *Saussurea poochlamys* | NCBI | EF420932.1 | - | - | - | - | - | - |
| Asteraceae | Carduoideae | Cardueae | *Saussurea* | *Saussurea populifolia* | NCBI | MH703272.1 | MH659837.1 | - | - | MH658359.1 | - | - |
| Asteraceae | Carduoideae | Cardueae | *Saussurea* | *Saussurea przewalskii* | NCBI | AB118127.2 | MK435747.1 | - | EF420908.1 | MN185088.1 | - | - |
| Asteraceae | Carduoideae | Cardueae | *Saussurea* | *Saussurea pubifolia* | NCBI | MH003793.1 | MH070700.1 | - | MH070827.1 | MH070953.1 | MH071079.1 | - |
| Asteraceae | Carduoideae | Cardueae | *Saussurea* | *Saussurea pulchella* | NCBI | JX274505.1 | AB118150.1 | - | - | - | - | - |
| Asteraceae | Carduoideae | Cardueae | *Saussurea* | *Saussurea pulvinata* | NCBI | EF420937.1 | - | - | EF420902.1 | - | - | - |
| Asteraceae | Carduoideae | Cardueae | *Saussurea* | *Saussurea pumila* | NCBI | AB254676.1 | - | - | - | - | - | - |
| Asteraceae | Carduoideae | Cardueae | *Saussurea* | *Saussurea quercifolia* | NCBI | MH293165.1 | MH293238.1 | - | - | MH293306.1 | - | - |
| Asteraceae | Carduoideae | Cardueae | *Saussurea* | *Saussurea rhytidocarpa* | NCBI | EF420944.1 | - | - | EF420909.1 | - | - | - |
| Asteraceae | Carduoideae | Cardueae | *Saussurea* | *Saussurea rockii* | NCBI | AB118128.1 | AB118151.1 | - | - | - | - | - |
| Asteraceae | Carduoideae | Cardueae | *Saussurea* | *Saussurea romuleifolia* | NCBI | AB254677.1 | - | - | - | - | - | - |
| Asteraceae | Carduoideae | Cardueae | *Saussurea* | *Saussurea salicifolia* | NCBI | EF420939.1 | - | - | EF420904.1 | - | - | - |
| Asteraceae | Carduoideae | Cardueae | *Saussurea* | *Saussurea salsa* | NCBI | AB118129.1 | - | - | EF420903.1 | - | - | - |
| Asteraceae | Carduoideae | Cardueae | *Saussurea* | *Saussurea schanginiana* | NCBI | AB254679.1 | - | - | - | - | - | - |
| Asteraceae | Carduoideae | Cardueae | *Saussurea* | *Saussurea schlagintweitii* | NCBI | EF420941.1 | - | - | - | - | - | - |
| Asteraceae | Carduoideae | Cardueae | *Saussurea* | *Saussurea semilyrata* | NCBI | AB254680.1 | - | - | - | - | - | - |
| Asteraceae | Carduoideae | Cardueae | *Saussurea* | *Saussurea simpsoniana* | NCBI | AB254681.1 | - | - | HQ690931.1 | - | - | - |
| Asteraceae | Carduoideae | Cardueae | *Saussurea* | *Saussurea sinuata* | NCBI | AB254682.1 | - | - | - | - | - | - |
| Asteraceae | Carduoideae | Cardueae | *Saussurea* | *Saussurea sobarocephala* | NCBI | EF420940.1 | - | - | EF420905.1 | - | - | - |
| Asteraceae | Carduoideae | Cardueae | *Saussurea* | *Saussurea sordida* | NCBI | AB254684.1 | - | - | - | - | - | - |
| Asteraceae | Carduoideae | Cardueae | *Saussurea* | *Saussurea spatulifolia* | NCBI | AB254685.1 | - | - | EF420901.1 | - | - | - |
| Asteraceae | Carduoideae | Cardueae | *Saussurea* | *Saussurea stella* | NCBI | AB254686.1 | KT280258.1 | AY466425.1 | EF420881.1 | KT280156.1 | - | - |
| Asteraceae | Carduoideae | Cardueae | *Saussurea* | *Saussurea stoliczkae* | NCBI | AB254687.1 | - | - | - | - | - | - |
| Asteraceae | Carduoideae | Cardueae | *Saussurea* | *Saussurea subulata* | NCBI | AB118132.1 | AB118152.1 | AY466418.1 | EF420898.1 | - | - | - |
| Asteraceae | Carduoideae | Cardueae | *Saussurea* | *Saussurea subulisquama* | NCBI | AB254688.1 | - | - | - | - | - | - |
| Asteraceae | Carduoideae | Cardueae | *Saussurea* | *Saussurea superba* | NCBI | AY366336.1 | - | - | - | - | - | - |
| Asteraceae | Carduoideae | Cardueae | *Saussurea* | *Saussurea tangutica* | NCBI | AB254695.1 | MH070713.1 | - | MH070840.1 | MH070966.1 | MH071092.1 | - |
| Asteraceae | Carduoideae | Cardueae | *Saussurea* | *Saussurea taraxacifolia* | NCBI | AB254690.1 | - | - | - | - | - | - |
| Asteraceae | Carduoideae | Cardueae | *Saussurea* | *Saussurea tatsienensis* | NCBI | AB254691.1 | - | - | - | - | - | - |
| Asteraceae | Carduoideae | Cardueae | *Saussurea* | *Saussurea thomsonii* | NCBI | AB254698.1 | - | - | EF420879.1 | - | - | - |
| Asteraceae | Carduoideae | Cardueae | *Saussurea* | *Saussurea thoroldii* | NCBI | AB254699.1 | - | AY466417.1 | EF420880.1 | - | - | - |
| Asteraceae | Carduoideae | Cardueae | *Saussurea* | *Saussurea tibetica* | NCBI | AY366315.1 | - | - | - | - | - | - |
| Asteraceae | Carduoideae | Cardueae | *Saussurea* | *Saussurea tomentosa* | NCBI | EF420947.1 | - | - | EF420910.1 | - | - | - |
| Asteraceae | Carduoideae | Cardueae | *Saussurea* | *Saussurea topkegolensis* | NCBI | AB254692.1 | - | - | - | - | - | - |
| Asteraceae | Carduoideae | Cardueae | *Saussurea* | *Saussurea uliginosa* | NCBI | EF420922.1 | - | - | - | - | - | - |
| Asteraceae | Carduoideae | Cardueae | *Saussurea* | *Saussurea umbrosa* | NCBI | AB254694.1 | - | - | - | - | - | - |
| Asteraceae | Carduoideae | Cardueae | *Saussurea* | *Saussurea uniflora* | NCBI | MH003813.1 | MH070719.1 | - | MH070846.1 | MH070972.1 | MH071098.1 | - |
| Asteraceae | Carduoideae | Cardueae | *Saussurea* | *Saussurea veitchiana* | NCBI | MH003826.1 | MH070732.1 | - | MH070859.1 | MH070985.1 | MH071104.1 | - |
| Asteraceae | Carduoideae | Cardueae | *Saussurea* | *Saussurea velutina* | NCBI | MG386451.1 | MH070737.1 | - | MG386448.1 | MH070990.1 | MH071116.1 | - |
| Asteraceae | Carduoideae | Cardueae | *Saussurea* | *Saussurea wardii* | NCBI | AB254696.1 | - | - | - | - | - | - |
| Asteraceae | Carduoideae | Cardueae | *Saussurea* | *Saussurea wellbyi* | NCBI | AB254697.1 | - | - | - | - | - | - |
| Asteraceae | Carduoideae | Cardueae | *Saussurea* | *Saussurea wettsteiniana* | NCBI | MH003835.1 | MH070741.1 | - | MH070868.1 | MH070994.1 | MH071120.1 | - |
| Asteraceae | Carduoideae | Cardueae | *Saussurea* | *Saussurea woodiana* | NCBI | EF420951.1 | - | - | EF420913.1 | - | - | - |
| Asteraceae | Carduoideae | Cardueae | *Saussurea* | *Saussurea yunnanensis* | NCBI | MT914281.1 | MT928672.1 | - | - | MT921018.1 | - | - |
| Asteraceae | Carduoideae | Cardueae | *Schischkinia* | *Schischkinia albispina* | NCBI | AY826325.1 | AY785113.1 | JF754862.1 | - | KC589892.1 | - | - |
| Asteraceae | Carduoideae | Cardueae | *Schmalhausenia* | *Schmalhausenia nidulans* | NCBI | AY826326.1 | AY373681.1 | KC589917.1 | - | KC589790.1 | - | - |
| Asteraceae | Cichorioideae | Cichorieae | *Scorzonera* | *Scorzonera albicaulis* | NCBI | MH711349.1 | MH659824.1 | - | - | MH658346.1 | - | - |
| Asteraceae | Cichorioideae | Cichorieae | *Scorzonera* | *Scorzonera austriaca* | NCBI | GU724301.1 | KT250115.1 | - | - | MN204763.1 | KC968135.1 | - |
| Asteraceae | Cichorioideae | Cichorieae | *Scorzonera* | *Scorzonera capito* | NCBI | MN307485.1 | MN695369.1 | - | - | MN695370.1 | - | - |
| Asteraceae | Cichorioideae | Cichorieae | *Scorzonera* | *Scorzonera divaricata* | NCBI | MN312200.1 | MN737473.1 | - | - | JQ933473.1 | - | - |
| Asteraceae | Cichorioideae | Cichorieae | *Scorzonera* | *Scorzonera ikonnikovii* | NCBI | MN307487.1 | MN695371.1 | - | - | MN695372.1 | - | - |
| Asteraceae | Cichorioideae | Cichorieae | *Scorzonera* | *Scorzonera parviflora* | NCBI | HM802298.1 | - | - | - | - | - | - |
| Asteraceae | Cichorioideae | Cichorieae | *Scorzonera* | *Scorzonera pseudodivaricata* | NCBI | MN296016.1 | MN695365.1 | - | - | MN695366.1 | - | - |
| Asteraceae | Cichorioideae | Cichorieae | *Scorzonera* | *Scorzonera pubescens* | NCBI | MN307900.1 | - | - | - | - | - | - |
| Asteraceae | Cichorioideae | Cichorieae | *Scorzonera* | *Scorzonera pusilla* | NCBI | MZ668653.1 | MN695373.1 | - | - | MN695374.1 | - | - |
| Asteraceae | Cichorioideae | Cichorieae | *Scorzonera* | *Scorzonera subacaulis* | NCBI | MN307484.1 | - | - | - | - | - | - |
| Asteraceae | Asteroideae | Senecioneae | *Senecio* | *Senecio acutipinnus* | NCBI | MN031267.1 | - | - | - | - | - | - |
| Asteraceae | Asteroideae | Senecioneae | *Senecio* | *Senecio analogus* | NCBI | AF459947.1 | AF459980.1 | - | AY155651.1 | - | - | - |
| Asteraceae | Asteroideae | Senecioneae | *Senecio* | *Senecio argunensis* | NCBI | AY176154.1 | - | AY723208.1 | - | - | - | - |
| Asteraceae | Asteroideae | Senecioneae | *Senecio* | *Senecio cannabifolius* | NCBI | AF459949.1 | AF460011.1 | - | AY155649.1 | LC364370.1 | - | - |
| Asteraceae | Asteroideae | Senecioneae | *Senecio* | *Senecio changii* | NCBI | KU499907.1 | - | - | - | - | - | - |
| Asteraceae | Asteroideae | Senecioneae | *Senecio* | *Senecio densiserratus* | NCBI | MH808196.1 | - | AY723210.1 | - | - | - | - |
| Asteraceae | Asteroideae | Senecioneae | *Senecio* | *Senecio graciliflorus* | NCBI | MN031270.1 | - | - | - | JQ933248.1 | - | - |
| Asteraceae | Asteroideae | Senecioneae | *Senecio* | *Senecio inaequidens* | NCBI | JN789806.1 | KT250039.1 | JN790028.1 | JN789928.1 | HE963661.1 | - | - |
| Asteraceae | Asteroideae | Senecioneae | *Senecio* | *Senecio jacobaea* | NCBI | GU818567.1 | AY554084.1 | EF537971.1 | AY155657.1 | GU817769.1 | - | - |
| Asteraceae | Asteroideae | Senecioneae | *Senecio* | *Senecio krascheninnikovii* | NCBI | AF457437.1 | - | - | - | - | - | - |
| Asteraceae | Asteroideae | Senecioneae | *Senecio* | *Senecio laetus* | NCBI | KU696069.1 | - | AY723213.1 | - | - | KU750278.1 | - |
| Asteraceae | Asteroideae | Senecioneae | *Senecio* | *Senecio lijiangensis* | NCBI | KU696070.1 | - | - | - | - | KU750279.1 | - |
| Asteraceae | Asteroideae | Senecioneae | *Senecio* | *Senecio lingianus* | NCBI | KM592505.1 | - | - | - | - | - | - |
| Asteraceae | Asteroideae | Senecioneae | *Senecio* | *Senecio nemorensis* | NCBI | MH710657.1 | MH660029.1 | AY723209.1 | EF538046.2 | MH657705.1 | - | - |
| Asteraceae | Asteroideae | Senecioneae | *Senecio* | *Senecio nodiflorus* | NCBI | MH293166.1 | MH293239.1 | - | - | MH293307.1 | - | - |
| Asteraceae | Asteroideae | Senecioneae | *Senecio* | *Senecio pseudoarnica* | NCBI | LC681520.1 | LC681513.1 | - | LC681522.1 | LC681497.1 | - | - |
| Asteraceae | Asteroideae | Senecioneae | *Senecio* | *Senecio thianschanicus* | NCBI | AY176156.1 | - | AY723207.1 | - | - | - | - |
| Asteraceae | Asteroideae | Senecioneae | *Senecio* | *Senecio tricuspis* | NCBI | KU696072.1 | - | - | - | - | KU750281.1 | - |
| Asteraceae | Asteroideae | Senecioneae | *Senecio* | *Senecio vulgaris* | NCBI | MH364395.1 | AF151509.1 | EF538017.2 | FJ493263.1 | HM850347.1 | KU750282.1 | - |
| Asteraceae | Asteroideae | Anthemideae | *Seriphidium* | *Seriphidium brevifolium* | NCBI | KC493069.1 | - | - | - | - | - | - |
| Asteraceae | Asteroideae | Anthemideae | *Seriphidium* | *Seriphidium karatavicum* | NCBI | JX051660.1 | - | - | JN862047.1 | - | - | - |
| Asteraceae | Carduoideae | Cardueae | *Serratula* | *Serratula coronata* | NCBI | MH808198.1 | AB118154.1 | JF754863.1 | - | KC589893.1 | KJ826304.1 | - |
| Asteraceae | Carduoideae | Cardueae | *Serratula* | *Serratula strangulata* | NCBI | AY914830.1 | - | - | AY914850.1 | JQ933477.1 | - | - |
| Asteraceae | Asteroideae | Astereae | *Sheareria* | *Sheareria nana* | NCBI | JN543703.1 | KX526582.1 | - | - | KX527400.1 | - | - |
| Asteraceae | Asteroideae | Millieae | *Sigesbeckia* | *Sigesbeckia glabrescens* | NCBI | MH701847.1 | - | - | GQ435103.1 | - | - | - |
| Asteraceae | Asteroideae | Millieae | *Sigesbeckia* | *Sigesbeckia orientalis* | NCBI | JN987228.1 | HM989746.1 | - | GQ435085.1 | JQ933480.1 | - | - |
| Asteraceae | Asteroideae | Millieae | *Sigesbeckia* | *Sigesbeckia pubescens* | NCBI | MH701848.1 | MK435749.1 | - | - | - | - | - |
| Asteraceae | Asteroideae | Senecioneae | *Sinacalia* | *Sinacalia caroli* | NCBI | KY979047.1 | - | - | - | - | KU750283.1 | KY970411.1 |
| Asteraceae | Asteroideae | Senecioneae | *Sinacalia* | *Sinacalia davidii* | NCBI | MH808203.1 | - | - | - | - | KU750284.1 | KY970467.1 |
| Asteraceae | Asteroideae | Senecioneae | *Sinacalia* | *Sinacalia tangutica* | NCBI | MH808204.1 | GU817553.1 | AY723243.1 | GU818472.1 | MN185089.1 | KU750285.1 | KY970395.1 |
| Asteraceae | Asteroideae | Gnaphalieae | *Sinoleontopodium* | *Sinoleontopodium lingianum* | NCBI | FJ639945.1 | - | - | - | - | - | - |
| Asteraceae | Asteroideae | Senecioneae | *Sinosenecio* | *Sinosenecio albonervius* | NCBI | JF978569.1 | JF956253.1 | - | JN047128.1 | JF944220.1 | KU750286.1 | KY970483.1 |
| Asteraceae | Asteroideae | Senecioneae | *Sinosenecio* | *Sinosenecio baojingensis* | NCBI | KU696080.1 | KT150049.1 | - | - | KT150055.1 | KU750287.1 | - |
| Asteraceae | Asteroideae | Senecioneae | *Sinosenecio* | *Sinosenecio bodinieri* | NCBI | KU696081.1 | KT149932.1 | AY723245.1 | JN047129.1 | KT150007.1 | KU750288.1 | KY970468.1 |
| Asteraceae | Asteroideae | Senecioneae | *Sinosenecio* | *Sinosenecio changii* | NCBI | KU696082.1 | KT150048.1 | - | - | KT150053.1 | KU750289.1 | - |
| Asteraceae | Asteroideae | Senecioneae | *Sinosenecio* | *Sinosenecio chienii* | NCBI | JF978574.1 | KT149952.1 | - | JN047131.1 | JF944224.1 | KU750290.1 | KY970477.1 |
| Asteraceae | Asteroideae | Senecioneae | *Sinosenecio* | *Sinosenecio confervifer* | NCBI | KU696086.1 | KT149936.1 | - | - | KT150011.1 | KU750292.1 | - |
| Asteraceae | Asteroideae | Senecioneae | *Sinosenecio* | *Sinosenecio cortusifolius* | NCBI | GU818710.1 | JF956275.1 | EU195525.1 | JN047148.1 | JF944242.1 | KU750298.1 | - |
| Asteraceae | Asteroideae | Senecioneae | *Sinosenecio* | *Sinosenecio cyclaminifolius* | NCBI | JF978577.1 | JF956261.1 | - | JN047133.1 | JF944227.1 | KU750293.1 | KY970384.1 |
| Asteraceae | Asteroideae | Senecioneae | *Sinosenecio* | *Sinosenecio denticulatus* | NCBI | JF978581.1 | JF956265.1 | - | JN047139.1 | JF944231.1 | KU750294.1 | KY970475.1 |
| Asteraceae | Asteroideae | Senecioneae | *Sinosenecio* | *Sinosenecio dryas* | NCBI | JF978584.1 | JF956268.1 | - | JN047141.1 | JF944234.1 | KU750295.1 | KY970480.1 |
| Asteraceae | Asteroideae | Senecioneae | *Sinosenecio* | *Sinosenecio eriopodus* | NCBI | KU696090.1 | KT149945.1 | - | - | KT150019.1 | KU750296.1 | KY970486.1 |
| Asteraceae | Asteroideae | Senecioneae | *Sinosenecio* | *Sinosenecio euosmus* | NCBI | GU818710.1 | JF956275.1 | EU195525.1 | JN047148.1 | JF944242.1 | KU750298.1 | - |
| Asteraceae | Asteroideae | Senecioneae | *Sinosenecio* | *Sinosenecio fangianus* | NCBI | KU696094.1 | KT150047.1 | - | - | KT150052.1 | KU750300.1 | - |
| Asteraceae | Asteroideae | Senecioneae | *Sinosenecio* | *Sinosenecio fanjingshanicus* | NCBI | KU696096.1 | KT149929.1 | - | - | KT150054.1 | KU750301.1 | - |
| Asteraceae | Asteroideae | Senecioneae | *Sinosenecio* | *Sinosenecio globiger* | NCBI | KT150041.1 | KT149939.1 | AY723247.1 | JN047150.1 | KT150014.1 | KU750304.1 | KY970394.1 |
| Asteraceae | Asteroideae | Senecioneae | *Sinosenecio* | *Sinosenecio guangxiensis* | NCBI | JF978599.1 | KT149923.1 | EU195526.1 | JN047158.1 | JF944255.1 | KU750307.1 | KY970476.1 |
| Asteraceae | Asteroideae | Senecioneae | *Sinosenecio* | *Sinosenecio hederifolius* | NCBI | KU696111.1 | KT149946.1 | EU195527.1 | - | KT150056.1 | KU750309.1 | - |
| Asteraceae | Asteroideae | Senecioneae | *Sinosenecio* | *Sinosenecio homogyniphyllus* | NCBI | JF978603.1 | KT149951.1 | EU195528.1 | JN047161.1 | KT150026.1 | KU750310.1 | KY970424.1 |
| Asteraceae | Asteroideae | Senecioneae | *Sinosenecio* | *Sinosenecio hupingshanensis* | NCBI | KU696115.1 | KT149940.1 | - | - | KT150015.1 | KU750311.1 | KY970474.1 |
| Asteraceae | Asteroideae | Senecioneae | *Sinosenecio* | *Sinosenecio jiangxiensis* | NCBI | JQ797424.1 | KT149922.1 | - | - | KT149999.1 | KU750312.1 | - |
| Asteraceae | Asteroideae | Senecioneae | *Sinosenecio* | *Sinosenecio jishouensis* | NCBI | KT149901.1 | KT149943.1 | - | - | KT150018.1 | KU750313.1 | - |
| Asteraceae | Asteroideae | Senecioneae | *Sinosenecio* | *Sinosenecio jiuhuashanicus* | NCBI | JQ797426.1 | JF956295.1 | - | JN047166.1 | JF944264.1 | KU750316.1 | KY970470.1 |
| Asteraceae | Asteroideae | Senecioneae | *Sinosenecio* | *Sinosenecio latouchei* | NCBI | JF978612.1 | JF956302.1 | - | JN047172.1 | JF944270.1 | KU750318.1 | KY970472.1 |
| Asteraceae | Asteroideae | Senecioneae | *Sinosenecio* | *Sinosenecio leiboensis* | NCBI | KU696118.1 | KT149926.1 | - | - | KT150002.1 | KU750319.1 | - |
| Asteraceae | Asteroideae | Senecioneae | *Sinosenecio* | *Sinosenecio ligularioides* | NCBI | JF978615.1 | JF956304.1 | - | JN047176.1 | JF944273.1 | KU750320.1 | KY970481.1 |
| Asteraceae | Asteroideae | Senecioneae | *Sinosenecio* | *Sinosenecio nanchuanicus* | NCBI | KU696120.1 | KT149937.1 | - | - | KT150012.1 | KU750321.1 | - |
| Asteraceae | Asteroideae | Senecioneae | *Sinosenecio* | *Sinosenecio oldhamianus* | NCBI | MH711715.1 | KT150046.1 | EU195529.1 | JN047178.1 | KT150051.1 | KU750323.1 | KY970561.1 |
| Asteraceae | Asteroideae | Senecioneae | *Sinosenecio* | *Sinosenecio palmatisectus* | NCBI | JF978621.1 | JF956311.1 | - | JN047181.1 | JF944279.1 | KU750325.1 | KY970473.1 |
| Asteraceae | Asteroideae | Senecioneae | *Sinosenecio* | *Sinosenecio rotundifolius* | NCBI | KU696126.1 | KT149949.1 | - | - | KT150024.1 | KU750326.1 | - |
| Asteraceae | Asteroideae | Senecioneae | *Sinosenecio* | *Sinosenecio saxatilis* | NCBI | KT149882.1 | KT149924.1 | - | - | KT150000.1 | KU750327.1 | - |
| Asteraceae | Asteroideae | Senecioneae | *Sinosenecio* | *Sinosenecio septilobus* | NCBI | JF978624.1 | JF956314.1 | EU195530.1 | JN047187.1 | JF944282.1 | KU750328.1 | KY970469.1 |
| Asteraceae | Asteroideae | Senecioneae | *Sinosenecio* | *Sinosenecio sichuanicus* | NCBI | JF978626.1 | JF956316.1 | - | JN047189.1 | JF944284.1 | KU750329.1 | KY970485.1 |
| Asteraceae | Asteroideae | Senecioneae | *Sinosenecio* | *Sinosenecio subrosulatus* | NCBI | KU696129.1 | JF956323.1 | - | JN047194.1 | KT150027.1 | KU750331.1 | KY970479.1 |
| Asteraceae | Asteroideae | Senecioneae | *Sinosenecio* | *Sinosenecio sungpanensis* | NCBI | JF978633.1 | JF956326.1 | - | JN047197.1 | JF944293.1 | KU750332.1 | KY970471.1 |
| Asteraceae | Asteroideae | Senecioneae | *Sinosenecio* | *Sinosenecio villifer* | NCBI | JF978635.1 | JF956328.1 | - | JN047200.1 | JF944296.1 | KU750333.1 | KY970383.1 |
| Asteraceae | Asteroideae | Senecioneae | *Sinosenecio* | *Sinosenecio wuyiensis* | NCBI | KT149878.1 | KT149919.1 | - | - | KT149996.1 | KU750334.1 | - |
| Asteraceae | Asteroideae | Senecioneae | *Sinosenecio* | *Sinosenecio yilingii* | NCBI | JF978638.1 | KT149955.1 | - | JN047203.1 | KT150030.1 | KU750335.1 | KY970482.1 |
| Asteraceae | Asteroideae | Millieae | *Smallanthus* | *Smallanthus sonchifolius* | NCBI | KF826287.1 | - | - | - | - | - | - |
| Asteraceae | Asteroideae | Millieae | *Smallanthus* | *Smallanthus uvedalia* | NCBI | MH984871.1 | - | - | MH985542.1 | KJ773900.1 | - | - |
| Asteraceae | Asteroideae | Astereae | *Solidago* | *Solidago altissima* | NCBI | HQ142590.1 | EU749409.1 | - | EU750560.1 | EU677019.1 | AB908050.1 | - |
| Asteraceae | Asteroideae | Astereae | *Solidago* | *Solidago canadensis* | NCBI | MZ005459.1 | MF159464.1 | - | EU337694.1 | MN601471.1 | KX243185.1 | - |
| Asteraceae | Asteroideae | Astereae | *Solidago* | *Solidago decurrens* | NCBI | MN947289.1 | MT384665.1 | - | KX346953.2 | - | - | - |
| Asteraceae | Asteroideae | Astereae | *Solidago* | *Solidago rugosa* | NCBI | HQ142588.1 | EU749422.1 | - | JQ246439.1 | EU677029.1 | - | - |
| Asteraceae | Asteroideae | Anthemideae | *Soliva* | *Soliva anthemifolia* | NCBI | GU724286.1 | HM989766.1 | AF153667.1 | GU724255.1 | JQ933485.1 | - | - |
| Asteraceae | Cichorioideae | Cichorieae | *Sonchella* | *Sonchella dentata* | NCBI | LT721925.1 | LR743492.1 | - | - | - | - | - |
| Asteraceae | Cichorioideae | Cichorieae | *Sonchus* | *Sonchus asper* | NCBI | KY968828.1 | DQ508004.1 | - | HE966831.1 | HM850372.1 | - | - |
| Asteraceae | Cichorioideae | Cichorieae | *Sonchus* | *Sonchus oleraceus* | NCBI | MH364397.1 | DQ840449.1 | EU385206.1 | KF196112.1 | MN601475.1 | JN177476.1 | - |
| Asteraceae | Cichorioideae | Cichorieae | *Soroseris* | *Soroseris erysimoides* | NCBI | MH808212.1 | MG946728.1 | - | JN047244.1 | JF944480.1 | KF732177.1 | - |
| Asteraceae | Cichorioideae | Cichorieae | *Soroseris* | *Soroseris glomerata* | NCBI | JF978804.1 | MG946744.1 | - | JN047248.1 | JF944489.1 | - | - |
| Asteraceae | Cichorioideae | Cichorieae | *Soroseris* | *Soroseris hookeriana* | NCBI | MK043358.1 | MG946751.1 | - | MG932921.1 | MN185091.1 | MK046061.1 | - |
| Asteraceae | Cichorioideae | Cichorieae | *Soroseris* | *Soroseris teres* | NCBI | HQ436221.1 | MG946753.1 | - | MG932925.1 | - | - | - |
| Asteraceae | Cichorioideae | Cichorieae | *Soroseris* | *Soroseris umbrella* | NCBI | HQ436198.1 | KF195985.1 | - | KF196117.1 | KF196029.1 | - | - |
| Asteraceae | Asteroideae | Inuleae | *Sphaeranthus* | *Sphaeranthus africanus* | NCBI | LN607569.1 | - | LN607695.1 | - | - | - | - |
| Asteraceae | Asteroideae | Inuleae | *Sphaeranthus* | *Sphaeranthus indicus* | NCBI | LN607576.1 | - | LN607734.1 | MG947106.1 | JQ933489.1 | - | - |
| Asteraceae | Asteroideae | Heliantheae | *Sphagneticola* | *Sphagneticola calendulacea* | NCBI | JQ064971.1 | - | - | - | - | - | - |
| Asteraceae | Asteroideae | Heliantheae | *Sphagneticola* | *Sphagneticola trilobata* | NCBI | MH768111.1 | AY215861.1 | AF384783.3 | MH152103.1 | AY215178.1 | - | - |
| Asteraceae | Asteroideae | Anthemideae | *Stilpnolepis* | *Stilpnolepis centiflora* | NCBI | MF417003.1 | - | - | MF416967.1 | KX527544.1 | MF416972.1 | - |
| Asteraceae | Asteroideae | Anthemideae | *Stilpnolepis* | *Stilpnolepis intricata* | NCBI | EF577313.1 | - | - | KY312288.1 | - | - | - |
| Asteraceae | Asteroideae | Athroismeae | *Symphyllocarpus* | *Symphyllocarpus exilis* | NCBI | KY210091.1 | KX526583.1 | KY210131.1 | - | KX527501.1 | - | - |
| Asteraceae | Asteroideae | Astereae | *Symphyotrichum* | *Symphyotrichum ciliatum* | NCBI | KJ711891.1 | MG225216.1 | - | - | MG224077.1 | - | - |
| Asteraceae | Asteroideae | Astereae | *Symphyotrichum* | *Symphyotrichum retroflexum* | NCBI | JQ360396.1 | - | - | - | - | - | - |
| Asteraceae | Asteroideae | Astereae | *Symphyotrichum* | *Symphyotrichum subulatum* | NCBI | KY968950.1 | MF159428.1 | - | MF143788.1 | HM849800.1 | - | - |
| Asteraceae | Cichorioideae | Cichorieae | *Syncalathium* | *Syncalathium chrysocephalum* | NCBI | JF978839.1 | JF956589.1 | - | JN047304.1 | JF944557.1 | MK046062.1 | - |
| Asteraceae | Cichorioideae | Cichorieae | *Syncalathium* | *Syncalathium disciforme* | NCBI | JF978842.1 | MG946754.1 | - | JN047308.1 | JF944563.1 | KF732176.1 | - |
| Asteraceae | Cichorioideae | Cichorieae | *Syncalathium* | *Syncalathium kawaguchii* | NCBI | MK043360.1 | MG946755.1 | - | JN047310.1 | JF944567.1 | MK046063.1 | - |
| Asteraceae | Cichorioideae | Cichorieae | *Syncalathium* | *Syncalathium roseum* | NCBI | HQ436205.1 | JF956603.1 | - | JN047313.1 | JF944571.1 | - | - |
| Asteraceae | Asteroideae | Heliantheae | *Synedrella* | *Synedrella nodiflora* | NCBI | EF108404.1 | AY215866.1 | AF384788.3 | AY215612.1 | AY215183.1 | - | - |
| Asteraceae | Asteroideae | Senecioneae | *Syneilesis* | *Syneilesis aconitifolia* | NCBI | MH711197.1 | MK435754.1 | - | - | MN185092.1 | - | KY970551.1 |
| Asteraceae | Asteroideae | Senecioneae | *Syneilesis* | *Syneilesis australis* | NCBI | KY979027.1 | - | - | - | - | KU750336.1 | KY970382.1 |
| Asteraceae | Asteroideae | Senecioneae | *Synotis* | *Synotis erythropappa* | NCBI | MH117781.1 | KX526587.1 | KX526876.1 | MH117341.1 | KX527009.1 | KU750338.1 | - |
| Asteraceae | Asteroideae | Senecioneae | *Synotis* | *Synotis glomerata* | NCBI | EU331117.1 | AY554083.1 | - | MF290374.1 | - | - | - |
| Asteraceae | Asteroideae | Senecioneae | *Synotis* | *Synotis lucorum* | NCBI | KU696134.1 | - | AY723218.1 | - | - | - | - |
| Asteraceae | Asteroideae | Senecioneae | *Synotis* | *Synotis nagensium* | NCBI | AF459922.1 | KX526588.1 | KX526926.1 | EF538052.1 | KX527026.1 | - | - |
| Asteraceae | Asteroideae | Senecioneae | *Synotis* | *Synotis solidaginea* | NCBI | KX549952.1 | KX526589.1 | KX526884.1 | KX549928.1 | KX527008.1 | - | - |
| Asteraceae | Carduoideae | Cardueae | *Synurus* | *Synurus deltoides* | NCBI | MH808217.1 | MK435755.1 | L39415.1 | - | - | - | AY865235.1 |
| Asteraceae | Carduoideae | Cardueae | *Syreitschikovia* | *Syreitschikovia tenuifolia* | NCBI | FJ007891.1 | - | - | - | - | - | - |
| Asteraceae | Asteroideae | Tageteae | *Tagetes* | *Tagetes erecta* | NCBI | KJ525046.1 | AY215867.1 | L39466.1 | MK090093.1 | AY215184.1 | - | AY865266.1 |
| Asteraceae | Asteroideae | Tageteae | *Tagetes* | *Tagetes minuta* | NCBI | MH231492.2 | - | AF405271.1 | - | MF694781.1 | - | - |
| Asteraceae | Asteroideae | Anthemideae | *Tanacetum* | *Tanacetum cinerariifolium* | NCBI | EF577319.1 | - | EU334486.1 | AB683390.1 | MT680902.1 | - | - |
| Asteraceae | Asteroideae | Anthemideae | *Tanacetum* | *Tanacetum coccineum* | NCBI | KY397500.1 | - | - | AB683391.1 | MG223704.1 | - | - |
| Asteraceae | Asteroideae | Anthemideae | *Tanacetum* | *Tanacetum vulgare* | NCBI | ON685464.1 | JN894898.1 | EU334490.1 | KM224631.1 | KM361004.1 | - | - |
| Asteraceae | Cichorioideae | Cichorieae | *Taraxacum* | *Taraxacum album* | NCBI | EU637114.1 | - | - | - | - | - | - |
| Asteraceae | Cichorioideae | Cichorieae | *Taraxacum* | *Taraxacum armeriifolium* | NCBI | EU637120.1 | - | - | - | - | - | - |
| Asteraceae | Cichorioideae | Cichorieae | *Taraxacum* | *Taraxacum aurantiacum* | NCBI | KC312164.1 | - | - | - | - | - | - |
| Asteraceae | Cichorioideae | Cichorieae | *Taraxacum* | *Taraxacum bessarabicum* | NCBI | MN480891.1 | - | - | - | - | - | - |
| Asteraceae | Cichorioideae | Cichorieae | *Taraxacum* | *Taraxacum bicorne* | NCBI | KY552478.1 | - | - | - | - | - | - |
| Asteraceae | Cichorioideae | Cichorieae | *Taraxacum* | *Taraxacum candidatum* | NCBI | EU637134.1 | - | - | - | - | - | - |
| Asteraceae | Cichorioideae | Cichorieae | *Taraxacum* | *Taraxacum coreanum* | NCBI | MG519655.1 | KF739654.1 | - | KU382755.1 | MG564451.1 | KX984936.1 | - |
| Asteraceae | Cichorioideae | Cichorieae | *Taraxacum* | *Taraxacum dasypodum* | NCBI | MH117786.1 | MH116897.1 | - | MH117347.1 | MH116446.1 | - | - |
| Asteraceae | Cichorioideae | Cichorieae | *Taraxacum* | *Taraxacum formosanum* | NCBI | AY862577.1 | - | - | - | - | - | - |
| Asteraceae | Cichorioideae | Cichorieae | *Taraxacum* | *Taraxacum glaucophyllum* | NCBI | EU637164.1 | - | - | - | - | - | - |
| Asteraceae | Cichorioideae | Cichorieae | *Taraxacum* | *Taraxacum lugubre* | NCBI | MH808219.1 | MK435756.1 | - | - | - | - | - |
| Asteraceae | Cichorioideae | Cichorieae | *Taraxacum* | *Taraxacum luridum* | NCBI | EU637200.1 | - | - | - | - | - | - |
| Asteraceae | Cichorioideae | Cichorieae | *Taraxacum* | *Taraxacum minutilobum* | NCBI | KF437435.1 | - | - | - | - | - | - |
| Asteraceae | Cichorioideae | Cichorieae | *Taraxacum* | *Taraxacum mitalii* | NCBI | KF437458.1 | - | - | - | - | - | - |
| Asteraceae | Cichorioideae | Cichorieae | *Taraxacum* | *Taraxacum mongolicum* | NCBI | MH711010.1 | MK090005.1 | - | MG947159.1 | JN407256.1 | KX984928.1 | - |
| Asteraceae | Cichorioideae | Cichorieae | *Taraxacum* | *Taraxacum niveum* | NCBI | EU637221.1 | - | - | - | - | - | - |
| Asteraceae | Cichorioideae | Cichorieae | *Taraxacum* | *Taraxacum nutans* | NCBI | KF437460.1 | - | - | - | - | - | - |
| Asteraceae | Cichorioideae | Cichorieae | *Taraxacum* | *Taraxacum sherriffii* | NCBI | EU637265.1 | - | - | - | - | - | - |
| Asteraceae | Cichorioideae | Cichorieae | *Taraxacum* | *Taraxacum sinicum* | NCBI | EU637277.1 | - | - | - | - | - | - |
| Asteraceae | Cichorioideae | Cichorieae | *Taraxacum* | *Taraxacum tibetanum* | NCBI | GU444020.1 | - | - | - | - | - | - |
| Asteraceae | Asteroideae | Senecioneae | *Tephroseris* | *Tephroseris flammea* | NCBI | KU696137.1 | - | - | - | - | KU750339.1 | KY970560.1 |
| Asteraceae | Asteroideae | Senecioneae | *Tephroseris* | *Tephroseris kirilowii* | NCBI | MH711065.1 | MH659514.1 | EU195532.1 | - | MH658029.1 | - | - |
| Asteraceae | Asteroideae | Senecioneae | *Tephroseris* | *Tephroseris koreana* | NCBI | JF978608.1 | JF956298.1 | - | JN047169.1 | JF944266.1 | KU750340.1 | KY970484.1 |
| Asteraceae | Asteroideae | Senecioneae | *Tephroseris* | *Tephroseris palustris* | NCBI | MW779519.1 | MK926079.1 | - | - | MG224495.1 | KU750341.1 | - |
| Asteraceae | Asteroideae | Senecioneae | *Tephroseris* | *Tephroseris pseudosonchus* | NCBI | KU696139.1 | - | - | - | - | - | - |
| Asteraceae | Asteroideae | Senecioneae | *Tephroseris* | *Tephroseris rufa* | NCBI | AY176166.1 | - | AY723244.1 | - | - | KU750343.1 | KY970557.1 |
| Asteraceae | Asteroideae | Heliantheae | *Tithonia* | *Tithonia diversifolia* | NCBI | MH050186.1 | MH070464.1 | - | - | MH049958.1 | - | - |
| Asteraceae | Cichorioideae | Cichorieae | *Tragopogon* | *Tragopogon altaicus* | NCBI | KF050361.1 | - | - | - | - | - | - |
| Asteraceae | Cichorioideae | Cichorieae | *Tragopogon* | *Tragopogon capitatus* | NCBI | AJ633493.1 | - | - | - | - | - | - |
| Asteraceae | Cichorioideae | Cichorieae | *Tragopogon* | *Tragopogon dubius* | NCBI | OL826782.1 | AJ633258.1 | - | HQ596868.1 | JX848433.1 | - | EU392047.1 |
| Asteraceae | Cichorioideae | Cichorieae | *Tragopogon* | *Tragopogon elongatus* | NCBI | AY645815.1 | - | - | - | - | - | - |
| Asteraceae | Cichorioideae | Cichorieae | *Tragopogon* | *Tragopogon gracilis* | NCBI | KF050408.1 | - | - | EU124026.1 | - | - | EU124034.1 |
| Asteraceae | Cichorioideae | Cichorieae | *Tragopogon* | *Tragopogon kasachstanicus* | NCBI | KF050415.1 | - | - | - | - | - | - |
| Asteraceae | Cichorioideae | Cichorieae | *Tragopogon* | *Tragopogon marginifolius* | NCBI | KF050428.1 | - | - | EU391974.1 | - | - | EU392036.1 |
| Asteraceae | Cichorioideae | Cichorieae | *Tragopogon* | *Tragopogon montanus* | NCBI | AY508172.1 | - | - | - | - | - | - |
| Asteraceae | Cichorioideae | Cichorieae | *Tragopogon* | *Tragopogon porrifolius* | NCBI | FN675710.1 | AJ633261.1 | L39391.1 | EF374280.1 | MN192552.1 | GQ984037.1 | EU392028.1 |
| Asteraceae | Cichorioideae | Cichorieae | *Tragopogon* | *Tragopogon pseudomajor* | NCBI | KF050444.1 | - | - | - | - | - | - |
| Asteraceae | Cichorioideae | Cichorieae | *Tragopogon* | *Tragopogon ruber* | NCBI | KF050447.1 | - | - | EF374287.1 | - | - | EU392063.1 |
| Asteraceae | Cichorioideae | Cichorieae | *Tragopogon* | *Tragopogon sabulosus* | NCBI | KF050450.1 | - | - | - | - | - | - |
| Asteraceae | Cichorioideae | Cichorieae | *Tragopogon* | *Tragopogon sibiricus* | NCBI | KF050454.1 | - | - | - | - | - | - |
| Asteraceae | Cichorioideae | Cichorieae | *Tragopogon* | *Tragopogon subalpinus* | NCBI | EU124009.1 | - | - | EU124025.1 | - | - | EU124033.1 |
| Asteraceae | Carduoideae | Cardueae | *Tricholepis* | *Tricholepis tibetica* | NCBI | AY826341.1 | - | JF754866.1 | - | - | - | - |
| Asteraceae | Asteroideae | Millieae | *Tridax* | *Tridax procumbens* | NCBI | EF108393.1 | MH621606.1 | - | MH622133.1 | JQ933511.1 | - | - |
| Asteraceae | Asteroideae | Anthemideae | *Tripleurospermum* | *Tripleurospermum inodorum* | NCBI | JF907423.1 | JN895475.1 | AF153656.1 | EU547795.1 | KM361022.1 | - | - |
| Asteraceae | Carduoideae | Cardueae | *Tugarinovia* | *Tugarinovia mongolica* | NCBI | EF627048.1 | AY785124.1 | KC590006.1 | MK299505.1 | KC589904.1 | - | - |
| Asteraceae | Asteroideae | Astereae | *Turczaninovia* | *Turczaninovia fastigiata* | NCBI | LC482289.1 | - | - | LC484269.1 | - | - | - |
| Asteraceae | Asteroideae | Senecioneae | *Tussilago* | *Tussilago farfara* | NCBI | MH711019.1 | JN896028.1 | AY723239.1 | HQ596875.1 | KM361024.1 | KU750345.1 | KY970556.1 |
| Asteraceae | Cichorioideae | Vernonieae | *Vernonia* | *Vernonia arborea* | NCBI | HQ158411.1 | JN837396.1 | EF155686.1 | - | MG707329.1 | - | - |
| Asteraceae | Cichorioideae | Vernonieae | *Vernonia* | *Vernonia attenuata* | NCBI | HQ158371.1 | - | HQ158472.1 | - | - | - | - |
| Asteraceae | Cichorioideae | Vernonieae | *Vernonia* | *Vernonia cinerea* | NCBI | EF107650.1 | LC503588.1 | HQ158497.1 | LC503590.1 | LC503589.1 | - | - |
| Asteraceae | Cichorioideae | Vernonieae | *Vernonia* | *Vernonia cumingiana* | NCBI | HG004807.1 | HG004925.1 | - | MH837925.1 | KF181498.1 | - | - |
| Asteraceae | Cichorioideae | Vernonieae | *Vernonia* | *Vernonia divergens* | NCBI | HQ158373.1 | - | HQ158476.1 | - | - | - | - |
| Asteraceae | Cichorioideae | Vernonieae | *Vernonia* | *Vernonia elliptica* | NCBI | HQ158414.1 | MG712611.1 | HQ158517.1 | - | MW393548.1 | - | - |
| Asteraceae | Cichorioideae | Vernonieae | *Vernonia* | *Vernonia saligna* | NCBI | HQ158379.1 | - | HQ158482.1 | - | - | - | - |
| Asteraceae | Cichorioideae | Vernonieae | *Vernonia* | *Vernonia silhetensis* | NCBI | HQ158380.1 | - | HQ158483.1 | - | - | - | - |
| Asteraceae | Cichorioideae | Vernonieae | *Vernonia* | *Vernonia solanifolia* | NCBI | HQ158412.1 | - | HQ158516.1 | - | - | - | - |
| Asteraceae | Asteroideae | Heliantheae | *Wollastonia* | *Wollastonia biflora* | NCBI | MH768115.1 | AY297653.1 | - | MH152119.1 | MH767508.1 | - | - |
| Asteraceae | Asteroideae | Heliantheae | *Wollastonia* | *Wollastonia montana* | NCBI | JQ065017.1 | - | - | - | - | - | - |
| Asteraceae | Asteroideae | Heliantheae | *Xanthium* | *Xanthium spinosum* | NCBI | KY215702.1 | HM850669.1 | - | KY215739.1 | HM850472.1 | - | - |
| Asteraceae | Asteroideae | Heliantheae | *Xanthium* | *Xanthium strumarium* | NCBI | ON505999.1 | KX272487.1 | - | DQ006156.1 | MH203382.1 | - | - |
| Asteraceae | Carduoideae | Cardueae | *Xanthopappus* | *Xanthopappus subacaulis* | NCBI | AY914833.1 | KC590039.1 | KC590008.1 | AY914851.1 | KC589907.1 | - | - |
| Asteraceae | Asteroideae | Gnaphalieae | *Xerochrysum* | *Xerochrysum bracteatum* | NCBI | KY397488.1 | AF318905.1 | AB530935.1 | KF263792.1 | AB530969.1 | - | - |
| Asteraceae | Cichorioideae | Cichorieae | *Youngia* | *Youngia cineripappa* | NCBI | KJ502307.1 | - | - | LT722068.1 | - | KC968136.1 | - |
| Asteraceae | Cichorioideae | Cichorieae | *Youngia* | *Youngia conjunctiva* | NCBI | KC968036.1 | - | - | - | - | KC968125.1 | - |
| Asteraceae | Cichorioideae | Cichorieae | *Youngia* | *Youngia erythrocarpa* | NCBI | MN701100.1 | KF739655.1 | - | KF739628.1 | - | KF732169.1 | - |
| Asteraceae | Cichorioideae | Cichorieae | *Youngia* | *Youngia fusca* | NCBI | KR733613.1 | - | - | - | - | KR733621.1 | - |
| Asteraceae | Cichorioideae | Cichorieae | *Youngia* | *Youngia gracilipes* | NCBI | KJ502308.1 | - | - | - | - | KC968126.1 | - |
| Asteraceae | Cichorioideae | Cichorieae | *Youngia* | *Youngia henryi* | NCBI | MH808222.1 | MK435757.1 | - | - | - | KF732133.1 | - |
| Asteraceae | Cichorioideae | Cichorieae | *Youngia* | *Youngia heterophylla* | NCBI | MN701103.1 | KF195987.1 | - | KF196119.1 | KC967988.1 | KC968123.1 | - |
| Asteraceae | Cichorioideae | Cichorieae | *Youngia* | *Youngia japonica* | NCBI | MH711060.1 | EU385409.1 | EU385218.1 | KP402578.1 | EU385029.1 | KC968091.1 | - |
| Asteraceae | Cichorioideae | Cichorieae | *Youngia* | *Youngia paleacea* | NCBI | MH117809.1 | KF195988.1 | - | MH117371.1 | KC968019.1 | KF732135.1 | - |
| Asteraceae | Cichorioideae | Cichorieae | *Youngia* | *Youngia rubida* | NCBI | KC968042.1 | - | - | - | - | KC968095.1 | - |
| Asteraceae | Cichorioideae | Cichorieae | *Youngia* | *Youngia simulatrix* | NCBI | KJ502312.1 | MH293250.1 | - | - | - | - | - |
| Asteraceae | Cichorioideae | Cichorieae | *Youngia* | *Youngia zhengyiana* | NCBI | KJ502314.1 | - | - | - | - | - | - |
| Asteraceae | Asteroideae | Heliantheae | *Zinnia* | *Zinnia peruviana* | NCBI | KY989553.1 | MT214875.1 | - | MF037529.1 | MH028872.1 | - | EU586887.1 |
