# Supplementary material for: Spatial Phylogenetics Reveals Endemism Hotspots and Conservation Priorities in Chinese Asteraceae
Source: Ecol Evol. 2025 Nov 2;15(11):e72403. doi: 10.1002/ece3.72403 (PMC12579969; doi:10.1002/ece3.72403)

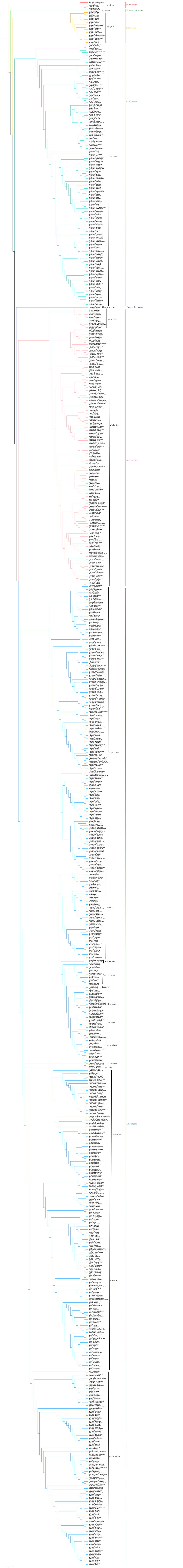

Supplement: Supplementary file 1 — Figure S1: Phylogenetic tree of Asteraceae in China. 7 subfamilies and 22 tribes are shown. [file ECE3-15-e72403-s001.pdf]
